# Loss of STARD7 Triggers Metabolic Reprogramming and Cell Cycle Arrest in Breast Cancer

**DOI:** 10.1002/advs.202503022

**Published:** 2025-05-30

**Authors:** Ewelina Dondajewska, Paula Allepuz‐Fuster, Chloé Maurizy, Alexandre Hego, Sandra Ormenese, Quentin Lion, Arnaud Blomme, Pierre Close, Arnaud Lavergne, Latifa Karim, Marc Thiry, Ivan Nemazanyy, Roopesh Krishnankutty, Jair Marques, Alex von Kriegsheim, Nathaniel F. Henneman, Ganna Panasyuk, Kateryna Shostak, Alain Chariot

**Affiliations:** ^1^ Laboratory of Cancer Biology Liège Belgium; ^2^ GIGA Cancer GIGA University of Liege CHU Sart‐Tilman Liège 4000 Belgium; ^3^ Department of Medical Biotechnology Poznan University of Medical Sciences (PUMS) Poznan Poland; ^4^ GIGA Flow Cytometry and Cell Imaging Platform University of Liege Liege Belgium; ^5^ Laboratory of Cancer Signaling Liège Belgium; ^6^ WELBIO department WEL Research Institute avenue Pasteur, 6, 1300 Wavre Belgium; ^7^ GIGA Genomics Platform University of Liege Liege Belgium; ^8^ Unit of Cell and Tissue Biology GIGA Neurosciences University of Liege Liege Belgium; ^9^ Platform for Metabolic Analyses Structure Fédérative de Recherche Necker INSERM US24/CNRS UAR 3633 Paris 75015 France; ^10^ Institute of Genetics and Cancer Edinburgh Cancer Research Scotland; ^11^ Institut Necker‐Enfants Malades (INEM) INSERM U1151/CNRS UMR 8253, 75015 Paris France and Université de Paris Cité Paris 75006 France

**Keywords:** breast cancer, cell cycle arrest, EGFR signaling, lipid transfer protein, metabolic reprogramming

## Abstract

Cancer cells adapt their metabolism to support aberrant cell proliferation. However, the functional link between metabolic reprogramming and cell cycle progression remains largely unexplored. Mitochondria rely on the transfer of multiple lipids from the endoplasmic reticulum (ER) to their membranes to be functional. Several mitochondrial‐derived metabolites influence cancer cell proliferation by modulating the epigenome. Here, the loss of STARD7, a lipid transfer protein whose expression is enhanced in breast cancer, is shown to lead a metabolic reprogramming characterized by the accumulation of carnitine derivatives and S‐Adenosyl‐L‐methionine (SAM). Elevated SAM levels cause the increase of H3K27 trimethylation on many gene promoters coding for candidates involved in cell cycle progression. Likewise, STARD7 deficiency triggers cell cycle arrest and impairs ERα‐dependent cell proliferation. Moreover, EGFR signaling is also impaired in triple negative breast cancer cells lacking STARD7, at least due to deregulated EGFR trafficking to lysosomes. Therefore, mitochondria rely on STARD7 to support cell cycle progression in breast cancer.

## Graphical abstract

Breast cancer cells undergo metabolic and transcriptomic reprogramming to support aberrant cell proliferation. Their mitochondria rely on the transfer of phosphatidylcholine from the endoplasmic reticulum to their membranes by STARD7, a candidate upregulated in breast cancer, to be functional. The loss of STARD7 leads to enhanced S‐Adenosyl‐L‐methionine (SAM) levels, which ultimately impairs ERα‐ and EGFR‐dependent signaling pathways through epigenetic mechanisms.

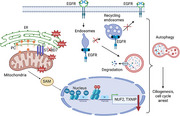

## 1 Introduction

The maintenance of membrane homeostasis relies on an intense exchange of lipids between cellular membranes.^[^ ^1^ ^]^ Most lipids are synthesized within the endoplasmic reticulum (ER) and redistributed from there to other cellular membranes, including mitochondrial membranes. Mitochondria carry out several essential functions, such as ATP production, calcium regulation, and redox homeostasis.^[^ ^2^, ^3^ ^]^

STARD7, a lipid transfer protein belonging to the family of START (steroidogenic acute regulatory protein‐related lipid transfer) domain superfamily, facilitates the transfer of lipids among intracellular membranes^[^ ^4^ ^]^ (see our supplementary file for the list of all abbreviations used in this study). STARD7 preferentially binds to phosphatidylcholine (PC) and promotes the delivery of PC to the mitochondria.^[^ ^5^ ^]^ Consistently, STARD7‐deficient cells show a reduction of mitochondrial PC as well as a disorganized cristae structure of mitochondria.^[^ ^6^ ^]^ This altered mitochondrial structure is associated with a decreased aerobic respiration, increased oxidative stress, and mitochondrial DNA damage, resulting in altered barrier integrity in vivo.^[^ ^7^ ^]^ STARD7 is actually localized in the intermembrane space and mitochondrial STARD7 is necessary and sufficient for PC accumulation in the inner membrane as well as for mitochondrial respiration and cristae morphogenesis.^[^ ^8^ ^]^ The inner membrane protease PARL (presenilin‐associated rhomboid‐like) can cleave STARD7, resulting in the release of mature STARD7 in the cytosol.^[^ ^8^ ^]^ Interestingly, cytosolic STARD7 is critical for the transport of Coenzyme Q, a redox‐active lipid acting as an universal electron carrier in the mitochondrial respiratory chain, to the plasma membrane and consequently protects from ferroptosis, suggesting that STARD7 function is not limited to PC transport.^[^ ^9^ ^]^

Members of the START‐domain containing protein family are aberrantly expressed in cancer. Indeed, STARD1, which promotes cholesterol trafficking to mitochondria, is overexpressed in nonalcoholic steatohepatitis (NASH)‐derived hepatocellular carcinoma (HCC) and critically contributes to HCC in mice.^[^ ^10^ ^]^ STARD3, another cholesterol‐binding protein, is part of the amplified locus with ERBB2 on human chromosome 17q12 found in both gastric and breast cancer.^[^ ^11^ ^]^ STARD3 contributes to breast cancer development, at least by promoting both Src and PI3K/AKT/mTOR activation.^[^ ^12^, ^13^ ^]^ STARD4, which facilitates the transfer of cholesterol to the cytoplasmic membrane, is overexpressed in breast cancer and promotes cell proliferation and migration.^[^ ^14^ ^]^ Finally, STARD10 is also overexpressed in breast cancer and cooperates with ErbB receptor signaling in cellular transformation.^[^ ^15^ ^]^ Likewise, ethanol abuse, which exacerbates breast cancer progression, induces both STARD10 and ERBB2 expression.^[^ ^16^ ^]^ Therefore, these studies undoubtedly established a link between aberrant expression of START protein family members and cancer development but underlying molecular mechanisms have not been extensively dissected yet.

We demonstrate here that STARD7 is overexpressed in ERα^+^ breast malignancies and in Triple Negative Breast Cancers (TNBCs). Loss of STARD7 in breast cancer cells causes a metabolic reprogramming characterized by the accumulation of several mitochondrial metabolites, including carnitine derivatives and S‐Adenosylmethionine (SAM). The defective transport of PC to mitochondria due to STARD7 deficiency also leads to increased numbers of mitochondria‐associated ER membranes (MAMs). Accumulation of SAMs in breast cancer cells lacking STARD7 enhances H3K27 methylation on genes coding for candidates involved in cell cycle progression. Loss of STARD7 consequently causes cell cycle arrest, at least through defective ERα‐ and EGFR‐dependent signaling pathways as well as autophagy. Collectively, our results provide molecular mechanisms through which cancer cells reprogram their metabolic and transcriptomic signatures to support sustained cell proliferation.

## 2 Results

### 2.1 STARD7 is Overexpressed in Breast Cancer

**Figure 1:**
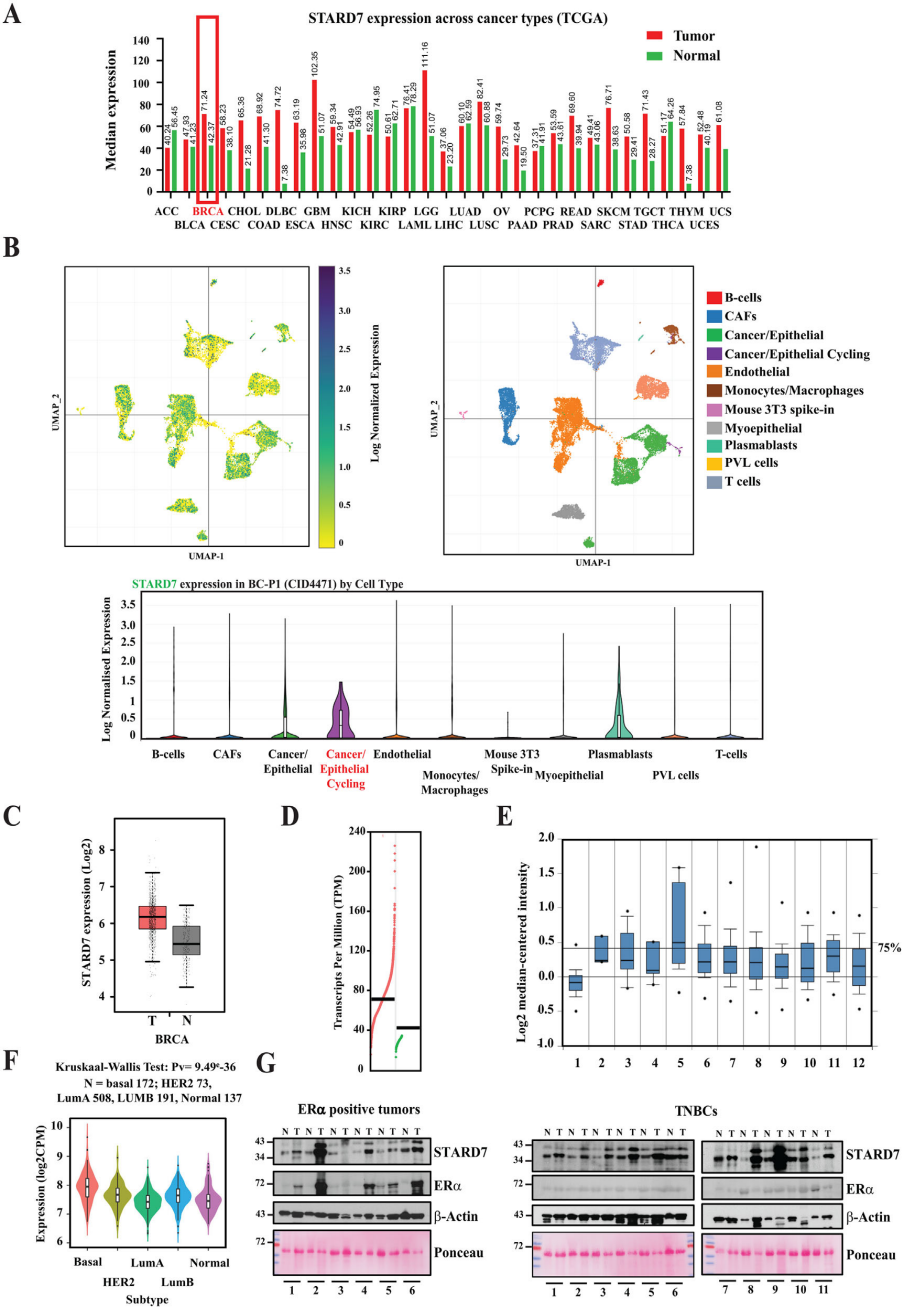
STARD7 overexpression in breast cancer. A) STARD7 expression in multiple human cancer types. TCGA data with mRNA expression levels of STARD7 in human cancer types are illustrated. B) STARD7 expression at the single cell level. Processed data were collected from.^[^ ^53^ ^]^ The UMAP clustering from breast tumor BC‐P1 (CID4471) is illustrated. C,D) Enrichment of STARD7 mRNAs in breast cancer versus normal adjacent tissues (TCGA data). E) STARD7 overexpression in human breast cancer. STARD7 mRNA levels were quantified in the following cases: 1) Breast (144), 2) Benign Breast Neoplasm (3), 3) Breast Carcinoma (14), 4) Breast Phyllodes Tumor (5), 5) Ductal Breast Carcinoma in Situ (10), 6) Invasive Breast Carcinoma (21), 7) Invasive Ductal and Invasive Lobular Breast Carcinoma (90), 8) Invasive Ductal Breast Carcinoma (1556), 9) Invasive Lobular Breast Carcinoma (148), 10) Medullary Breast Carcinoma (32), 11) Mucinous Breast Carcinoma (46), and 12) Tubular Breast Carcinoma (67), as described.^[^ ^54^ ^]^ F) Enrichment of STARD7 expression in the basal subtype of breast cancer. STARD7 expression in all indicated breast cancer subtypes was established using TISIDB (Tumor and Immune System Interaction Database) (http://cis.hku.hk/TISIDB/index.php).^[^ ^55^ ^]^ G) Enhanced STARD7 protein levels in human breast cancer. Extracts from ER^+^ and TNBCs as well as from corresponding adjacent normal tissues were subjected to Western blot (WB) analyses using the indicated antibodies.

To learn more on mechanisms through which lipid transfer proteins regulate tumor development, we first looked at the expression profile of STARD7 in human tumors. STARD7 was overexpressed in many human tumors, including cholangiocarcinoma (CHOL), cervical squamous cell carcinoma and endocervical adenocarcinoma (CESC), diffuse large B cell lymphoma (DLBCL), glioblastoma (GBM), pancreatic adenocarcinoma (PAAD), rectum adenocarcinoma (READ), skin cutaneous melanoma (SKMC), testicular germ cells tumor (TGCT), thymoma (THYM), and breast cancer (BRCA), as indicated by data generated by the TCGA Research Network (https://www.cancer.gov/tcga) (**Figure** **1**A). Interestingly data from single‐cell RNA analyses carried out on human breast cancers indicated that STARD7 expression was detected in multiple cell types, especially in cycling and transformed breast epithelial cells (Figure 1B). *STARD7* transcripts were indeed enriched in breast tumors versus normal adjacent tissues (Figure 1C,D). Interestingly, mRNA levels of *STARD7* were found elevated in benign breast neoplasms as well as in both ductal and invasive carcinomas when compared to levels in normal breast, suggesting that *STARD7* overexpression occurs early during tumor development (Figure 1E). STARD7 expression was also found to be enriched in the basal subtype of breast cancer (Figure 1F). STARD7 overexpression was also detected at the protein level in all our tested clinical cases of TNBCs as well as in most ER^+^ tumors (infiltrating ductal carcinomas) (Figure 1G). Of note, STARD7 expression was not correlated to patient survival nor to the stage of the disease, at least based on its mRNA levels (Figure S1A and S1B, respectively). Therefore, breast cancer development is characterized by elevated levels of STARD7.

### 2.2 Loss of STARD7 in Breast Cancer Cells Leads to Changes in Mitochondrial Morphology

**Figure 2:**
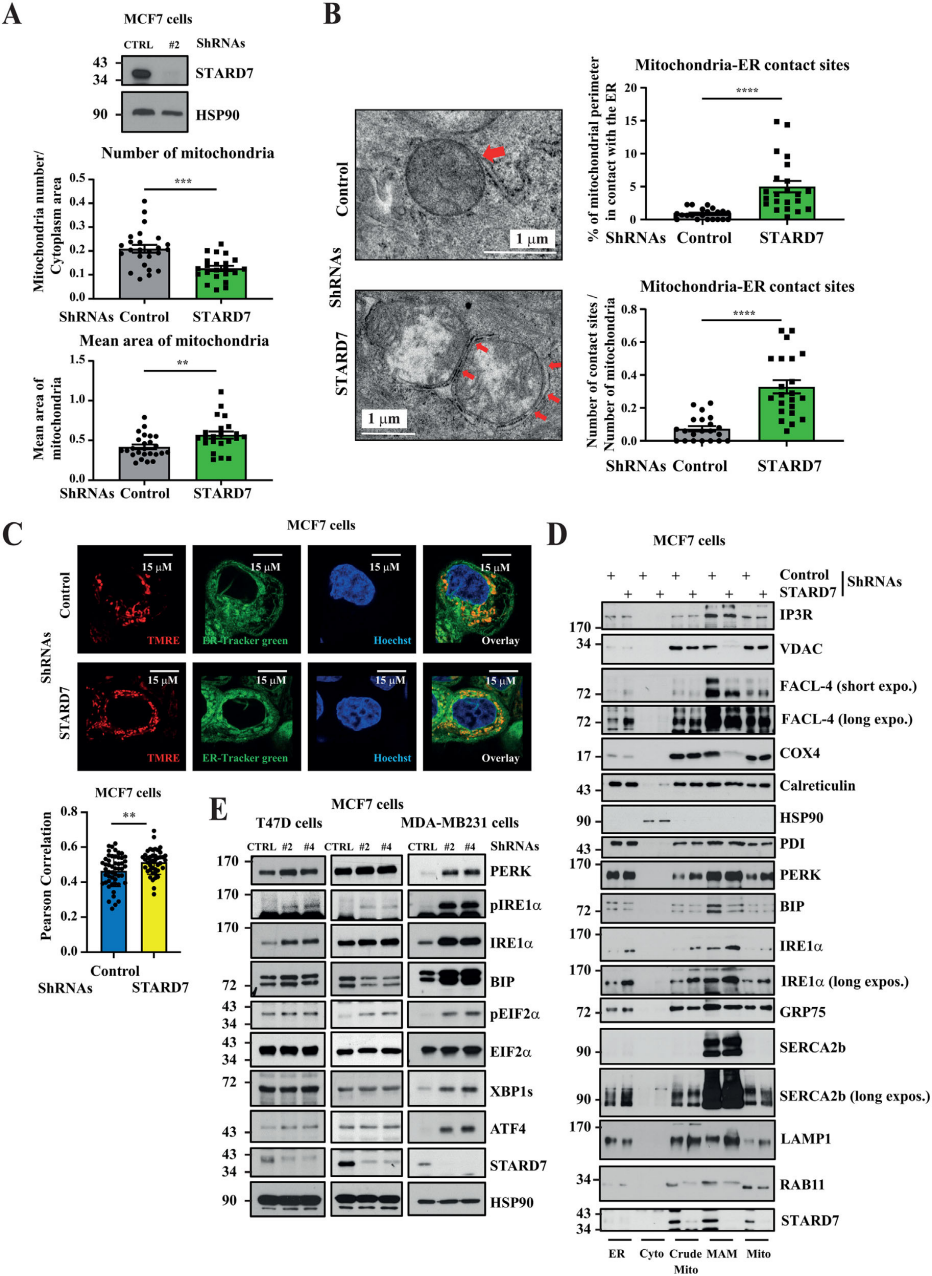
STARD7 deficiency leads to changes in mitochondrial morphology in breast cancer cells. A) Enhanced number and size of mitochondria upon STARD7 deficiency. A quantitative analysis of mitochondria observed by Transmission Electron Microscopy (TEM) in control and STARD7‐depleted breast cancer cells is illustrated. The average number of mitochondria by cytoplasmic area and the average area of mitochondria are shown (top and bottom histograms, respectively) (***p < 0.001). WB analyses using extracts from control and STARD7‐depleted MCF7 cells are also illustrated on the top. B,C) Enhanced mitochondria‐ER contact sites in breast cancer cells lacking STARD7. TEM analysis of mitochondria/ER contacts in control and STARD7‐depleted MCF7 cells (B). On the left, illustration of the ultrastructure of mitochondria/ER contacts in both control and STARD7‐depleted MCF7 cells. On the right, number of mitochondria/ER contact sites and average percentage of mitochondrial perimeter in contact with the ER are plotted (top and bottom histograms, respectively) (see methods for details on statistical analyses). Control and STARD7‐depleted MCF7 cells were co‐stained with the marker of active mitochondria TMRE and with ER‐Tracker green (Endoplasmatic Reticulum marker) in order to show contact sites between both mitochondria and ER (C). Hoechst stainings were done to show the nucleus. Illustrated graphs represent the degree of colocalization between ER and mitochondria, using the Pearson's coefficient. A total of 51 control and 43‐depleted MCF7 cells were analyzed. The Prism10 program was used for statistical analyses (Welch's *t*‐test, **p < 0.01). D) Enrichment of some but not all MAMs markers in MAMs upon STARD7 deficiency. Extracts from MCF7 cells were biochemically fractionated to generate organelle‐enriched extracts. ER = endoplasmic reticulum, Cyto = cytoplasm, Crude mito = crude mitochondria, MAM = mitochondria‐associated membrane, Mito = mitochondria. The organelle‐enriched extracts were subjected to Western blots (WB) using the indicated antibodies. E) Elevated levels of ER stress markers in breast cancer cell lacking STARD7. Extracts from control and STARD7‐depleted MCF7 cells were subjected to WB analyses using the indicated antibodies. Experiments were repeated at least three times. Representative blots are shown.

To define the biological processes controlled by STARD7 in breast cancer, we first assessed *STARD7* expression in a variety of human breast cancer cell lines using the DepMap dataset. We found that *STARD7* mRNA levels were comparable in TNBCs, ERα^+^ and ERBB2^+^ breast cancer cell lines (Figure S1C, left panel, Supporting Information). Likewise, Luminal A‐ and Luminal B‐derived breast cancer cell lines also showed comparable mRNA levels of *STARD7* (Figure S1C, right panel, Supporting Information). We next assessed STARD7 protein levels in several established breast cancer cell lines. Selected ERα‐positive cell lines included MCF7, ZR75‐1, and T47 cells. As expected, all three cell lines expressed high levels of the epithelial marker E‐Cadherin but did not express the mesenchymal marker Vimentin (Figure S1D, Supporting Information). HCC1569 breast cancer cells expressed high levels of the oncogenic protein HER2 but not ERα (Figure S1D, Supporting Information). ERα‐negative cell lines included HCC70, HCC1187 as well as MDA‐MB231 cells, all defined as Triple‐negative breast cancer cells (TNBC)‐derived cell lines (even if HER2 was nevertheless detected in HCC1187 cells). STARD7 expression was also assessed in the immortalized but nontumorigenic MCF10A cell line. We systematically detected high STARD7 protein levels in all tested breast cancer cell lines, although at lower levels in both MCF10A and T47D cell lines (Figure S1D, Supporting Information). To assess STARD7 biological functions in breast cancer, we first generated MCF7 cells lacking STARD7 (**Figure** **2**A). As PC transfer from the ER to mitochondria relies on STARD7, we looked at the consequences of STARD7 deficiency in both the shape and function of mitochondria. MCF7 cells lacking STARD7 had fewer but enlarged mitochondria (Figure 2A). Moreover, MCF7 cells lacking STARD7 exclusively showed orthodox mitochondria while control cells showed 39% of condensed organelles, which have a contraction of the matrix space (Figure S2A, Supporting Information). To clarify whether the increased size and decreased number of mitochondria were the result of increased mitochondria fusion, we next looked at levels of key mitochondria dynamics regulators. In this context, levels of Mitofusin 2, a key protein in mitochondrial fusion, Dynamin‐Related Protein 1 (DRP1), a dynamin‐related GTPase involved in mitochondrial division as well as Mitochondrial Fission 1 (FIS1) involved in mitochondrial outer membrane fission^[^ ^17^, ^18^, ^19^ ^]^ remained unchanged upon STARD7 deficiency (Figure S2B, Supporting Information).

Given the fact that STARD7 deficiency changes both mitochondrial number and size and because STARD7 promotes the transport of PC from the ER to mitochondria, we next looked at mitochondria‐ER contacts. We observed that cells lacking STARD7 displayed more contacts between both organelles (Figure 2B). Moreover, the percentage of mitochondrial perimeter in contact with the ER also increased upon STARD7 deficiency (Figure 2B). We also stained control versus STARD7‐depleted MCF7 with TMRE, a marker of active mitochondria and with ER‐Tracker green, an ER marker and confirmed that STARD7 deficiency led to more contact sites between both organelles (Figure 2C). This conclusion was also true in triple negative breast cancer‐derived MDA‐MB231 cells lacking STARD7 (Figure S2C, Supporting Information). Therefore, loss of STARD7 leads to higher numbers of mitochondria‐associated membranes (MAMs) in breast cancer cells, potentially as a compensatory mechanism of a defective PC transport between both organelles.

We biochemically fractionated extracts from both control and STARD7‐depleted MCF7 cells in order to assess whether STARD7 deficiency would have any consequence on the expression profile of candidates known to be found in MAMs. In this context, we noticed that IRE1α, an ER stress sensor also acting as a structural determinant of MAMs involved in the control of mitochondrial calcium uptake, but not BIP levels, increased in MAMs from STARD7‐depleted cells (Figure 2D). Of note, IRE1α levels also increased in additional cell compartments, including in both MAMs and mitochondrial extracts (Figure 2D). As expected, Sarcoplasmic/endoplasmic reticulum Ca^2+^‐ATPase 2b (SERCA2b) was found in the ER but also in MAMs from MCF7 cells^[^ ^20^ ^]^ but its level did not dramatically change upon STARD7 deficiency (Figure 2D). Moreover, Inositol 1,4,5‐trisphosphate receptors (IP3R), Voltage‐dependent anion channel 1 (VDAC1) and glucose‐regulated protein 75 (GRP75), which all form a complex to modulate ER‐mitochondria Calcium signaling,^[^ ^21^ ^]^ were also found in MAMs from MCF7 cells (Figure 2D). However, although both GRP75 and IP3R levels in MAMs did not change upon STARD7 deficiency, MCF7 cells lacking STARD7 showed dramatically decreased levels of VDAC1 in MAMs but not in other cell compartments (Figure 2D). This conclusion also applied to COX4 (Figure 2D). Consistently, levels of IRE1α, another candidate found in MAMs,^[^ ^22^ ^]^ were elevated in total extracts from T47D, MCF7, and MDA‐MB231 cells lacking STARD7 (Figure 2E). Of note, the ER stress inducer RNA‐dependent protein kinase (PKR)‐like ER kinase (PERK), which is enriched in MAMs,^[^ ^23^ ^]^ was found elevated upon STARD7 deficiency in all tested breast cancer cell lines, especially in MDA‐MB231 cells (Figure 2E). Therefore, STARD7 deficiency alters protein levels of many but not all proteins in MAMs. Given the fact that the ER stress inducer PERK was more expressed in breast cancer cells lacking STARD7, we also look at levels of other ER stress downstream targets such as Eukaryotic Initiation Factor 2α (EIF2α), X box‐binding protein 1 (XBP1) and Activating transcription factor 4 (ATF4). These candidates were robustly elevated upon STARD7 deficiency in MDA‐MB231 but not in both T47D and MCF7 cells, as were BIP levels (Figure 2E). Levels of the lysosomal protein LAMP1 were increased upon STARD7 deficiency in MAMs as well as in mitochondrial extracts (Figure 2D). Importantly, levels of RAB11, which is associated with recycling endosomes, were decreased upon STARD7 deficiency in both MAMs and mitochondrial extracts, suggesting that the recycling of some key membrane receptors such as EGFR may be impaired in cells lacking STARD7 (Figure 2D) Therefore, STARD7 deficiency has profound consequences on protein levels of multiple candidates known to be found in cellular membranes.

### 2.3 Loss of STARD7 Leads to a Metabolic and Epigenetic‐Mediated Transcriptional Reprogramming

**Figure 3:**
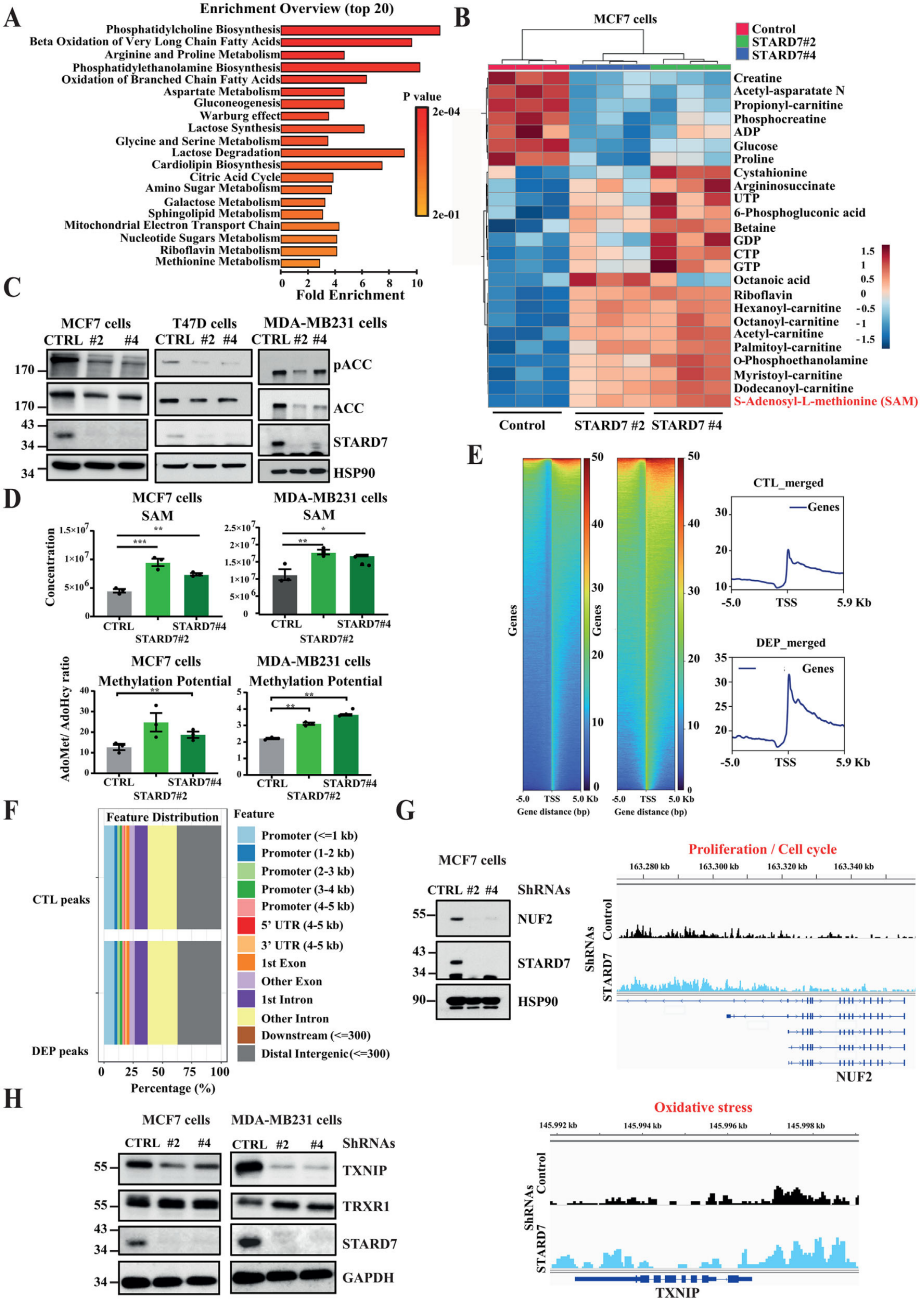
STARD7 deficiency leads to a metabolic and an epigenetic‐mediated transcriptional reprogramming. A) The metabolic signature of both control and STARD7‐depleted MCF7 cells was established on cells from three independent depletions (see methods for details). An Enrichment overview was conducted and the top 20 upregulated pathways in cells lacking STARD7 are illustrated. B) Enrichment of carnitine derivatives and S‐Adenosyl‐L‐methionine (SAM) in breast cancer cells lacking STARD7. A HeatMap was generated using the metabolomic data from control and STARD7‐depleted MCF7 cells. Metabolites whose level dramatically change upon STARD7 deficiency are illustrated. C) Defective ACC expression upon STARD7 deficiency in breast cancer cells. Extracts from MCF7, T47D or MDA‐MB231 cells were subjected to WB analyses using the indicated antibodies. Experiments were repeated at least three times. Representative blots are shown. D) Enhanced SAM levels and methylation potential in breast cancer cells lacking STARD7. The plotted data were extracted from the metabolomic data. *p < 0.05, **p < 0.01, ***p < 0.001). E,F) Enrichment of H3K27 trimethylation on gene candidates involved in cell proliferation and in the response to oxidative stress. H3K27me3 ChIP‐seq analysis of control and STARD7‐depleted MCF7 cells (Sh Control, « CTL » and sh STARD7#2, « DEP », respectively) were conducted on cells from three independent depletions. Peak profiles and heatmaps depicting genome‐wide H3K27me3 peaks aligned by intensity of reads surrounding the TSS (*n* = 3 merged experiments) are illustrated (E). Color key indicates relative intensity of peaks. Annotated feature distribution of H3K27me3 peaks in control and STARD7‐depleted MCF7 cells (« CTL » and « DEP », respectively) within respective genomic regions are shown (F). G,H) Gene tracks of H3K27me3 enrichment on NUF2 (G) and TXNIP (H) genes, respectively (*n* = 3 merged experiments). TSS‐transcription start site. Extracts from control and STARD7‐depleted MCF7 (G and H) and MDA‐MB231 (H) cells were subjected to WB analyses using the indicated antibodies for validation purposes. Experiments were repeated at least three times. Representative blots are shown.

To address consequences of STARD7 deficiency on mitochondrial functions, we performed LC‐MS metabolomics and established the metabolic signature of both control and STARD7‐depleted cells (**Figure** **3**A,B). An enrichment overview of these signatures revealed numerous deregulated metabolic pathways in breast cancer cells lacking STARD7 (Figure 3A). Fatty acids (FA) β‐oxidation was strongly upregulated in breast cancer cell lacking STARD7 (Figure 3A). Of note, levels of Acetyl‐CoA carboxylase (ACC), which catalyzes the carboxylation of acetyl‐CoA into malonyl‐CoA,^[^ ^24^ ^]^ were downregulated upon STARD7 deficiency in MCF7, T47D cells as well as in MDA‐MB231 cells (Figure 3C). As a consequence, its phosphorylated and inactivated form (pACC) was also downregulated upon STARD7 deficiency (Figure 3C). Interestingly, levels of acetyl‐carnitine, which is synthesized from acetyl‐CoA by carnitine palmitoyltransferase I (CPTI), the key limiting‐step of FA β‐oxidation,^[^ ^25^ ^]^ increased in MCF7 cells lacking STARD7 (Figure 3B). Likewise, STARD7 deficiency led to enhanced levels of a variety of carnitine derivatives, suggesting that mitochondrial capacity to oxidize FAs may be overwhelmed in these cells, which causes accumulation of toxic lipid intermediates (Figure 3B).^[^ ^26^ ^]^ Importantly, levels of the universal methyl donor for methylation reactions S‐Adenosyl‐L‐methionine (SAM) were also elevated in MCF7 cells lacking STARD7 (Figure 3B,D). Such observation was also seen upon STARD7 deficiency in MDA‐MB231 cells (Figure 3D). As a result, the methylation potential of both cell lines was increased upon STARD7 deficiency (Figure 3D). In contrast, steady‐state levels of both creatine and phosphocreatine were significantly reduced upon STARD7 deficiency (Figure 3B). Therefore, a metabolic reprogramming occurs in breast cancer cells lacking STARD7.

As STARD7 deficiency causes the accumulation of SAMs in breast cancer cells and given its role in epigenetic modifications through both histone and DNA methylations, we next conducted ChIP sequencing experiments using the anti‐H3K27Me3 antibody to establish the methylome of both control and STARD7‐depleted MCF7 cells. Histone H3 trimethylation on lysine 27 is associated with transcriptional repression and is catalyzed by the methyltransferases EZH1 and EZH2.^[^ ^27^, ^28^ ^]^ The frequency of H3K27Me3 events increased in cells lacking STARD7 (Figure 3E). This epigenetic modifications was found in promoters as well as in the first or other exons and introns and in 3′ and 5′ UTRs and this distribution did not change upon STARD7 deficiency (Figure 3F). Interestingly, H3K27Me3 events were enriched on specific gene candidates linked to cell proliferation, cell cycle, and oxidative stress (Figure 3G,H). In this context, the H3K27Me3 epigenetic mark was found elevated on the promoter of both *NUF2*, a member of the kinetochore complex^[^ ^29^ ^]^ and Thioredoxin‐interacting protein (*TXNIP*), an Thioredoxin inhibitor^[^ ^30^ ^]^ (Figure 3G,H). As a result, both NUF2 and TXNIP protein levels were downregulated in breast cancer cells lacking STARD7 (Figure 3G,H). Moreover, levels of Thioredoxin reductase (TRXR1), which plays a key role in protection against oxidant injury,^[^ ^31^ ^]^ were increased in breast cancer cells lacking STARD7 (Figure 3H). Therefore, the accumulation of SAMs seen upon STARD7 deficiency has dramatic consequences on the transcription of specific candidates involved in cell proliferation and in the response to oxidative stress.

### 2.4 Loss of STARD7 Leads to Cell Cycle Arrest in Breast Cancer Cells

**Figure 4:**
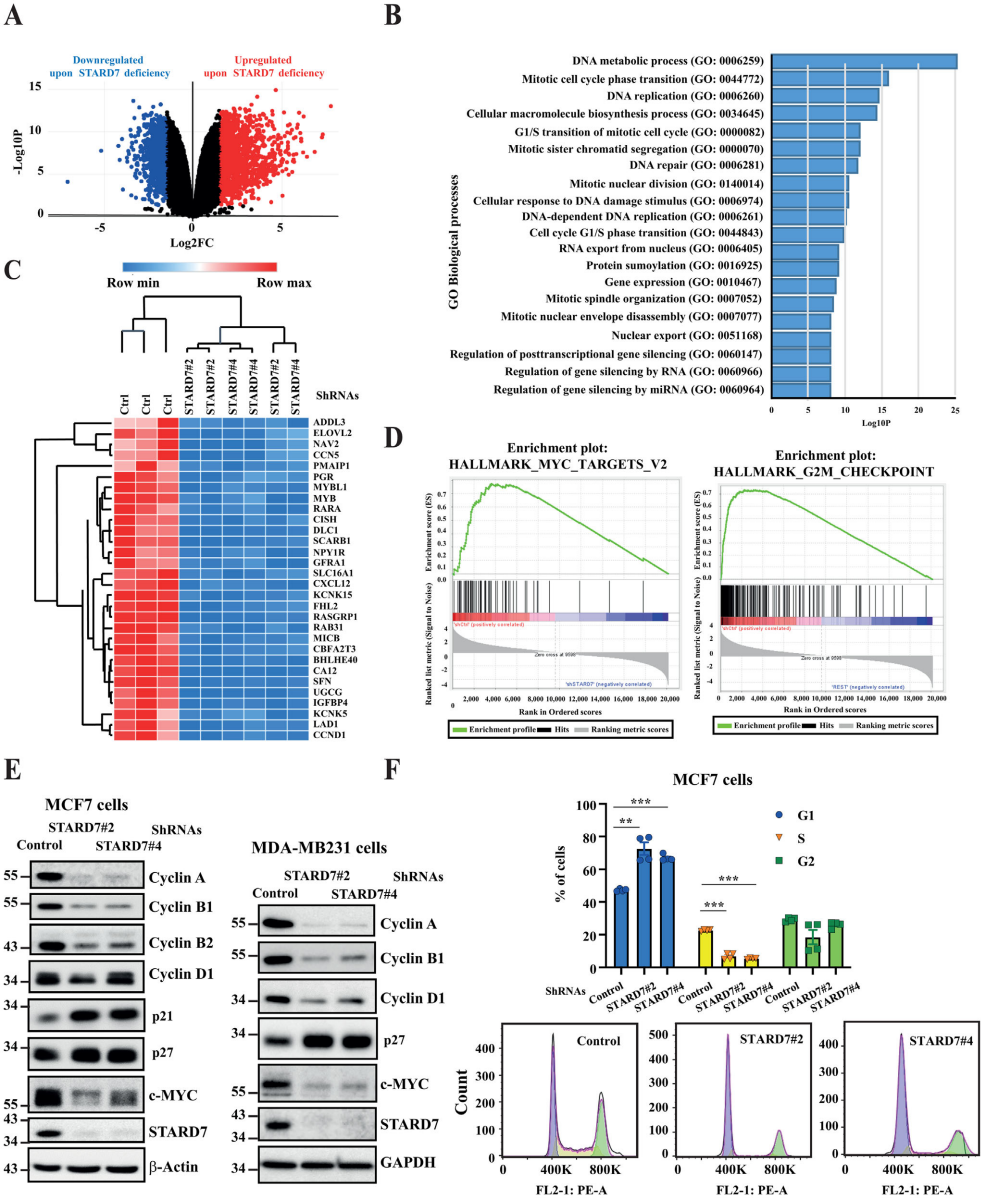
STARD7 deficiency in breast cancer cells leads to defects in cell cycle progression. A) Deregulated transcriptional signature in breast cancer cells lacking STARD7. A volcano plot illustrating the up and downregulated candidates (blue and red dots, respectively) upon STARD7 deficiency in MCF7 cells (*n* = 3). B) STARD7 deficiency causes cell cycle defects in breast cancer cells. A Gene Ontology for biological processes was carried out with transcriptomic data obtained with control versus STARD7‐depleted MCF7 cells (*n* = 3). C) Top 30 candidates downregulated upon STARD7 deficiency in breast cancer cells. A HeatMap showing the most downregulated candidates in MCF7 cells lacking STARD7 is shown (*n* = 3). D) STARD7 deficiency impairs the expression of candidates involved in G2M checkpoint as well as c‐Myc targets. GSEA were carried out with transcriptomic data obtained with control versus STARD7‐depleted MCF7 cells (*n* = 3). E) Impaired expression of candidates involved in cell cycle progression in breast cancer cells lacking STARD7. Extracts from control and STARD7‐depleted MCF7 or MDA‐MB231 cells were subjected to WB analyses using the indicated antibodies. Experiments were repeated at least three times. Representative blots are shown. F) Cell cycle progression defects upon STARD7 deficiency. The percentage of cells in G1, S, or G2 phases was quantified in both control and STARD7‐depleted MCF7 cells by FACS. Two experiments carried out in duplicates are shown and 10^4^ cells were analyzed for each experimental condition (Student's *T*‐test, **p < 0.01, ***p < 0.001, ****p < 0.0001).

To learn more on gene candidates whose expression is regulated by STARD7, we next established the transcriptomic signature of control versus STARD7‐depleted breast cancer cells by RNA sequencing experiments. STARD7 deficiency had dramatic consequences on the transcriptomic signature as hundreds of candidates were not properly expressed (**Figure** **4**A). Interestingly, many dysregulated candidates were functionally linked to DNA metabolic processes, mitotic cell cycle phase transition and DNA replication (Figure 4B). A HeatMap illustrating the most 30 downregulated genes upon STARD7 deficiency in MCF7 cells included key actors of cell progression such as *Cyclin D1* (*CCND1*), *IGFBP4*, *MYB*, and *MYBL1* (Figure 4C). Gene Set Enrichment Analyses (GSEA) conducted with RNA sequencing data confirmed that STARD7 deficiency was linked to defects in the G2M checkpoint and in the expression of c‐MYC targets, suggesting a role for STARD7 in cell cycle progression (Figure 4D). Real‐Time PCR analyses carried out with total RNAs from both control and STARD7‐depleted MCF7 or T47D cells confirmed that *Polo‐like kinase 1* (*PLK1*), *c‐MYC*, *estrogen receptor 1* (*ESR1*, here after referred to as ERα), *retinoic acid receptor alpha* (*RARA*), *Carbonic Anhydrase 12* (*CA12*), *IGFBP4*, *Cyclin D1*, *Cyclin B1* (*CCNB1*) mRNA levels were decreased (Figure S3, Supporting Information). Consistently, a variety of Cyclins were not properly expressed at the protein level in MCF7 cells lacking STARD7 (Figure 4E). Indeed, STARD7 deficiency impaired the expression of Cyclin A, B1 and B2 in MCF7 cells but also in MDA‐MB231 cells (Figure 4E). On the other hand, cyclin kinase inhibitors such as p21 and p27 levels were enhanced upon STARD7 deficiency (Figure 4E). Likewise, cell cycle progression defects were systematically seen in MCF7, MCF10A or MDA‐MB231 cells lacking STARD7. Indeed, STARD7 deficiency caused the accumulation of cells in G1 phase, as evidenced by flow cytometry analyses (Figure 4F and Figure S4, Supporting Information, respectively). Therefore, STARD7 deficiency triggers a transcriptional reprogramming linked to cell cycle arrest.

### 2.5 STARD7 Deficiency Impairs the Expression of Candidates Involved in DNA Replication

**Figure 5:**
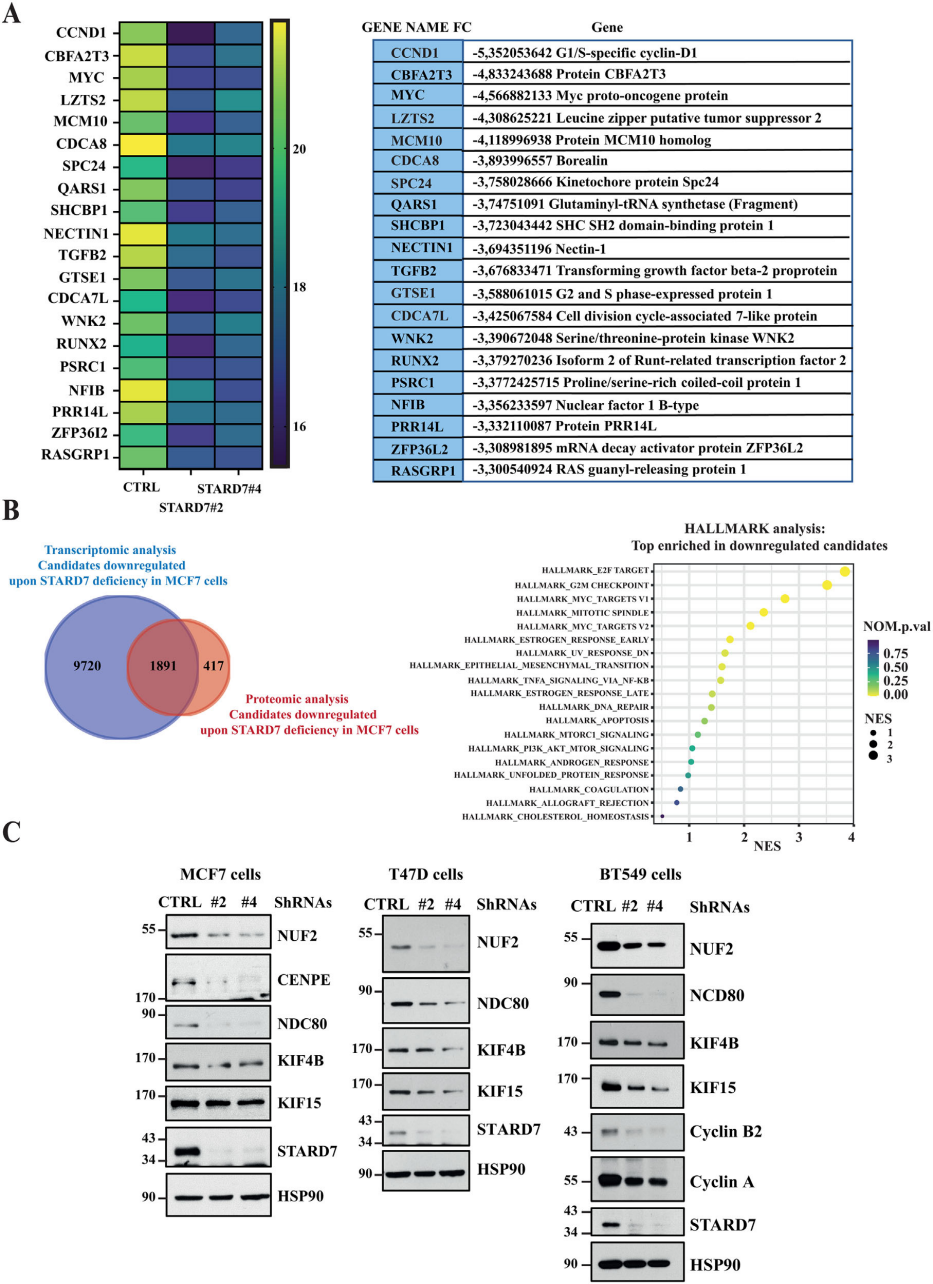
STARD7 promotes the expression of candidates involved in DNA replication in breast cancer cells. A) HeatMap of differentially expressed candidates upon STARD7 deficiency in MCF7 cells from three independent experiments (proteomic analysis). B) STARD7 controls the expression of candidates involved in DNA replication. On the left, data sets of downregulated candidates upon STARD7 deficiency from both transcriptomic and proteomic analyses were compared. On the right, the top 19 significantly downregulated gene sets found in both transcriptomic and proteomic analyses upon STARD7 deficiency in MCF7 cells are shown (HALLMARK analysis) (Mann–Whitney *U* test) (*n* = 3). C) STARD7 promotes the expression of candidates involved in kinetochore formation. Protein extracts from control and STARD7‐depleted MCF7, T47D and BT549 cells were subjected to WB analyses using the indicated antibodies. Representative blots from three independent experiments are shown (MCF7 and T47D cells) and from one additional confirmatory experiment done in BT549 cells.

To further confirm the link between STARD7 and cell cycle progression, we established the proteomic signature of both control and STARD7‐depleted MCF7 cells. A String Interaction Network analyses highlighted a link between STARD7 expression and DNA replication (Figure S5A, Supporting Information). Likewise, key biological processes involving downregulated candidates upon STARD7 deficiency were cell DNA replication and DNA metabolic process (Figure S5B,C, Supporting Information, respectively). In agreement with our Western blot analyses, Cyclin D1 and c‐Myc were identified as downregulated candidates in MCF7 cells lacking STARD7 (**Figure** **5**A). We next compared data sets from both transcriptomic and proteomic signatures seen in MCF7 cells lacking STARD7 and found highly consistent results as 1891 out of 2308 (i.e., 81.93%) candidates downregulated upon STARD7 deficiency at the protein level were also found in the list of downregulated candidates at the mRNA level (Figure 5B, left panel). Consistently, a HALLMARK analysis for top enriched pathways carried out with downregulated candidates found upon STARD7 deficiency at both mRNA and protein levels also highlighted a link with cell cycle (G2M checkpoint, MYC targets, etc.) (Figure 5B, right panel). This conclusion was further supported by the fact that both Cyclin A and B2 levels also decreased upon STARD7 deficiency in TNBC‐derived BT549 cells, as previously shown in MCF7 cells (Figures 5C and 4E, respectively). As an additional evidence linking STARD7 expression to DNA replication in breast cancer cells, levels of candidates involved in kinetochore formation were downregulated in MCF7 or T47D breast cells lacking STARD7. Indeed, levels of NUF2, a core component of the kinetochore outer plate, were decreased upon STARD7 deficiency, most likely due to the enhanced distribution of the H3K27Me3 epigenetic mark on NUF2 promoter (Figures 5C and 3G, respectively). Moreover, NDC80, a NUF2‐associated protein,^[^ ^29^ ^]^ was also less expressed in both MCF7 and T47D cells lacking STARD7 (Figure 5C). Importantly, these conclusions were also relevant in BT549 cells, indicating that our conclusions are relevant in multiple cellular models (Figure 5C). Other members of the kinetochore such as CENPE as well as Kinesins such as KIF15 and KIF4B were also downregulated upon STARD7 deficiency in MCF7, T47D, and BT549 cells (Figure 5C). Therefore, STARD7 supports DNA replication, at least by promoting kinetochore assembly in breast cancer cells.

### 2.6 STARD7 Deficiency Impairs Signaling Pathways Linked to Cell Proliferation in Breast Cancer Cells

**Figure 6:**
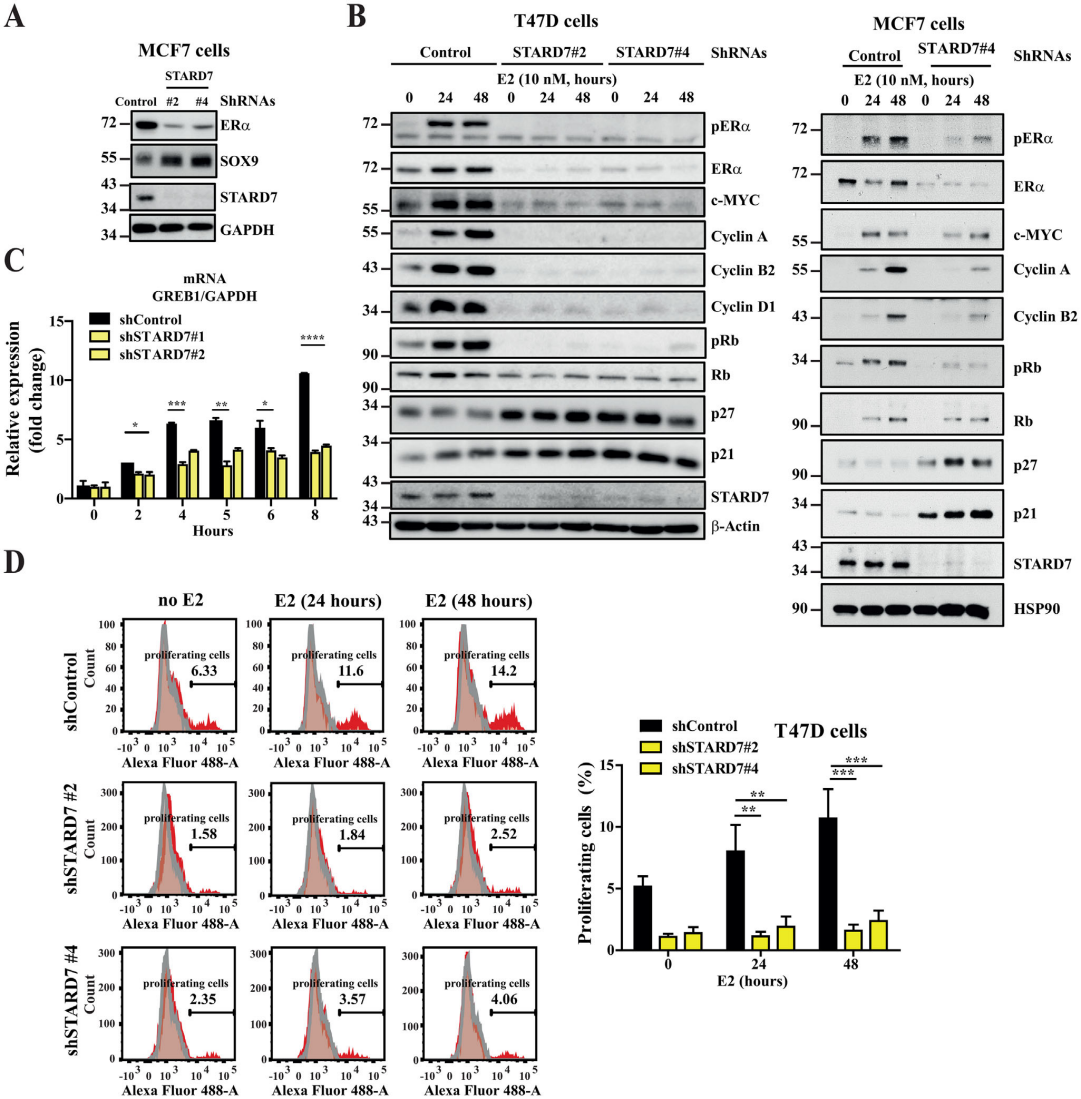
STARD7 deficiency in breast cancer cells impairs ERα signaling. A) Impaired ERα expression upon STARD7 deficiency in breast cancer cells. Extracts from control and STARD7‐depleted MCF7 cells from at least three independent depletions were subjected to WB analyses using the indicated antibodies. Representative blots are shown. B) Impaired estrogens‐dependent signaling upon STARD7 deficiency in breast cancer cells. Control and STARD7‐depleted T47D or MCF7 cells incubated in 5% charcoal‐treated serum were treated or not with estrogens (E2, 10 nM) for the indicated periods of time and the resulting extracts were subjected to WB analyses using the indicated antibodies. Experiments were repeated three times. Representative blots are shown. C) Impaired induction of GREB1 by estrogens in breast cancer cells lacking STARD7. Serum‐starved control and STARD7‐depleted T47D cells were treated or not with E2 (10 nM) for the indicated hours and the resulting extracts were subjected to Real‐Time PCR analyses. mRNA levels of GREB1 in untreated control cells were set to 1 and levels in other experimental conditions were relative to that after normalization with GAPDH levels. Data are from three independent experiments (mean ± SD, two‐way ANOVA, *p < 0.05, **p < 0.01, ***p < 0.001, ****p < 0.0001). D) STARD7 deficiency in breast cancer cells impairs cell proliferation induced by estrogens. Control and STARD7‐depleted T47D cells incubated in 5% charcoal‐treated serum were treated or not with E2 (10 nM) for the indicated hours and the resulting cells were subjected to FACS analyses to quantify the percentage of proliferative cells. Experiments were repeated three times (mean ± SEM, two‐way ANOVA with Dunnettes post‐test **p < 0.01, ***p < 0.001).

Because STARD7 deficiency impairs *ERα* mRNA levels in MCF7 cells, we next assessed whether ERα signaling was defective in these cells. MCF7 cells lacking STARD7 showed lower protein levels of ERα while levels of SOX9, a candidate repressed by ERα signaling in ERα^+^ tumors, were elevated (**Figure** **6**A). Consistently, ERα signaling was strongly defective in STARD7‐depleted T47D or MCF7 cells, as evidenced by a defective estrogen (E2)‐dependent ERα phosphorylation, at least due to decreased ERα protein levels (Figure 6B, left and right panels, respectively). As a result, the estrogens‐dependent induction of c‐MYC, Cyclins A, B2, and D1 was strongly impaired in T47D cells lacking STARD7 (Figure 6B, left panels). Here again, T47D cells lacking STARD7 showed elevated levels of both p21 and p27 (Figure 6B, left panels). This defective E2‐dependent induction of both Cyclin A and Cyclin B2 was also observed in MCF7 cells lacking STARD7 (Figure 6B, right panels). Moreover, both p27 and p21 were further induced upon E2 treatment in MCF7 cells lacking STARD7 (Figure 6B, left panels). However and in contrast to T47D cells, E2‐dependent c‐Myc induction was weakly delayed upon STARD7 deficiency (Figure 6B, right panels). Therefore, STARD7 promotes ERα signaling in breast cancer cells. Consistently, mRNA levels of *growth regulation by estrogen in breast cancer 1* (*GREB1*), an early‐response gene in the estrogen receptor‐regulated pathway that promotes hormone‐dependent cell proliferation,^[^ ^32^ ^]^ were not properly induced by estrogens in T47D cells lacking STARD7 (Figure 6C). Likewise, estrogens fails to trigger cell cycle progression in T47D cells lacking STARD7, as evidenced by flow cytometry analyses (Figure 6D). Therefore, STARD7 is involved in ERα signaling, at least by promoting ERα expression.

**Figure 7:**
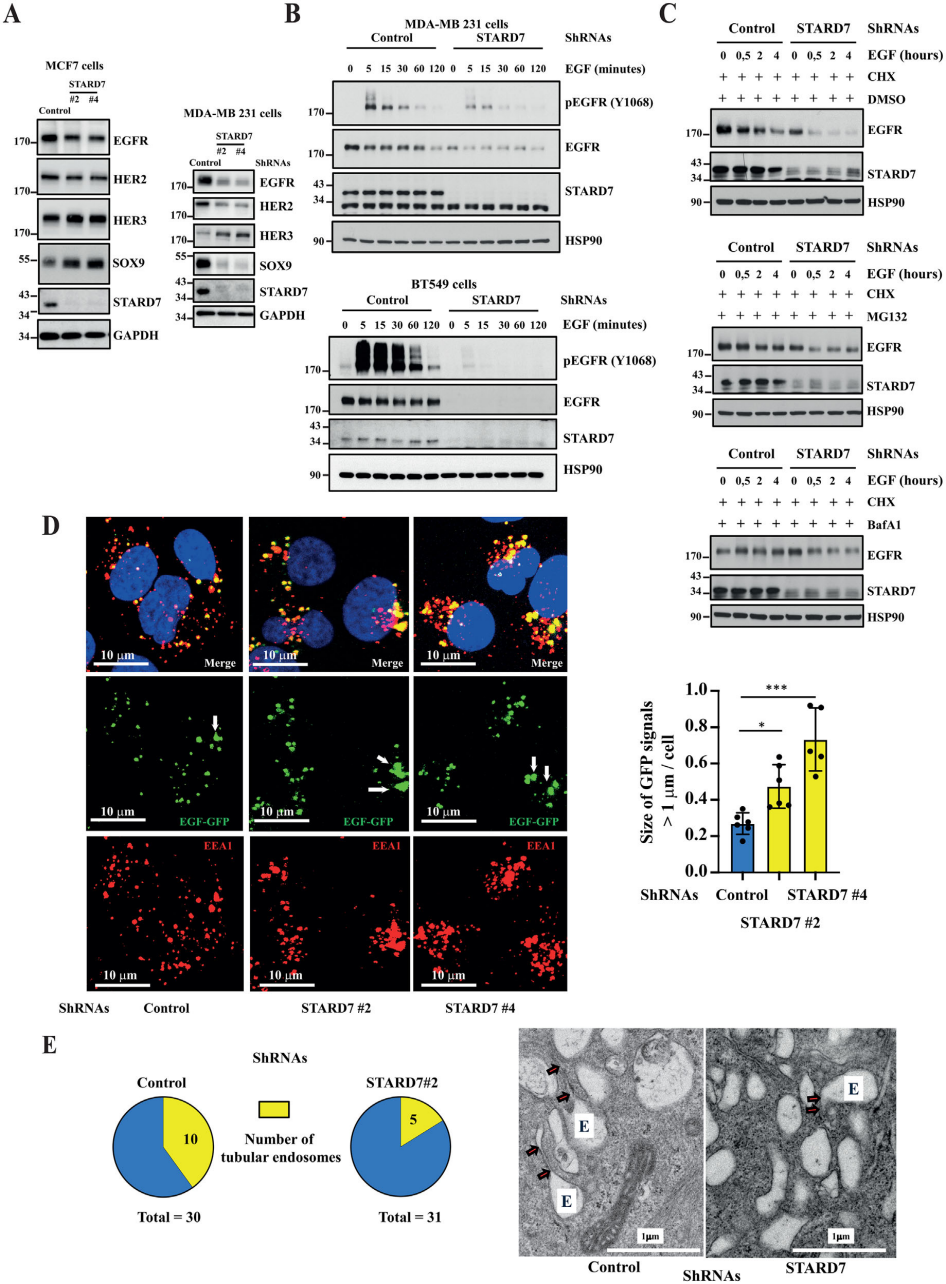
STARD7 deficiency impairs EGFR signaling. A) STARD7 deficiency impairs EGFR expression in TNBC‐ but not in ER^+^‐derived breast cancer cells. Control and STARD7‐depleted MCF7 or MDA‐MB231 cells were subjected to WB analyses using the indicated antibodies (left and right panels, respectively). Experiments were repeated at least three times. Representative blots are shown. B) EGFR signaling relies on STARD7 in TNBC‐derived breast cancer cells. Serum‐starved control and STARD7‐depleted MDA‐MB231 or BT549 cells were treated or not with EGF (50 ng ml^−1^) for the indicated periods of time and the resulting extracts were subjected to WB analyses using the indicated antibodies (top and bottom panels, respectively). Cell extracts were collected 12 days post‐infection. Experiments were repeated at least twice on each cell line. Representative blots are shown. C) STARD7 deficiency enhances EGFR degradation through lysosomes. Control or STARD7‐depleted MDA‐MB231 cells were pretreated with Cycloheximide (CHX, 50 µg ml^−1^) and with DMSO (vehicle), MG132 (25 µM) or Bafilomycin A1 (BafA1) (0.1 µM) for 2 hours and subsequently subjected or not to EGF for the indicated periods of time (top, middle, and bottom panels, respectively). The resulting cell extracts from two independent experiments were subjected to WB analyses. Representative blots are shown. D) Enlarged early endosomes in breast cancer cells lacking STARD7. Control or STARD7‐deficient MDA‐MB231 cells were serum starved and subsequently untreated or stimulated with EGF for 30 minutes and subjected to immunofluorescence analyses. EGF‐bound to EGFR (green) as well as EEA1^+^ endosomes (red) were detected. Nuclei were visualized by DAPI stainings. On the right, a quantification of enlarged (> 1 µm) EEA1^+^ endosomes per cell in EGF‐stimulated control and STARD7‐depleted cells is illustrated. 6 fields for each experimental condition were analyzed (314, 325, and 232 cells for shRNAs Control, STARD7#2 and STARD7#4, respectively) (one‐way ANOVA, ***p < 0.001; *p < 0.05). E) STARD7 deficiency decreases the number of endosomes with tubular structures. Electron micrographs showing endosomes (E) with tubular structures (arrows) in control versus STARD7‐depleted MDA‐MB231 cells are shown. Around thirty photos were randomly taken in both control and STARD7‐depleted cells at the level of endosomes at a magnification of 10 000.

As ERα^+^ cells lacking STARD7 showed cell proliferation defects, we also looked at levels of growth factor receptors and found that STARD7 was dispensable for both HER2 and HER3 expression in MCF7 cells (**Figure** **7**A, top panels). Moreover, STARD7 deficiency modestly impaired EGFR levels (Figure 7A, left panels). TNBC‐derived MDA‐MB231 cells also showed defects upon STARD7 deficiency but molecular mechanisms where different when compared to MCF7 cells as both EGFR and HER2 but not HER3 were not properly expressed in these TNBC‐derived cells (Figure 7A, right panels). Moreover and in contrast to MCF7 cells lacking STARD7, MDA‐MB231 cells showed lower levels of SOX9 upon STARD7 deficiency (Figure 7A, bottom panels). Consistently, EGF‐dependent EGFR phosphorylation on Tyrosine 1068 was strongly impaired in both MDA‐MB231 and BT549 cells lacking STARD7 (Figure 7B, top and bottom panels, respectively). Therefore, STARD7 is essential for EGFR signaling in TNBC‐derived cells, at least by promoting EGFR expression. To better understand why EGFR protein levels decreases in TNBC cells lacking STARD7, we assessed EGF‐dependent EGFR degradation in these cells. To prevent EGFR neosynthesis, we first pretreated control and STARD7‐depleted cells with Cycloheximide, a translation inhibitor and subsequently followed EGFR levels upon EGF stimulation. As expected, EGFR protein levels decreased upon EGF stimulation both in control and STARD7‐depleted cells (Figure 7C, top panels). Moreover, the proteasome inhibitor MG132 did not restore EGFR levels in cells lacking STARD7, suggesting that EGF‐dependent EGFR degradation does not rely on the proteasome in these cells (Figure 7C, middle panels). As expected, the lysosome inhibitor BaFA1 blocked EGF‐dependent EGFR degradation in control cells (Figure 7C, bottom panels). Importantly, BafA1 also restored EGFR levels in cell lacking STARD7 and also slowed down EGF‐dependent EGFR degradation when compared to STARD7‐depleted cells treated with EGF and DMSO (Figure 7C, bottom panels). Collectively, these results suggest that TNBC cells lacking STARD7 show enhanced Lysosome‐dependent EGFR degradation. To further support this conclusion, we also assessed EGF‐bound EGFR trafficking in control versus STARD7‐depleted MDA‐MB231 cells. EGF‐bound EGFR was found in EEA1^+^ endosomes 30 min post‐EGF stimulation in both control and STARD7‐depleted MDA‐MB231 cells (Figure 7D). Importantly, we found an increase of enlarged EEA1^+^ endosomes in cells lacking STARD7 (Figure 7D). Moreover, electronic microscopy analyses indicated that cells lacking STARD7 also showed less endosomes with tubular structures known to be involved in membrane receptor recycling (Figure 7E). Indeed, while 10 out of 30 control cells showed at least one endosome with tubular structures, only 5 out of 31 cells lacking STARD7 did (Figure 7E). Collectively, these results indicate that STARD7 influences the size of early endosomes and promotes membrane receptor recycling.

### 2.7 STARD7 Deficiency Leads to Cell Cycle Arrest Through Transcriptional and Metabolic Reprogramming

**Figure 8:**
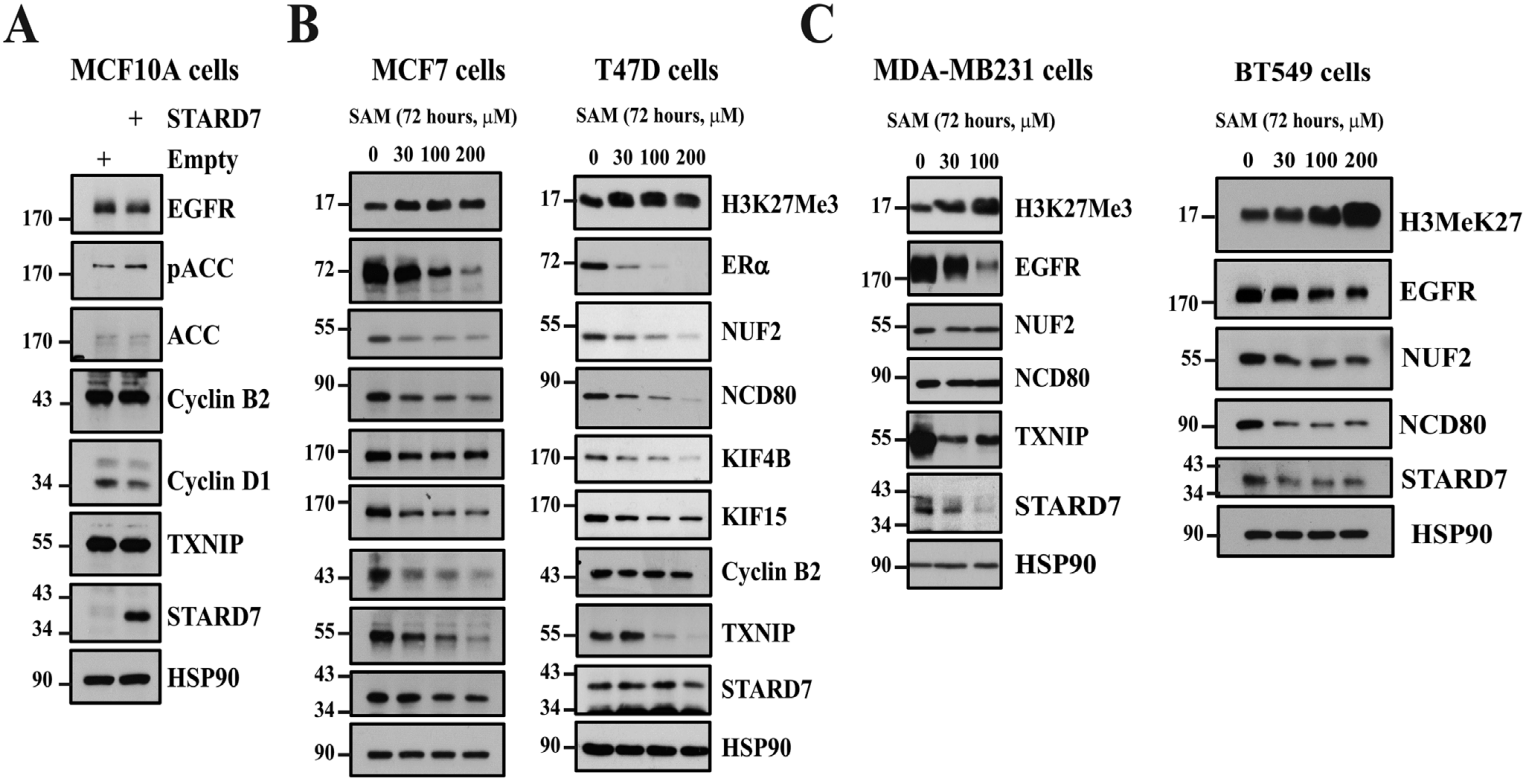
S‐Adenosylmethionine triggers molecular changes seen in breast cancer cells lacking STARD7. A. STARD7 overexpression does not change levels of candidates involved in cell proliferation. Extracts from MCF10A cells overexpressing an empty vector (negative control) or STARD7 were subjected to WB analyses using the indicated antibodies (*n* = 2). B,C) SAM stimulation decreased protein levels of proteins involved in cell proliferation. MCF7 (B), T47D (B), MDA‐MB231, or BT549 (C) breast cancer cells were treated or not with SAM for 72 hours at the indicated concentrations and the resulting extracts were subjected to WB analyses using the indicated antibodies. Experiments were repeated twice for each cell line (MCF7, T47D, and MDA‐MB231 cells). Results were confirmed once in BT549 cells. Representative blots are shown.

While STARD7 deficiency led to multiple molecular defects, it was still unclear what the causal event of this phenotype was. Of note, STARD7 overexpression alone in MCF10A cells did not influence EGFR, ACC, Cyclin B2, Cyclin D1, nor TXNIP protein levels, indicating that STARD7 overexpression alone does not enhance the proliferation of immortalized breast epithelial cells (**Figure** **8**A). In this context and to better link‐enhanced SAM levels to transcriptional reprogramming seen in breast cancer cells lacking STARD7, we treated MCF7, T47D, MDA‐MB231, or BT549 cells with SAM, which impairs breast cancer proliferation.^[^ ^33^ ^]^ SAM enhanced H3K27Me3 levels in all tested cell lines (Figure 8B,C). Moreover, both MCF7 and T47D cells showed lower ERα protein levels upon SAM stimulation and this was also true for EGFR levels in SAM‐treated MD‐MB231 and BT549 cells (Figure 8B,C). TNXIP expression was also strongly impaired upon SAM stimulation in all tested breast cancer cell lines, as were Cyclin B2 levels (Figure 8B,C). Of note, TXNIP expression was not detected in BT549 cells. Finally, SAM also impaired KIF4B and KIF15 expression in both MCF7 and T47D cell lines as well as NUF2 and NCD80 expression in ERα^+^ and BT549 cells but not in MDA‐MB231 cells, which reflects some cell type specificity (Figure 8B,C). It was also interesting to note that SAM also decreased STARD7 protein levels in MCF7, MDA‐MB231, and BT549 cells and only at higher concentrations in T47D cells. Therefore, the stimulation of breast cancer cells with SAM remarkably mimics molecular changes seen upon STARD7 deficiency, which suggests that STARD7, as a lipid transfer protein, promotes cell cycle progression mainly by limiting SAM levels in breast cancer cells.

### 2.8 STARD7 Deficiency Potentiates a Ciliogenesis Signature in Breast Cancer Cells

**Figure 9:**
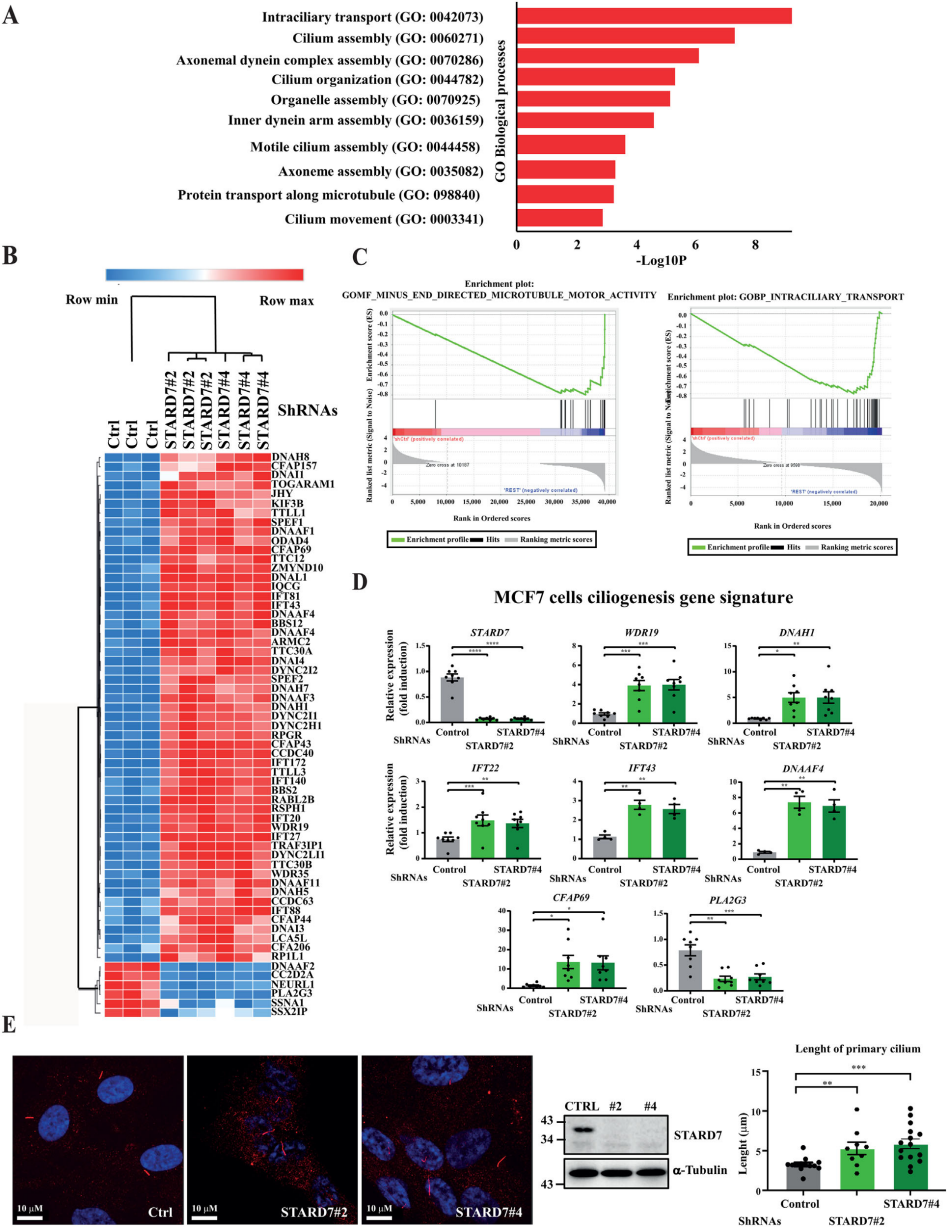
STARD7 deficiency potentiates ciliogenesis. A) A ciliogenesis signature is found in breast cancer cells lacking STARD7. A Gene Ontology for biological processes was carried out with RNA Sequencing data from control and STARD7‐depleted MCF7 cells (three independent depletions). B) Identification of a ciliogenesis signature upon STARD7 deficiency in breast cancer cells. A HeatMap generated with data from the RNA Sequencing experiments is shown. The most differentially expressed candidates between control and STARD7‐depleted MCF7 cells are illustrated (*n* = 3). C) STARD7 deficiency is linked to ciliogenesis. Gene Set Enrichment analyses (GSEA) were carried out with data from RNA sequencing experiments (*n* = 3). D) Enhanced mRNA levels of ciliogenesis genes in breast cancer cells lacking STARD7. Real‐Time PCR analyses were carried out with total RNAs from control and STARD7‐depleted T47D cells. mRNA levels of the indicated candidates were set to 1 in control cells and levels in other experimental conditions were relative to that after normalization with GAPDH levels (mean ± SEM, one‐way ANOVA with Dennett's post‐test, *p < 0.05, **p < 0.01, ***p <0.001, ****p < 0.0001, *n* = 8 distinct experiments). E) Enhanced ciliogenesis upon STARD7 deficiency. Immunofluorescence analyses were carried out with control and STARD7‐depleted hTERT‐RPE1 cells using the anti‐Arl13b to visualize the primary cilium. The length of the primary cilium in control and STARD7‐depleted cells was quantified (histogram on the right) (mean ± SEM, unpaired *T*‐test, **p < 0.01) (see methods for details). WB analyses with extracts from all experimental conditions are also illustrated.

To learn more on biological processes regulated by STARD7, we next concentrated on candidates upregulated in MCF7 cells lacking STARD7. A Gene Ontology for biological processes was done using our RNA sequencing data from control and STARD7‐depleted MCF7 cells and defined intraciliary transport, cilium assembly axonemal dynein complex assembly and cilium organization as the top processes potentiated upon STARD7 deficiency (**Figure** **9**A). Likewise, a HeatMap generated with these results also highlighted a ciliogenesis signature in MCF7 cells lacking STARD7 (Figure 9B). Likewise, Gene Set Enrichment analyses (GSEA) carried out with our dataset also revealed a strong link between the loss of STARD7 and intraciliary transport in MCF7 cells (Figure 9C). This finding appeared relevant to us given the fact that the primary cilium is assembled in the G0/G1 phase in which breast cancer cells accumulate upon STARD7 deficiency.^[^ ^34^ ^]^ Real‐Time PCR analyses indeed confirmed that *WDR19*, *DNAH1*, *DNAAF4*, *IFT22/43*, and *CFAP69* were more strongly expressed at the mRNA level in STARD7‐depleted MCF7, T47D, or MDA‐MB231 cells, while mRNA levels of *PLA2G3*, a negative regulator of ciliogenesis were down (Figure 9D and Figure S6, Supporting Information). Likewise, STARD7 deficiency in the human retinal pigment epithelial RPE‐1 cell line immortalized with hTERT (« hTERT‐RPE1 » cells), an experimental model to assess ciliogenesis, enhances the length of the primary cilium, as evidenced by anti‐Arl13b immunofluorescent analyses (Figure 9E). Therefore, STARD7 is a negative regulator of ciliogenesis.

### 2.9 Loss of STARD7 Leads to Autophagy

**Figure 10:**
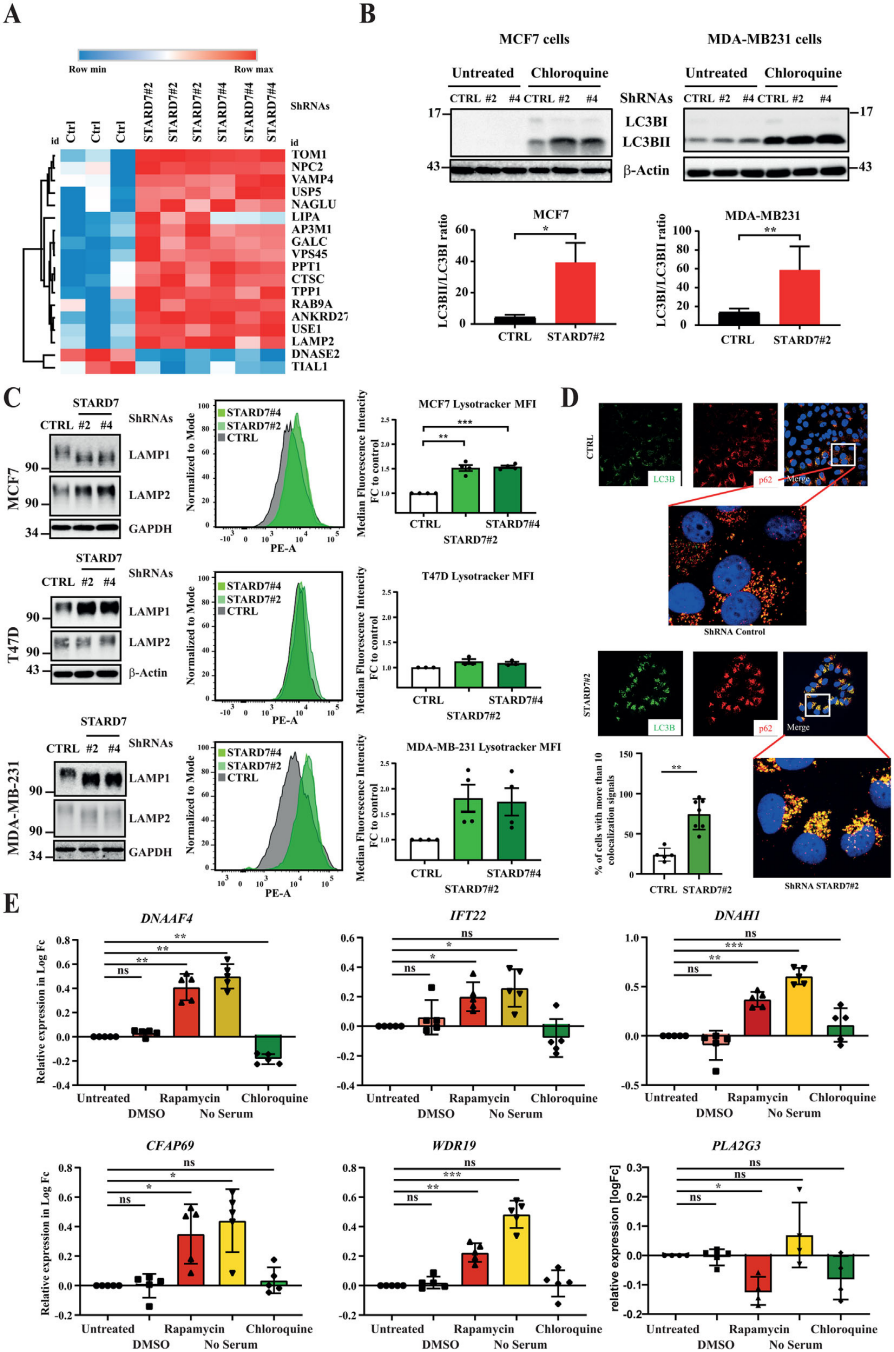
STARD7 deficiency leads to autophagy. A) Enrichment of lysosomal proteins in breast cancer cells lacking STARD7. A Heatmap of proteins involved in GO: Lysosome 0005764 was generated with data from control and STARD7‐depleted MCF7 cells. Data was obtained from proteomics analysis, hierarchical clustering on raw was performed using one minus Pearson's correlation with average linkage method. Relative coloring scheme was applied. B) Enhanced levels of the conjugated form of LC3B in breast cancer cells lacking STARD7. On the top, extracts from control and STARD7‐depleted MCF7 or MDA‐MB231 cells treated or not with Chloroquine (25 µM, 24 hours) were subjected to Western blot analyses using the indicated antibodies. Experiments were repeated at least three times. Representative blots are shown. At the bottom, histograms showing the LCB3II/LCB3I (unconjugated and conjugated forms of LC3B, respectively) ratio after Chloroquine (25 µM) overnight treatment in MCF7 cells. Ratios were calculated for at least three independent experiments (mean ± SEM, paired *T*‐test, *p < 0.05, **p < 0.01). C) Enhanced autophagy in breast cancer cells lacking STARD7. On the left, extracts from control and STARD7‐depleted MCF7, T47D, and MDA‐MB231 cells were subjected to WB analyses using the indicated antibodies. Experiments were repeated at least three times. Representative blots are shown. In the middle, FACS analyses using the lysotracker dye to monitor autophagy in control and STARD7‐depleted breast cancer cells. On the right, histograms illustrating the quantification of FACS data using the lysotracker. The median fluorescence intensity was set to 1 in control cells and values in other experimental conditions were relative to that. Data are from at least three independent experiments per cell line (mean ± SEM, one‐way ANOVA with Dennett's post‐test, *p < 0.05, **p < 0.01, ***p < 0.001). D) STARD7 deficiency causes autophagy in breast cancer cells. Representative immunofluorescence analyses of control and STARD7‐depleted MCF7 cells (shRNA Control and shRNA STARD7#2) after an overnight treatment with Chloroquine (15 µM). Anti‐p62 (red) and LC3B (green) stainings were carried out. DAPI was used for nuclei stainings. The bar graph shows a quantitative analysis of cells undergoing autophagy. The quantification shows the percentage of cells with more than 10 co‐localized points to the total number of analyzed cells (Student's *T*‐test, **p < 0.01). E) The ciliogenesis signature of breast cancer cells lacking STARD7 results from autophagy. MCF7 cells were treated or not with DMSO (vehicle), Rapamycin (20 nM), Chloroquine (25 µM) or were serum‐starved and mRNA levels of the indicated candidates were quantified by Real‐Time PCR analyses. Data are from 5 independent experiments (mean ± EM, one‐way ANOVA with Dennett's post‐test, *p < 0.05, **p < 0.01, ***p < 0.001).

Because autophagy promotes primary ciliogenesis, we next explored whether breast cancer cells lacking STARD7 undergo autophagy.^[^ ^35^ ^]^ We first noticed that multiple lysosomal candidates such as *Niemann‐Pick C2 *protein (NPC2), N‐acetyl‐alpha‐glucosaminidase (NAGLU) and Lysosomal‐associated membrane protein 1 (LAMP1) were enriched at the mRNA level in MCF7 cells lacking STARD7 (**Figure** **10**A). Likewise, MCF7 and MDA‐MB231 cells lacking STARD7 and treated with Chloroquine, which inhibits autophagy by blocking autophagosome fusion with lysosomes, showed elevated levels of the conjugated form of LC3B (LC3BII), a sign of autophagy (Figure 10B, top panels). Breast cancer cells lacking STARD7 also showed elevated levels of LAMP1 or LAMP2, which, together with flow cytometry analyses using Lysotracker, demonstrates that STARD7 deficiency in both MCF7 and MDA‐MB231 but not in T47D cells led to an increased in lysosomal biomass, a hallmark of autophagy (Figure 10C). Likewise, STARD7‐depleted MCF7 cells showed a more pronounced colocalization of LC3B with p62 or with LAMP1, as judged by immunofluorescence analyses (Figure 10D and Figure S7, Supporting Information, respectively).

To prove that the ciliogenesis signature seen upon STARD7 deficiency in breast cancer cells results from autophagy, we next quantified mRNA levels of candidates involved in ciliogenesis in MCF7 cells treated with the mTORC1 inhibitor Rapamycin. In circumstances in which autophagy is triggered by Rapamycin, mRNA levels of multiple candidates dramatically increased (Figure 10E). We also treated MCF7 cells with Chloroquine which strongly decreased *DNAAF4* and *IFT22* mRNA levels and did not significantly change *DNAH1*, *CFAF69*, and *WDR19* mRNA levels in control MCF7 cells. Interestingly, levels of *PLA2G3*, a negative regulator of ciliogenesis,^[^ ^36^ ^]^ were downregulated upon Rapamycin treatment, suggesting that this candidate is regulated by mTORC1 (Figure 10E). To further make the link between autophagy and ciliogenesis, we next hypothesized that expression levels of candidates linked to ciliogenesis in STARD7‐depleted MCF7 cells would decrease upon Chloroquine treatment. In agreement with this hypothesis, we first confirmed that mRNA levels of *DNAAF4*, *IFT22*, *DNAH1*, *CFAP69*, and *WDR19* were increased in STARD7‐depleted versus control MCF7 cells (Figure S8, Supporting Information). Importantly, Chloroquine decreased mRNA levels of these candidates in a dose‐dependent manner in MCF7 cells lacking STARD7 (Figure S8, Supporting Information). Collectively, these data indicate that ciliogenesis occurs in breast cancer cells undergoing autophagy, a process seen upon STARD7 deficiency.

## 3 Discussion

We provide here insights into molecular mechanisms through which metabolic reprogramming influences gene transcription with important consequences on cell cycle. Indeed, the loss of the lipid transfer protein STARD7 leads to a metabolic reprogramming characterized by SAM accumulation, which causes a transcriptional repression, at least due to the accumulation of H3K27me3 on genes involved in cell proliferation. As a result, breast cancer cells lacking STARD7 undergo cell cycle arrest and do not properly signal through both ERα and EGFR. They also show signs of autophagy, which reinforces the ciliogenesis‐associated transcriptional program. Therefore, our data demonstrate that breast cancer cells of any subtype (ERα^+^ tumors and TNBCs) overexpress STARD7 to support cell proliferation.

Breast cancer cells lacking STARD7 show a profound modification of mitochondrial ultrastructure. Indeed, their mitochondria are all in the orthodox state showing a more expanded matrix in which FA oxidation occurs. This observation fits with the fact that STARD7‐depleted cells accumulate carnitine derivatives, which is indicative of increased FA oxidation. Accumulation of carnitine derivatives may reflect overwhelmed mitochondria, a process ultimately leading to lipotoxicity. Molecular mechanisms underlying enhanced FA oxidation upon STARD7 deficiency is currently unclear. Carnitine palmitoyltransferases, which shuttle acylcarnitines between mitochondrial membranes, are properly expressed in breast cancer cells lacking STARD7 (data not shown). It is worth mentioning that levels of ACC, which produces malonyl‐CoA, an inhibitor of FA oxidation,^[^ ^37^ ^]^ are downregulated in breast cancer cells lacking STARD7. Therefore, this finding may explain why STARD7 deficiency is linked to enhanced FA oxidation.

Accumulation of SAMs is an important feature of STARD7‐depleted breast cancer cells. Indeed, SAM treatment remarkably mimics most molecular changes seen upon STARD7 deficiency. Our data demonstrate that enhanced SAM levels impact on gene transcription through the deposition of the H3K27me3 epigenetic mark on candidates involved in cell cycle progression. Yet, some candidates downregulated upon STARD7 deficiency do not show differences of histone methylation on their promoter. Indeed, our transcriptomic and ChiP sequencing data do not significantly overlap, suggesting that additional mechanisms underlie the STARD7's capacity to promote cell cycle progression, including other repressive histone modifications not assessed in this study. Additional mechanisms may include UPR signaling, as multiple UPR effectors such as PERK, BIP, and XBP1 are more expressed in TNBC‐derived cells lacking STARD7, which could, therefore, explain why these cells undergo cell cycle arrest. Importantly, we found that EGFR was massively downregulated in TNBC‐derived cells lacking STARD7, a phenomenon also seen upon SAM stimulation of MDA‐MB231 cells. Cells lacking STARD7 actually show less endosomes with tubular structures, which, combined with decreased Rab11 levels, suggests that EGFR recycling is impaired. Consistently, EGFR degradation through lysosomes is potentiated upon STARD7 deficiency, indicating that the loss of a lipid transfer protein has dramatic consequences on the trafficking of membrane receptors, at least through deregulated contacts between several intracellular organelles. ERα is another candidate not found in our ChIP sequencing data, despite a massively downregulated expression upon STARD7 deficiency in MCF7 cells but the underlying molecular mechanisms remain unclear. ERα levels are nevertheless decreased upon SAM stimulation in both MCF7 and T47D cells, which therefore indicate that SAM may trigger the transcriptional repression of any candidate involved in ERα expression.

It is currently unknown why breast cancer cells lacking STARD7 accumulate SAMs. One possibility could be linked to the capacity of STARD7 to transport PC to mitochondria. Breast cancer cells lacking STARD7 do not have enough PC in mitochondria and may therefore try to synthetize PC from Phosphatidylethanolamine (PE) through a cascade of reactions relying on Phosphatidylethanolamine N‐Methyl Transferase (PEMT) and on SAM (Figure S9, Supporting Information). PEMT enzymatic activity is dependent on the availability of both the substrate PE and on SAM.^[^ ^38^ ^]^ Therefore, breast cancer cell lacking STARD7 may increase SAM levels to stimulate PEMT enzymatic activity. Another reason may come from the role of STARD7 in Coenzyme Q synthesis.^[^ ^9^ ^]^ Coenzyme synthesis, a multi‐step process, requires SAM as a co‐factor for both *O*‐ and *C*2‐methylations.^[^ ^39^ ^]^ Therefore, SAMs accumulation may be an indirect consequence of a defective Coenzyme Q synthesis seen in breast cancer lacking STARD7. Interestingly, we also noticed that STARD7 deficiency downregulated creatine levels, possibly because of a defective synthesis. If so, it is important to mention that creatine synthesis also relies on the methyl donor SAM, similarly to Coenzyme synthesis.^[^ ^40^ ^]^ Here again, a defective creatine synthesis may contribute to elevated SAM levels seen in breast cancer cells lacking STARD7.

It was first demonstrated that STARD7 deficiency causes defects in PC transport to mitochondria. It is, therefore, not surprising to see that STARD7 deficiency deregulates multiple metabolic pathways occurring in this organelle. The enhanced mitochondria‐ER contact sites seen in breast cancer cells lacking STARD7 may be a compensatory mechanism to support lipid transfer. Indeed, beside the active lipid transport by STARD7, other ways to transfer lipids exist, such as diffusion, membrane merge or passive non vesicular exchange. Those processes are only possible in very close contact sites between membranes such as in MAMs. Therefore, in the situation of a limited active transport seen upon STARD7 deficiency, it is expected for resulting cells to increase their direct membrane contact sites for passive transport of lipids. Yet, underlying molecular mechanisms involved in the increase of MAMs in breast cancer cells lacking STARD7 are currently unknown. It is nevertheless important to mention that mitochondria‐ER contact sites are where autophagosomes are formed and where the VAPB‐PTPIP51 protein complex, known to regulate autophagy, is tethered.^[^ ^41^, ^42^ ^]^ Enhanced MAMs seen in cells lacking STARD7 may therefore contribute to autophagy. MAMs are also essential for the ER‐mitochondria Ca^2+^ transfer but more mitochondria‐ER contact sites seen upon STARD7 deficiency may lead to mitochondrial Ca^2+^ overload, which triggers apoptosis. Interestingly, levels of the voltage‐dependent anion channel VDAC are dramatically decreased in MAMs and this could be seen as a pro‐survival adaptation in breast cancer cells lacking STARD7.

As an evidence linking the phenotype of STARD7‐depleted breast cancer cells with a mitochondrial function of STARD7, interactomic studies carried out with mitochondrial proteins identified an interaction between STARD7 and several candidates, including CHCHD10.^[^ ^43^ ^]^ Interestingly, CHCHD10 protein levels were strongly downregulated in breast cancer cells lacking STARD7, as demonstrated through our proteomic studies, suggesting that STARD7 may bind and stabilize CHCHD10. Therefore, phenotypic alterations found in breast cancer cells lacking STARD7 result from multiple molecular mechanisms, including a defective PC transport and possibly destabilization of important mitochondrial proteins.

In conclusion, our study defines STARD7 as a candidate that promotes cell proliferation as well as both ERα‐ and EGFR‐dependent signaling cascades in breast cancer. It remains to be seen whether STARD7 inhibition would be an elegant pharmacological approach to treat breast malignancies and/or to overcome resistance to targeted therapies through the induction of cell cycle arrest. Given the key role of STARD7 in mitochondrial homeostasis, it is expected that the metabolic reprogramming seen upon STARD7 deficiency may also be relevant in nontransformed breast epithelial cells. In this context, nontumorigenic epithelial MCF10A cells also undergo cell cycle arrest when lacking STARD7. Therefore, some caution should be taken when considering STARD7 as a potential therapeutic target.

## 4 Experimental Section

### Cell Lines, Reagents and Clinical Cases of Breast Cancer

All cell lines used in this study were from ATCC and were routinely checked for Mycoplasma contamination. These cells were characterized by ATCC, using a comprehensive database of short tandem repeat (STR) DNA profiles. Frozen aliquots of freshly cultured cells were generated and experiments were done with resuscitated cells cultured for <6 months. Unless stated otherwise, MCF7, MDA‐MB231, BT549, and LentiX cells were cultured in DMEM high glucose (Lonza) + 10% Fetal Bovine Serum (Gibco), 1% Penicillin/Streptomycin (Lonza). T47D cells were cultured in RPMI (BioWest) + 10% FBS, 1% Penicillin/Streptomycin. BT549 cells were kindly provided by C. Gilles (GIGA Cancer, University of Liege, Belgium). hTERT‐RPE1 retinal cells were cultured in DMEM/F12 (Gibco) supplemented with 10% FBS and 1% Penicillin/Streptomycin. MCF10A cells were cultured in DMEM/F12 (Gibco) medium supplemented with 5% horse serum (Thermo Fisher Scientific), 20 ng ml^−1^ EGF (Sigma), 0.5 mg ml^−1^ hydrocortisone (STEM CELL Technologies), 100 ng ml^−1^ cholera toxin (Sigma), 10 ug ml^−1^ insulin (Sigma) and 1% Penicillin/Streptomycin. All cells were maintained in a 37 °C incubator in 95% humidity and 5% CO_2_. SAM was purchased from NEB (#B9003). Tumors and normal adjacent tissues from clinical cases of breast cancer were obtained at the Biobank of the University Hospital of Liege (CHU, Sart‐Tilman). Clinical parameters of these tumors are described in **Table** **1**. Our study was approved by the ethical committee of the University Hospital of Liege.

**Table 1:** Clinical data breast cancer patients.

Triple negative breast cancers			
Histological analyses			Patient age
Infiltrating ductal carcinoma		1	73
Infiltrating ductal carcinoma		2	84
Infiltrating ductal carcinoma		3	87
Infiltrating ductal carcinoma		4	69
Infiltrating ductal carcinoma		5	78
Infiltrating ductal carcinoma		6	54
Infiltrating ductal carcinoma		7	23
Infiltrating ductal carcinoma		8	62
Infiltrating ductal carcinoma		9	44
Infiltrating ductal carcinoma		10	61
Infiltrating ductal carcinoma		11	89
ER^+^ breast cancer			
Histological analyses			Patient age
Infiltrating ductal carcinoma	ER^+^ PR^+^ HER2 1^+^	1	59
Infiltrating ductal carcinoma	ER^+^ PR^+^ HER2 1^+^	2	83
Infiltrating ductal carcinoma	ER^+^ PR^+^ HER2^‐^	3	72
Infiltrating ductal carcinoma	ER^+^ PR^+^ HER2 1^+^ FISH neg	4	57
Infiltrating ductal carcinoma	ER^+^ PR^+^ HER2 2^+^ FISH neg	5	53
Infiltrating ductal carcinoma	ER^+^ PR^+^ HER2 1^+^ Ki67 15%	6	80

### Lentiviral and Retroviral Vector Production and Cell Transduction

Transfection mixture was prepared in 800 µL OptiMEM (Gibco) by adding following plasmids: 12 µg of psPAX2 (AddGene #12260), 5 µg of pVSV‐G (AddGene #138479) and 12 µg of expression vector carrying the shRNA sequence, in the presence of 80 µL of TransIT‐LT1 Transfection Reagent (Mirus, Mir2360). Expression vectors were: pLKO.1‐puro‐Nontarget shRNA Control (Sigma, SHC002), pLKO.1‐puro‐shSTARD7#2 (Sigma, TRCN000028081) and pLKO.1‐puro‐shSTARD7#4 (Sigma, TRCN0000155648). After 15 minutes of incubation at room temperature, the transfection mixture was added dropwise on top of the 50–60% confluent LentiX cells on T75 cm^3^. Medium was changed within 6–16 hours, with 8 ml of a complete culture medium. After 72 hours of viral particle production, the medium with LV was collected, centrifuged at 800 × *g* for 10 minutes, 0.22 µL filtered and either added directly to cells to be transduced or frozen in –80 °C.

For cell transduction, on day 0 (D0), cells were transduced using the medium containing lentiviral particles in the presence of 8 µg ml^−1^ of Polybrene transfection reagent (Millipore). After 24 hours (D1), the medium was changed to the selection medium with 1 µg ml^−1^ Puromycin (InVivoGen) for 48 hours. Afterwards (D3‐D4), cells were split using the Trypsin/EDTA solution (BioWest) and seeded for experiments. All experiments were done on D5‐D6 post‐transduction.

For retroviral infections of MCF10A cells, the human STARD7 coding sequence was subcloned into pbabe‐puro (Addgene #128041). Virus‐producing Phoenix cells were transfected with a pbabe‐puro empty vector or with the pbabe‐hSTARD7 construct. At 48 hours post‐transfection, the supernatant containing virus was collected, filtered and used for infection of MCF10A cells. At 24 hours postinfection, media were replaced with a medium dedicated to the culture of MCF10A cells (see here before) and complemented with Puromycin. At 72 hours postselection, cells were collected for Western blot analyses.

### Cell Treatments

For estrogens (E2) treatments, control and STARD7‐depleted MCF7 or T47D cells on D1 or D4 (MCF7 and T47D cells, respectively) post‐transduction were first seeded in six well plates in DMEM:F12 without phenol red (Gibco), supplemented with 5% of charcoal/dextran treated FBS (HyClone) and 1% of Penicillin/Streptomycin for 72 or 48 hours (MCF7 and T47D cells, respectively) as a pretreatment to deplete hormones. Note that for MCF7 cells, Puromycin was added in the DMEM:F12 without phenol red (Gibco), supplemented with 5% of charcoal/dextran treated FBS (HyClone) for 48 hours. Afterwards, the medium was changed to a medium with β‐Estradiol (E2) (10 nM) (Sigma–Aldrich, E2257) and cells were harvested at the indicated time points (for protein lysates, 30 minutes, 60 minutes, 24 hours and 48 hours), RNA extraction (2, 4, 5, 6, and 8 hours) and EDU proliferation analysis (24 and 48 hours). For EGF treatments, cells were first serum‐starved overnight or for 24 hours in a medium with 0% FBS before the addition of EGF (Cell Signaling, #9908). Treatments with Chloroquine (25 µM) (Sigma–Aldrich), C6628‐25) and Rapamycin (20 nM) (LC Laboratories, R‐5000) were carried out for 24 hours with cells cultured in DMEM high glucose (Lonza) + 10% Fetal Bovine Serum (Gibco), 1% Penicillin/Streptomycin (Lonza).

### Real‐Time PCR Analyses

Total RNAs were extracted from cultured cells using the column based extraction E.Z.N.A. Total RNA Kit I (Omega Biotek) according to the manufacturer's protocol. cDNAs were obtained using the RevertAid H Minus First Strand cDNA Synthesis Kit using oligo(dT)18 primers and 1 µg of total RNAs as a template. Real‐time PCR reactions were carried out on Light Cycler480 (Roche) with TB Green Premix Ex Taq II Tli RNase H Plus (Takara Bio) and specific primers designed with the PrimerBlast software (NCBI). Primer sequences can be found in **Table** **2**. Ct values were used to calculate fold change of expression using the 2^‐ Δ ΔCt^ method. HSP90B1, GAPDH, and β‐Actin were used as housekeeping controls.

**Table 2:** List of primers.

GAPDH	F: GCATCTTCTTTTGCGTCGC R: CCAAATGCGTTGACTCCGA
HSP90B1	F : CTGTATTCAGGCCCTTCCCG R : ACCACAGCCTTTTCAATCTTGT
ACTB	F: AGAGCTACGAGCTGCCTGAC R: AGCACTGTGTTGGCGTACAG
STARD7	F : ATTCAGAGGGCAAAGAGCAA R : AAGGTGGGTGCCTGTAATTG
ESR1	F : GATCAACTGGGCGAAGAGGG R : CATTTTCCCTGGTTCCTGTCC
CCND1	F : ATCAAGTGTGACCCGGACTG R : CTTGGGGTCCATGTTCTGCT
CCNB1	F : CTGCTGGGTGTAGGTCCTTG R : AGCTGAAGGTTTTGCTTCCTTC
PLK1	F : CCGCAATTACATGAGCGAGC R : GCTTGGTGTGATCCTGGAAGA
IGFBP4	F : GCAGAAGCACTTCGCCAAAA R : CTCTCGAAAGCTGTCAGCCA
RARA	F : CACACACCTGAGCAGCATCAC R : CGGTCCTTTGGTCAAGCAGT
AURKA	F : GTGGGGGATATCTCAGTGGC R : ATGGAGTGAGACCCTCTAGC
PTGS2	F : TGCGCCTTTTCAAGGATGGA R : ACATCATCAGACCAGGCACC
MYBL2	F : CAGCCACTTCCCTAACCGCA R : TGTCCACTGCTTTGTGCCAT
MYC	F: CCGCTTCTCTGAAAGGCTCT R: CTAACGTTGAGGGGCATCGT
CFAP69	F: AACTGTGTGGCTTGCCATTT R : GCTTCTTTGGTGGTTCTGCAT
DNAH1	F: AGATGGCCGTTATCTGGCTC R : CACGCTTTATCCAGTGTCGC
WDR19	F : TCCGCTTGTACATGGCTCTG R : GGCCATCTCGGAGGGAATTT
IFT43	F : ACAGCTGGATCTGAACGCAT R : TCCAGGTCACGGTAGGTCAT
PLA2G3	F : GGCCTAAAACCTCAGGGTGC R : AGCATGTTGGTAACCTCGGG
IFT22	F : TGTGGTGGCGATGCTAAGTT R : TTCAAGGGTGGCGACAAAGA
DNAAF4	F : GGCACATGTACGACGTGGA R : TTCATAATCCTGTAGGCCTTCTAC

### Transcriptomic Analyses by RNA Sequencing

RNA sequencing was performed on libraries prepared from total RNA samples from MCF7 cells (shControl, shSTARD7#2, shSTARD7#4). Three biological replicates were analyzed for each condition. Total RNAs were extracted by using the E.Z.N.A. Total RNA Kit (Omega Bio‐tek) according to the manufacturer's protocol. Total RNAs before elution were treated with TURBO DNase (ThermoFisher Scientific, AM2238). RNA integrity was verified on a Bioanalyser 2100 with RNA 6000 Nano chips (Agilent technologies). RNA integrity number score was above 9 for every sample. Libraries were prepared using Truseq stranded mRNA Sample Preparation Kits (Illumina) following manufacturer's instructions. Libraries were validated using QIAxcel Advanced System and quantified by qPCR using the KAPA library quantification kit. Libraries were multiplexed and sequenced on an Illumina NextSeq500 sequencer to generate more than ≈25 000 000 paired‐end reads (2  ×  150 bases) per library. Raw reads were demultiplexed and adapter‐trimmed using Illumina bcl2fastq conversion software v2.20. Reads were processed within the nf‐core/rnaseq‐1.4.2 pipeline^[^ ^44^ ^]^ using STAR aligner, the human reference genome GRCh38 and the gene annotations from Ensembl release 97. Quality of the sequencing data was successfully controlled using QC modules of the pipeline and a report has been compiled with MultiQC. The data has been deposited to the Genomic Expression Omnibus (GEO) with the following accession number: GSE251873.

### SDS‐PAGE and Western Blot Analyses

Cells were washed twice in ice‐cold PBS and scrapped in an ice‐cold RIPA lysis buffer (50 mM Tris‐HCl pH 7.4, 2 mM EDTA, 50 mM NaF, 150 mM NaCl, 0.1% SDS, 0.5% sodium deoxycholate, 1% NP40) supplemented with cOmplete Protease Inhibitor (Roche) and PhosStop (Roche). Lysates were cleared by centrifugation for 15 minutes, 15 000 × *g* at 4 °C and protein concentrations were measured using the Pierce BCA Protein Assay Kit (Thermo Scientific) according to manufacturer's instructions. Samples were denaturated by boiling for 7 minutes in a Laemmli Buffer with β‐mercaptoethanol and 20 µg of total proteins were loaded per well of SDS‐PAGE gel (8–14%). Samples were separated under reducing conditions and transferred to PVDF membrane (Immobilion‐P, Millipore), using wet transfer chambers. Membranes were blocked in 5% skimmed milk for 45 minutes, cut and incubated with primary antibodies overnight on rotor at 4 °C. A list of primary antibodies is available in **Table** **3**. The following day, membranes were washed five times in TBS‐T and incubated for 1 hour at room temperature with HRP conjugated secondary antibodies donkey antirabbit (GE Healthcare, NA934V) or sheep antimouse (GE Healthcare, NA931V). After subsequent five washing steps, membranes were visualized with the Pierce ECL Western (Thermo Scientific) or SuperSignal West Femto Maximum Sensitivity Substrate (Thermo Scientific) on the ImageQuant LAS 4000 mini (GE Healthcare) or using CL‐XPosure film (Thermo Scientific).

**Table 3:** List of antibodies.

Name of antibody	Reference number	Company
GAPDH (D16H11)	#5174	Cell Signaling Technologies
STARD7	PAS‐30772	Invitrogen
ACTIN‐HRP (AC‐15)	A3854	Sigma
Mitofusin2 (D2D10)	#9482	Cell Signaling Technologies
FIS1	HPA017430	Sigma
DRP1 (C‐5)	sc‐271583	Santa Cruz
Tri‐Methyl‐Histone H3 (Lys27) (C36B11)	#9733	Cell Signaling Technologies
Cyclin A (B‐8)	sc‐271682	Santa Cruz
Cyclin B1	A305‐000A	Bethyl Laboratories Inc. Montgomery, TX, USA
Cyclin B2	A304‐509A	Bethyl Laboratories Inc. Montgomery, TX, USA
Cyclin D1 (92G2)	#2978	Cell Signaling Technologies
Cyclin E (E‐4)	sc‐ 377100	Santa Cruz
P21 Waf/Cip1 (12D1)	#2947	Cell Signaling Technologies
P27 Kip1 (D69C12)	#3686	Cell Signaling Technologies
MYC (D84C12)	#5605	Cell Signaling Technologies
ERα (HC‐20)	sc‐543	Santa Cruz
SOX9	AB5535	Sigma‐Aldrich
EGFR (A‐10)	Sc‐373746	Santa Cruz
Phospho‐ERα (S118)	#16J4	Cell Signaling Technologies
HER2/ErbB2 (29D8)	#2165S	Cell Signaling Technologies
HER3/ErbB3 (D22C5)	#12708S	Cell Signaling Technologies
Phospho‐EGFR (Y1068)	#3777	Cell Signaling Technologies
TrxR1 (B‐2)	sc‐28321	Santa Cruz
TXNIP (D5F3E)	#14715	Cell Signaling Technologies
LAMP1 (D401S)	#15665	Cell Signaling Technologies
LAMP2 (H4B4)	sc18822	Santa Cruz
PERK (C33E10)	#5683	Cell Signaling Technologies
LC3B (D11)	#3868	Cell Signaling Technologies
p62	NBP1‐48320	Novus
Rab11	#3539	Cell Signaling Technologies
Rb	#9309	Cell Signaling Technologies
Phospho‐Rb (s780)	9307S	Cell Signaling Technologies
Phospho‐ACC	#11818	Cell Signaling Technologies
ACC	#3662	Cell Signaling Technologies
E‐cadherin	610182	BD Bioscience
HSP90	sc‐13119	Santa Cruz
Vimentin	Ab92547	Abcam
Atf4	#11815	Cell Signaling Technologies
IP3R	#8568	Cell Signaling Technologies
VDAC	#4661	Cell Signaling Technologies
FACL‐4 (ACSL4)	#38493	Cell Signaling Technologies
COXIV	#4850	Cell Signaling Technologies
Calreticulin	Sc‐11398	Santa Cruz
GRP75	#3593	Cell Signaling Technologies
SERCA2	#9580	Cell Signaling Technologies
Nuf2	# **A304‐318A**	Bethyl Laboratories Inc. Montgomery, TX, USA
CENPE	# **A301‐942A**	Bethyl Laboratories Inc. Montgomery, TX, USA
KIF4B	# **PA5‐68147**	Invitrogen
KIF15	# **A302‐706A**	Bethyl Laboratories Inc. Montgomery, TX, USA
IRE1α (14C10)	# 3294	Cell Signaling Technologies
BIP (C50B12)	# 3177	Cell Signaling Technologies
pIRE1α (Ser724)	# ab124945	Abcam
peIF2α (Ser51)	# 9721	Cell Signaling Technologies
eIF2α	# 9722	Cell Signaling Technologies
XBP1s (D2C1F)	# 12782	Cell Signaling Technologies
PDI	#3501	Cell Signaling Technologies
NCD80 (HEC1)	# 300‐771A	Bethyl Laboratories Inc. Montgomery, TX, USA

Densitometric analyses of Western blot bands were performed using the GeneTools software (Syngene, Cambridge, UK). Bands detection and quantification were carried out automatically by the software, which identifies bands, calculates their intensity and substracts background using the default rolling ball correction. Protein band intensities were normalized to corresponding loading controls (β‐actin, GAPDH or HSP90) and resulting values were expressed relative to control conditions (see our supplementary file).

### Immunofluorescence Analyses

For immunofluorescences with TMRE (Tetramethylrhodamine, ethyl ester) and ER‐Tracker green, control or STARD7‐depleted MCF7 or MDA‐MB231 cells were seeded on Cellview cell culture dishes (35 mm, four compartments) (Greiner bio‐one, Cat#627870) overnight in the growth media. Cells were washed once with PBS then once with HBSS and incubated for 30 minutes at room temperature with 50 nM TMRE (Invitrogen, Cat#T669), 1 µM ER‐Tracker green (Invitrogen, Cat# E34251) and 1 µM Hoechst 33342 (Thermo Scientific, Cat# 62249) in HBSS covered from light. Cells were subsequently washed thrice for 5 minutes at room temperature in PBS. All samples were acquired with a LSM980 Airyscan 2 super‐resolution system (Carl Zeiss, Oberkochen, Germany), in SR mode (pixel size 0.035 µm), equipped with a Plan‐Apochromat 63x/1.4 oil objective. We imaged TMRE (red), ER‐tracker (green), and Hoechst (blue) in 2D, respectively using a 561 nm laser at 1.5%, a longpass filter LP570 and gain at 600 V; a 488 nm laser at 4.0%, a 495–550 nm bandpass filter and gain at 600 V and finally, a 405 nm laser at 4.0%, a shortpass filter SP505 nm and gain at 700 V. We acquired between 3 and 5 random field images, for a total of at least 30 cells per sample. The images were analyzed using the QuPath software, as described.^[^ ^45^ ^]^ All cells were manually annotated and the Pearson's coefficient was calculated for each cell, using the script from MicroscopyRA: (https://gist.github.com/Svidro/68dd668af64ad91b2f76022015dd8a45#file‐colocalization‐of‐channels‐per‐detection‐0‐2‐0‐groovy).

To measure the length of the primary cilium, hTERT‐RPE1 cells were kept in DMEM/F12 medium supplemented with 10% FBS and 1% pen/strep in an incubator with a 5% CO2 and 95% humidified atmosphere. hTERT‐RPE1 cells were transduced with the LV‐sh Control (CTRL), LV‐sh STARD7#2 or LV‐sh STARD7#4 construct and the medium was changed to medium containing 1 µg ml^−1^ of puromycin for 48 hours. After that, cells were split and seeded for experiments. Three days after transduction, cells were seeded on coverslips. Four days after transduction, cells were starved with no serum Optimem for 48 hours. Cells were fixed in 4% PFA on day six after transduction, blocked, permeabilized and stained with a mouse anti‐Arl13b antibody overnight. The next day, coverslips were incubated with a secondary goat antimouse IgG AlexaFluor568 antibody and DAPI and mounted. Samples were visualized using a Zeiss HR LSM 880 confocal microscope. A Z stack of 4 µm was taken for each condition. Data was analyzed using the Imaris software. Six images of cells transduced with the LV‐sh CTRL construct (measurements: *n* = 12) and nine images of cells transduced with the LV‐shRNA STARD7 construct (three images of with cells transduced with the shRNA STARD7#2 construct, measurements: *n* = 10, six images of cells transduced with the shRNA STARD7#4 construct, measurements: *n* = 15) were analyzed. Statistical significance was determined with the unpaired Student's *T*‐test, **p < 0.01.

To assess EGFR trafficking upon EGF stimulation, control or STARD7‐depleted MDA‐MB231 cells were seeded on coverslips in 6‐well plates and serum‐starved for 24 hours. Cells were subsequently untreated or stimulated with EGF (Alexa Fluor 488 EGF complex, Life Technologies, Grand Island, NY). Cells were kept for 45 minutes at 4 °C in the dark, put back at 37 °C for 10 minutes, washed with some fresh medium to remove EGF and incubated again at 37 °C for 15 or 30 minutes. Cell were then fixed with paraformaldehyde 4% and preimmobilized with Triton X 0.3% for 10 minutes at room temperature. Cells were washed with PBS and blocked for 1 hour in 5% BSA in PBS.  They were subsequently incubated with an anti‐EEA1 or ‐LAMP1 antibody overnight at 4°C followed by 45 minutes of incubation at room temperature with an Alexa Fluor 594‐conjugated antibody (Dako, Glostrup, Germany). Images were acquired with the confocal system of Leica SP5 inverted microscope.

To assess autophagy, cells were seeded on coverslips in 6‐well plates. After transfection and treatment, cells were washed in PBS, fixed with 4% paraformaldehyde (PFA) in PBS for 15 minutes and preimmobilized with 0.2% Triton‐X100/PBS for 10 minutes at room temperature. Cells were then washed and blocked for 1 hour (5% BSA + 5% normal goat serum in PBS) followed by overnight incubation at 4 °C with primary antibodies in a blocking solution. The next day after 1 hour incubation with appropriate goat secondary antibodies coupled to Alexa Fluor 488 or 568 fluorophores (Life Technologies), coverslips were washed and incubated for 10 minutes with DAPI (Life Technologies). ProLong (Life Technologies) was used for mounting on glass slides and images were acquired with the Leica TCS SP5 II confocal system (Leica Microsystems, Wetzlar, Germany).

### Proliferation

Cell proliferation was measured by the incorporation of thymidine analogue EdU into newly synthesized DNAs by using the Click‐iT EdU Alexa Fluor 488 Flow Cytometry Assay Kit (Invitrogen ref: C10420) according to manufacturer's protocol. Briefly, cells were incubated with 10 µM EdU in complete medium for 2 hours. Following washing and harvesting by trypsinization, cells were fixed in Click‐iT fixative for 15 minutes and permeabilized by the saponin‐based buffer. Cells were incubated with Click‐iT reaction cocktail for 30 minutes at room temperature. Following washing steps, samples were analyzed on the FACS Canto (BD Biosciences) at the 488 nm excitation laser with a green emission filter. Results were analyzed with the FlowJo software.

### Targeted LC‐MS Metabolomics Analyses

MCF7 cells were transduced with lentiviral vectors (shControl, shSTARD7#2, and shSTARD7#4) in 6‐well plates and after selection in a Puromycin‐containing medium for 5 days, cells were washed with cold PBS, scrapped, centrifuged, and pellets were kept frozen in liquid nitrogen. For metabolomic analyses, the extraction solution was composed of 50% methanol, 30% acetonitrile (ACN) and 20% water. The volume of the extraction solution was adjusted to cell number (1 ml per 10^6^ cells). After addition of the extraction solution, samples were vortexed for 5 minutes at 4 °C and centrifuged at 16 000 × *g* for 15 minutes at 4 °C. The supernatants were collected and stored at –80 °C until analysis. LC/MS analyses were conducted on a QExactive Plus Orbitrap mass spectrometer equipped with an Ion Max source and a HESI II probe coupled to a Dionex UltiMate 3000 uHPLC system (Thermo). External mass calibration was performed using a standard calibration mixture every seven days, as recommended by the manufacturer. The 5 µl samples were injected onto a ZIC‐pHILIC column (150 mm  ×  2.1 mm; i.d. 5 µm) with a guard column (20 mm  ×  2.1 mm; i.d. 5 µm) (Millipore) for LC separation. Buffer A was 20 mM ammonium carbonate, 0.1% ammonium hydroxide (pH 9.2) and buffer B was ACN. The chromatographic gradient was run at a flow rate of 0.200 µl min^−1^ as follows: 0–20 minutes, linear gradient from 80% to 20% of buffer B; 20–20.5 minutes, linear gradient from 20% to 80% of buffer B; 20.5–28 minutes, 80% buffer B. The mass spectrometer was operated in full scan, polarity switching mode with the spray voltage set to 2.5 kV and the heated capillary held at 320 °C. The sheath gas flow was set to 20 units, the auxiliary gas flow to 5 units and the sweep gas flow to 0 units. The metabolites were detected across a mass range of 75–1000 *m*/*z* at a resolution of 35 000 (at 200 *m*/*z*) with the automatic gain control target at 10^6^ and the maximum injection time at 250 ms. Lock masses were used to ensure mass accuracy below 5 ppm. Data was acquired with Thermo Xcalibur software (Thermo). The peak areas of metabolites were determined using Thermo TraceFinder software (Thermo), identified by the exact mass of each singly charged ion and by the known retention time on the HPLC column.

### Subcellular Fractionations

Cytoplasmic and crude mitochondrial fractions were separated using the Mitochondrial Isolation Kit for Cultured Cells (Thermo Scientific) according to manufacturer's protocol. Briefly, cells from 15 cm culture dishes were scrapped on ice and pelleted by centrifugation 850 × *g* for 2 minutes. 800 µL of Reagent A with protease inhibitors was added, samples were vortexed for 5 seconds and incubated on ice for 2 minutes. Afterwards, 10 µL of reagent B was added and samples were vortexed at max speed for 5 seconds and incubated on ice for 5 minutes with vortexing every minute. A total of 800 µL of reagent C was added, and after centrifugation steps, supernatants – cytosolic fractions – were transferred to a fresh tube. 500 µL of reagent C was added to the remaining pellet and after centrifugation, the crude mitochondrial fraction was obtained. For Western blot analyses, mitochondrial pellets were suspended in 2% CHAPS in TBS.

For MAMs isolation,^[^ ^46^ ^]^ 60 (10 cm) dishes of control and STARD7‐depleted cells were washed twice with PBS (without Ca^2+^ and Mg^2+^), trypsinised and collected into 50 ml tubes. Samples were centrifuged at 600 × *g* for 5 minutes at 4 °C, washed again twice with PBS (without Ca^2+^ and Mg^2+^) and combined into two 50 ml tubes (control and STARD7‐depleted cells). Cells were again centrifuged at 600 × *g* for 5 minutes at 4 °C. PBS was discarded and cells were resuspended in 20 ml of ice cold buffer containing 225 mM mannitol, 75 mM sucrose, 0.1 mM EGTA, and 30 mM Tris‐HCl pH7.4. Cell were homogenized with cold Teflon homogenizer and cell integrity was monitored under the microscope (80–90% of cell damage has been achieved). Homogenates were centrifuged at 600 × *g* for 5 minutes at 4 °C and pellets were discarded. Homogenates were again centrifuged at 600 × *g* for 5 minutes at 4 °C. Supernatants were again centrifuged at 7000 × *g* for 10 minutes at 4 °C. The supernatant was a cytosolic fraction with lysosomes and microsomes, while the pellet contained mitochondria. The supernatant was further proceeded with separation of cytosolic, lysosomal and ER fractions. To achieve this goal, supernatants were centrifuged at 20 000 × *g* for 30 minutes at 4 °C. After centrifugation, pellets consisted of lysosomal and plasma membrane fractions. Next, we subjected the supernatant to centrifugation at 100 000 × *g* for 1 hour in order to isolate the ER (pellet) and the cytosolic fraction (supernatant). Crude mitochondria pellets were resuspended in 20 ml of cold buffer containing 225 mM mannitol, 75 mM sucrose, and 30 mM Tris‐HCl pH7.4 and centrifuged at 7000 × *g* for 10 minutes at 4 °C. Supernatants were discarded and pellets were resuspended in 20 ml of ice cold buffer containing 225 mM mannitol, 75 mM sucrose, and 30 mM Tris‐HCl pH7.4 and centrifuged at 10 000 × *g* for 10 minutes at 4 °C. Mitochondria pellets were resuspended in 2 ml of ice‐cold buffer (250 mM mannitol, 5 mM HEPES (pH 7.4) and 0.5 mM EGTA) and layered on 8 ml of Percoll medium. The same solution (250 mM mannitol, 5 mM HEPES (pH 7.4) and 0.5 mM EGTA) was layered gently on the top to fill up the centrifuge tube and was centrifuged at 95 000 × *g* for 30 minutes (Beckman Coulter Optima L‐100 XP Ultracentrifuge (SW40 rotor, Beckman, Fullerton, CA, USA). The band containing purified mitochondria was localized at the bottom of the ultracentrifuge tube. MAMs were visible as the diffused white band in the middle of the tube. MAMs were collected with a Pasteur pipette and then diluted 10 times with a buffer containing 250 mM mannitol, 5 mM HEPES (pH 7.4), and 0.5 mM EGTA. MAMs were centrifuged at 6300 × *g* for 10 minutes at 4 °C (to remove mitochondria contamination). Supernatant was then centrifuged at 100 000 × *g* for 1 hour at 4 °C. The pellet containing MAMs was resuspended and used for WB analyses.

### Transmission Electron Microscopy

Cells were fixed for 1 hour at 4 °C in a solution composed of 2.5% glutaraldehyde in 0.1 M Sorensen's buffer (0.2 M NaH_2_PO_4_, 0.2 M Na_2_HPO_4_, pH 7.4). After several washes in the same buffer, the samples were post‐fixed for 60 minutes with 2% osmium tetroxide, washed in deionized water, dehydrated through graded ethanol (70, 95, and 100%) and embedded in epon for 48 hours at 60 °C. Ultrathin sections (700‐A thick) were obtained by means of an ultramicrotome (Reichert Ultracut E) equipped with a diamond knife. The ultrathin sections were mounted on palladium/copper grids coated with collodion and contrasted with uranyl acetate and lead citrate for 5 minutes each before being examined under a Jeol JEM1400 transmission electron microscope at 80 kV. Random fields were photographed using an 11‐megapixel camera system (Quemesa, Olympus). Morphometric measurements were performed with iTEM v5.2 (Olympus, Tokyo, Japan) and analyzed using Image J v1.52a software.

To evaluate the area occupied by mitochondria in control or STARD7‐depleted cells, we randomly took 22 images of whole cells from each sample with a JEOL 1400 TEM at × 2500 magnification. In each image, we counted the number of mitochondria (651 in control cells and 393 in STARD7‐depleted cells for a total of 1044 mitochondria) and calculated the average number of mitochondria on the cytoplasmic area examined on each cell incidence and the average area occupied by the mitochondria on each cell incidence. In addition, we assessed the configuration of five mitochondria in 21 control cells and in 22 STARD7‐depleted cells, respectively.

To evaluate the frequency of contact between the mitochondria and the ER in control or STARD7‐depleted cells, we randomly took 20 images of cytoplasmic area from 21 different cells in each sample with a JEOL 1400 TEM at × 10 000 magnification. In each image, we counted the number of mitochondria (488 in control cells and 347 in STARD7‐depleted cells for a total of 835 mitochondria) and calculated the proportion of mitochondria in close contact with ER (<30 nm). Moreover, the perimeter of each mitochondria and the proportion of the mitochondrial surface closely associated with ER were calculated.

### Proteomic Analyses

Pellets were resuspended in 50 µl 6 M guanidine hydrochloride 100 mM Tris pH8.5 containing 1.5 mg ml^−1^ TCEP and 1 mg ml^−1^ chloroacetamide and digested with 1 µg LysC (FUJIFILM Wako Pure Chemicals U.S.A. Corporation) for 4 hours at 37 °C. Subsequently, samples were diluted to 300 µl with LC‐MS Water and digested overnight using 1 µg of porcine Trypsin (Thermo Scientific) followed by desalting using stage‐tips before MS analyses. Peptides were resuspended in 0.1% TFA in water and peptide content was estimated at 280 nm Absorption using a Nanodrop 2000 (Thermo Scientific). 1 µg was then injected and separated on an Ultimate 3000 Nano using a C18 packed emitter (Aurora, IonOptiks, Australia), with a gradient from 4% to 29% acetonitrile in 90 minutes, with a 10 minutes 80% wash. A total of 0.5% acetic acid was present throughout. Peptides were analyzed in data‐independent acquisition (DIA) mode on a Thermo Fusion Lumos. The mass spectrometer was operated in DIA mode, acquiring a MS 350–1650 Da at 120 k resolution followed by MS/MS on 45 windows with 0.5 Da overlap (200‐2000 Da) at 30 k with a NCE setting of 27. Data was searched using DIA‐NN (1.8.1) against the Uniprot Human database using the default setting for library‐free search. The mass spectrometry proteomics data have been deposited to the ProteomeXchange Consortium via the PRIDE^[^ ^1^ ^]^ partner repository with the dataset identifier PXD046984.

### H3K27Me3 ChIP Sequencing Analyses

Samples for chromatin immunoprecipitation were prepared using an iDeal ChIP‐seq Kit *for* Transcription Factors (Diagenode, Belgium) according to manufacturer's instructions. Briefly, 3 × 10^6^ cells (MCF7 shControl and shSTARD7#2) were fixed by directly adding the crosslinking reagent to the medium on the cell culture plate. After 15 minutes of fixation at room temperature, the reaction was stopped with Glycine. Cells were washed with PBS on ice and scrapped in the lysis buffer (1 × 10^6^ cells ml^−1^). After subsequent lysis steps, samples were immediately subjected to chromatin shearing using Bioraptor Plus (Diagenode) for 20 cycles (30 s “ON”, 30 s “OFF”) each at High power setting. ChIP of H3K27Me3 (Cell Signaling Technology (C36B11) Rabbit mAb #9733) was conducted on sheared protein from three independent experiments using the magnetic beads system. After elution, de‐crosslinking and DNA purification, the library for sequencing was prepared. Next‐generation sequencing was performed on all immunoprecipitated DNA samples and their respective inputs, with a depth of 50 million clusters per sample. For data analysis, clean reads were aligned to the hg38 human genome using Bowtie2 version 2.4.4 and SAMtools version 1.13 was used to index and sort binary alignment map (BAM) files.^[^ ^47^, ^48^ ^]^ Heatmaps were generated using the deepTools2 plotHeatmap function.^[^ ^49^ ^]^ Peak calling was performed for all samples using MACS2 version 2.2.7.1^[^ ^50^ ^]^ with the following parameter: callpeak ‐t file_merged.bam ‐c file_inp.sorted.bam ‐f BAMPE ‐g hs ‐n file_merged ‐broad ‐outdir peaks; peaks were annotated, and pathway analysis performed using ChIPseeker and clusterProfiler, respectively^[^ ^51^, ^52^ ^]^ in R v4 1.2 (http://www.r‐project.org/foundation/).

### Flow Cytometry

For the analysis of the lysosomal compartment, a cell‐permeable fluorescent dye LysoTracker Red DND‐99 (Invitrogen) was used. On day 5 post‐transduction, cells were washed with PBS and detached from a six well culture plate using a trypsin‐EDTA solution. Trypsin was inactivated by the addition of a culture medium with FBS. Cells were spinned down and suspended in a full culture medium containing Lysotracker dye in a final concentration of 100 nM and incubated at 37 °C for 30 minutes. Afterwards, cells were centrifuged and suspended in PBS for the analysis on the FACS Fortessa (BD). Analysis was performed using an excitation laser 561 nm and fluorescence was collected on the PE channel. Data was analyzed using the FlowJo software.

For FACS analyses to assess cell cycle progression, cells were seeded, trypsinized 24 hours later, collected and centrifuged at 200 × *g* for 5 minutes at room temperature. Cells were then washed once with PBS and fixed with 70% cold ethanol. They were vortexed to avoid cell aggregations and incubated minimum for 30 minutes on ice. Cells were then washed twice with PBS (500 × *g* for 5 minutes at 4 °C) and incubated with 400 µl of a solution containing 50 µg ml^−1^ PI and 50 µl of 100 µg ml^−1^ RNaseA in PBS per 10^6^ cells for 15 minutes at room temperature. Cells were acquired with the CytoFlex flow cytometer (Beckman Coulter) and analyzed with FlowJo10 with Cell Cycle detection/analysis. Cells were gated (PI positive, doublets and debris excluded) using the Watson Pragmatic algorithm.

### Statistical Analyses

For all analyses in which three groups were compared (shControl, shSTARD7#2 and shSTARD7#4), statistical significance was measured by one‐way ANOVA for repeated measures, using the Dunnett's multiple comparison test, unless stated otherwise. When only two groups were compared (shControl, shSTARD7#2), a Student's *T*‐Test was employed. All analyses were done using the Prism software. Significance: *p < 0.05, **p < 0.01, ***p < 0.001, ****p < 0.0001.

## Conflict of Interest

The authors declare no conflict of interest.

## Author Contributions

K.S. and A.C. equally contributed to this work and are co‐last authors. E.D. and A.C.: Conceptualization; E.D., C.M., A.H., S.O., P.A‐F., A.B., A.L., L.K., M.T., I.N., R.K., J.M. Jr., A. von K., N.H., K.S., and A.C.: Methodology; E.D., A.H., S.O., A.L., M.T., I.N., A. von K., N.H., and A.C.: Validation; E.D., A.L., M.T., I.N., A. von K., N.H., K.S., and A.C.: Formal Analysis; Investigation, E.D., C.M., P.A.‐F., A.B., A.L., L.K., M.T., I.N., R.K., J.M. Jr., A. von K., K.S., and A.C: Investigation; A.B., P.C., and G.P.: Resources; A.C.: Writing‐Original Draft; E.D., K.S., and A.C.: Writing‐Review & Editing; E.D., K.S., and A.C.: Visualization; A.C.: Supervision, Project administration and funding acquisition.

## Supporting information



Supporting Information

Uncropped gels STARD7 and breast cancer.

Supplementary file Densitometry graphs for Figures 1 to 10.

List of abbreviations.

## Data Availability

The data that support the findings of this study are available from the corresponding author upon reasonable request.
