# Supplementary material for: Loss of STARD7 Triggers Metabolic Reprogramming and Cell Cycle Arrest in Breast Cancer
Source: Adv Sci (Weinh). 2025 May 30;12(31):e03022. doi: 10.1002/advs.202503022 (PMC12376514; doi:10.1002/advs.202503022)
Supplement: Supplementary file 1 — Supporting Information [file ADVS-12-e03022-s004.docx]

**
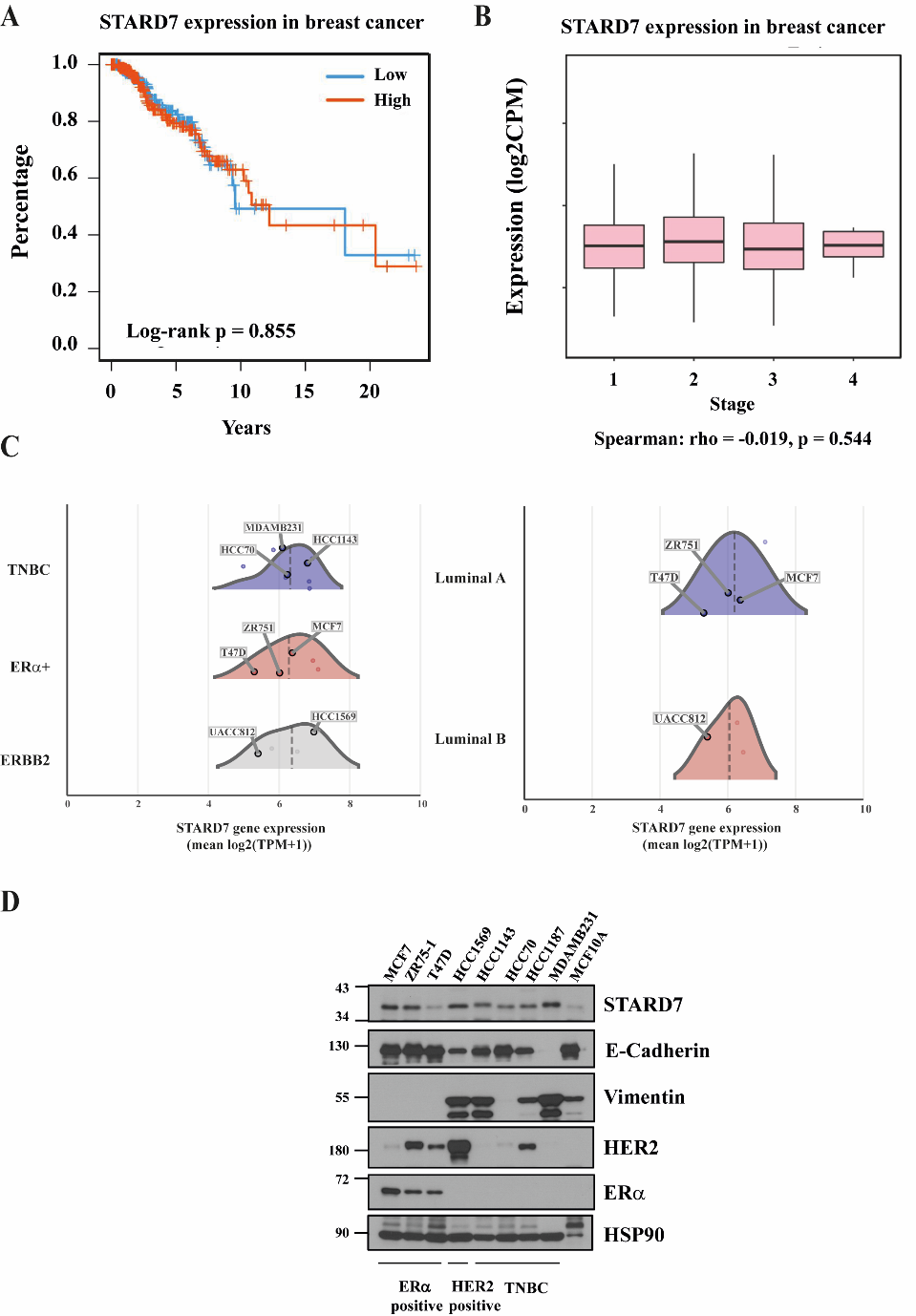
**

**Supplementary Figure 1: STARD7 expression in breast cancer. A.** and **B.** STARD7 expression is not correlated to patient survival nor to the stage of the disease (A and B, respectively). Data were acquired from the TISIDB database (<http://cis.hku.hk/TISIDB/index.php>). **C.** Density plot of STARD7 mRNA expression levels between Triple Negative Breast Cancer (TNBC), ERα^+^ and ERBB2^+^ cell lines or between Luminal A and Luminal B breast cell lines using normalized read count data (TPM+1) from DepMap dataset (left and right panels, respectively). **D.** STARD7 expression at the protein level in human breast cancer-derived cell lines as well as in MCF10A cells (WB analyses). Note that HCC1187 cells have been described as a TNBC cell line in which we nevertheless detected HER2 expression.

**
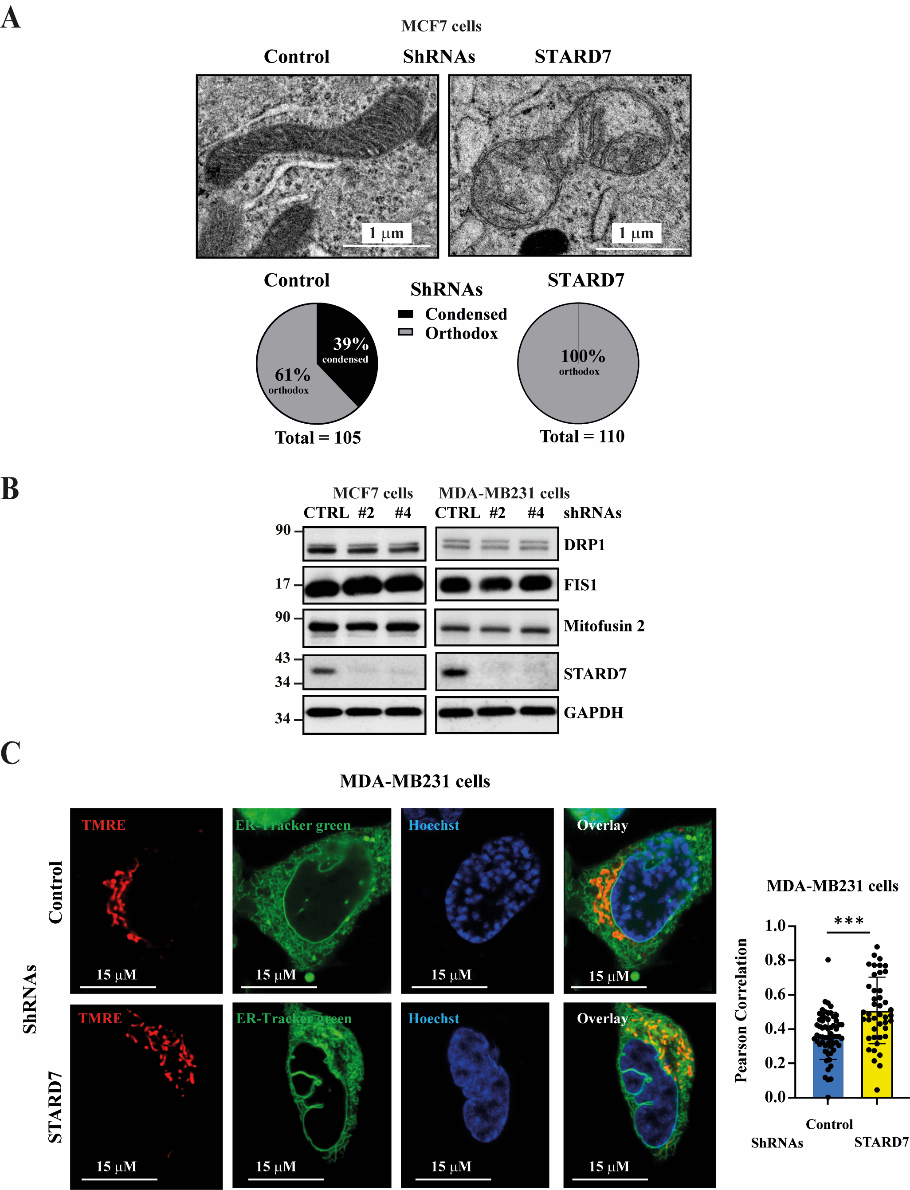
**

**Supplementary Figure 2: STARD7 deficiency leads to changes in mitochondrial morphology in breast cancer cells and to enhanced mitochondrial-ER contacts. A.** Accumulation of mitochondria in the orthodox state upon STARD7 deficiency in MCF7 cells. On the top, the ultrastructure of mitochondria in control and STARD7-depleted breast cancer cells is illustrated. At the bottom, the percentage of mitochondria in orthodox or condensed state is mentioned in control and STARD7-depleted breast cancer cells (see methods for details). **B.** STARD7 is dispensable in mitochondria dynamics. Extracts from control and STARD7-depleted MCF7 and MDA-MB231 cells were subjected to western blot analyses using the indicated antibodies. **C.** Enhanced mitochondrial-ER contacts upon STARD7 deficiency. Control and STARD7-depleted MDA-MB231 cells were co-stained with the marker of active mitochondria TMRE and with ER-Tracker green (Endoplasmic Reticulum marker) in order to show contact sites between both mitochondria and ER. Hoechst stainings were done to show the nucleus. Illustrated graphs represent the degree of colocalization between ER and mitochondria, using the Pearson's coefficient. 61 control and 42-depleted MDA-MB231 cells were analyzed. The Prism10 program was used for statistical analyses (Welch’s t-test, ** = p <0.01).

**
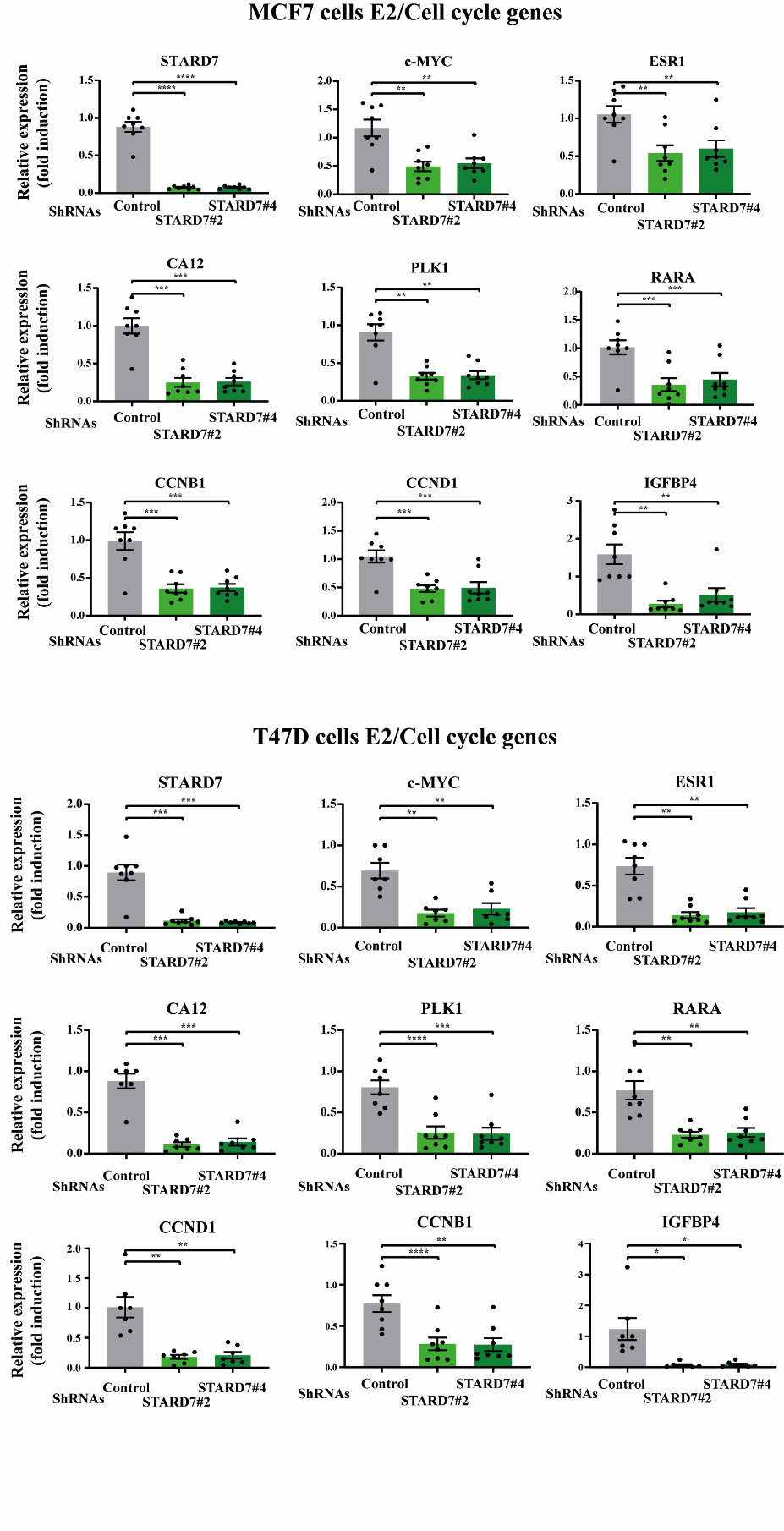
**

**Supplementary Figure 3: STARD7 deficiency leads to transcriptional reprogramming.** Real-Time PCR analyses were conducted with total RNAs from control and STARD7-depleted MCF7 or T47D cells, as indicated. The expression of each candidate in control cells was set to 1 and levels in cells lacking STARD7 was relative to that after normalization with GAPDH mRNA levels (mean +/- SD, one-way ANOVA, * = p <0.05, ** = p < 0.01, *** = p < 0.001, n = 8 distinct experiments).


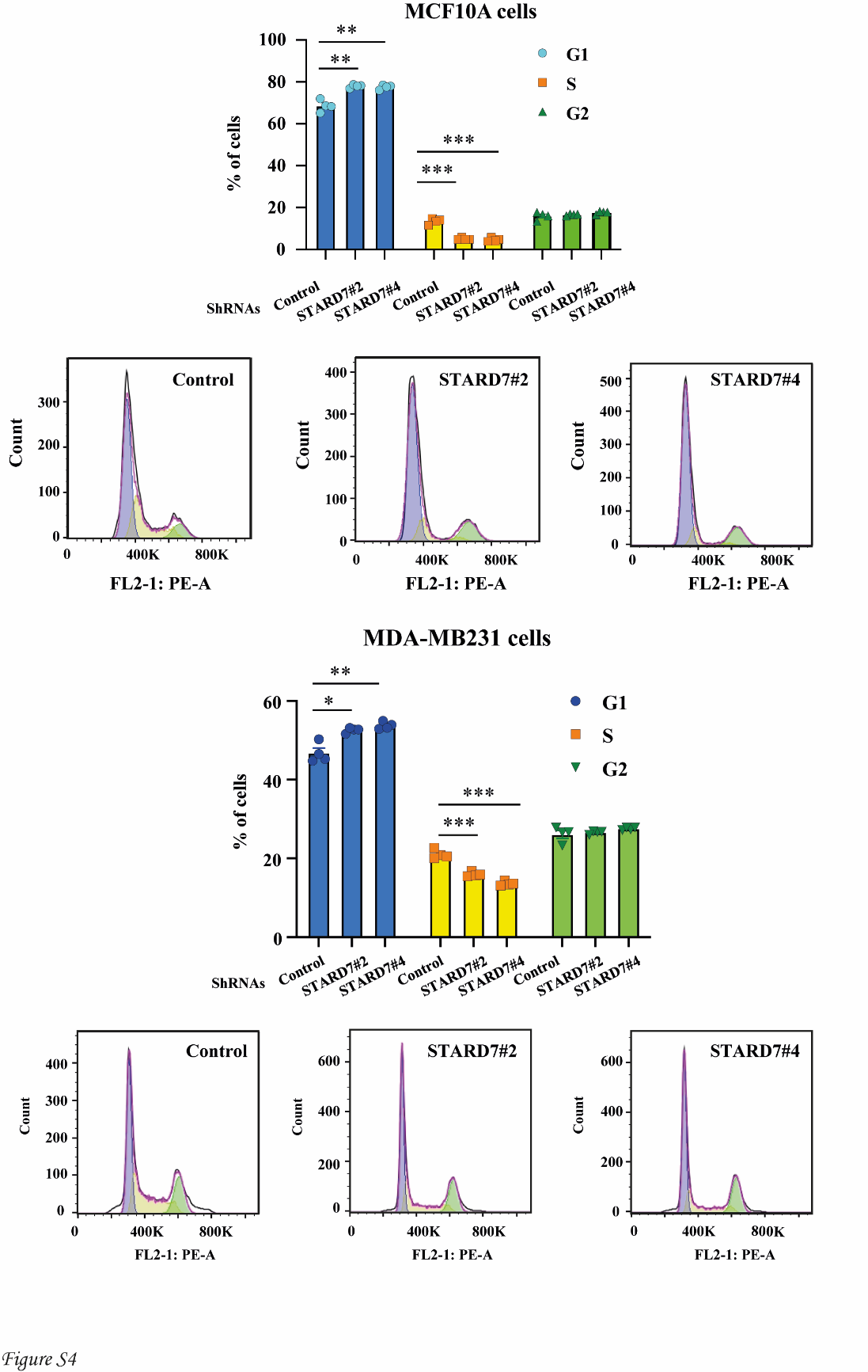


**Supplementary Figure 4: Defects in cell cycle progression upon STARD7 deficiency in breast cancer cells.** The percentage of cells in G1, S and G2 phases in control and STARD7-depleted MCF10A or MDA-MB231 cells was quantified by FACS. Two experiments carried out in duplicates are shown and 10^4^ cells were analyzed for each experimental condition (Student T-Test, * = p < 0.05, ** = p < 0.01, *** = p < 0.001, **** = p < 0.0001).

**
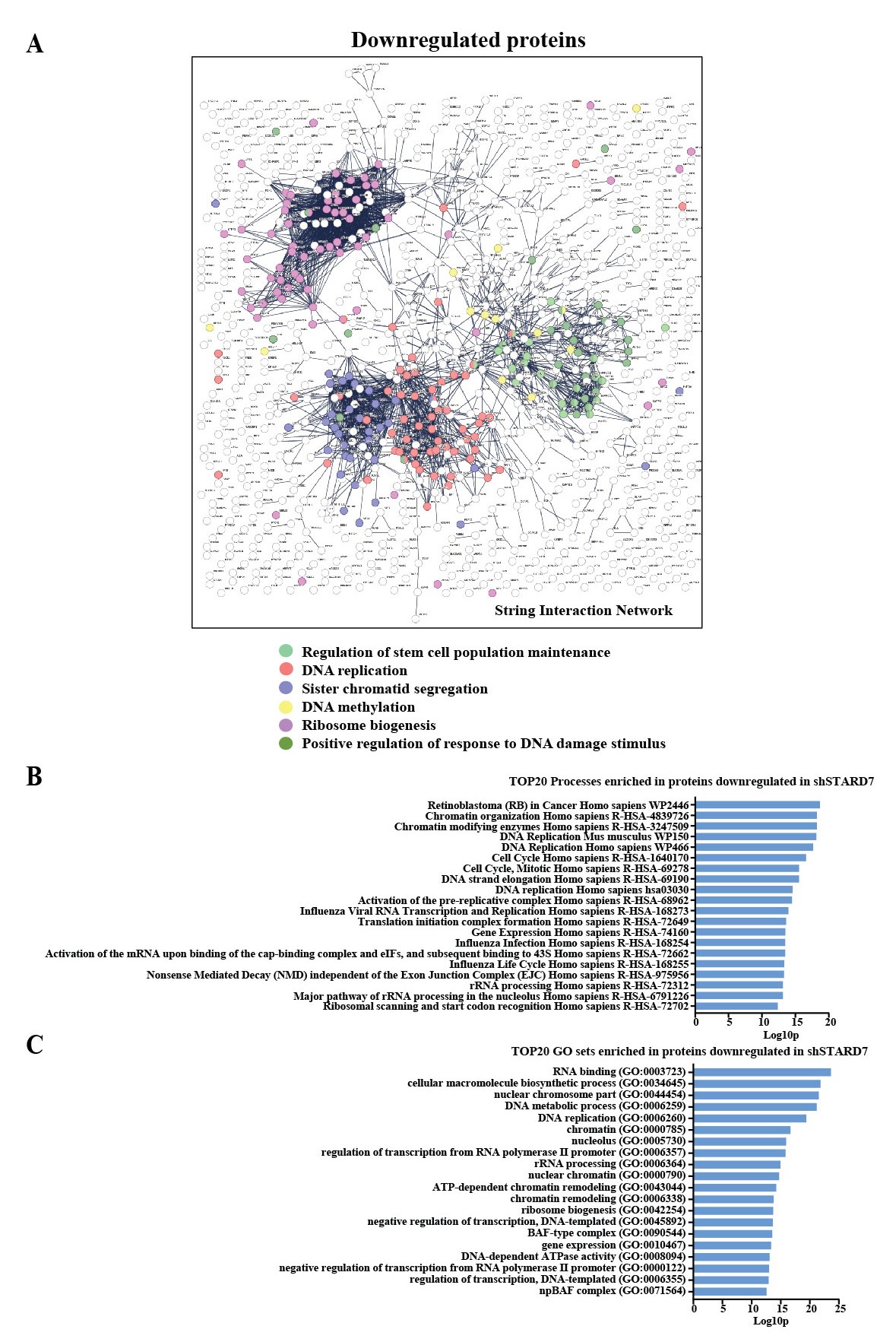
**

**Supplementary Figure 5: STARD7 deficiency decreases levels of proteins linked to cell cycle progression.** A string Interaction Network (A) and the top 20 processes and GO sets (C and B, respectively) enriched in breast cancer-derived MCF7 cells lacking STARD7 are illustrated, based on proteomic analyses carried out with extracts from control and STARD7-depleted MCF7 cells.


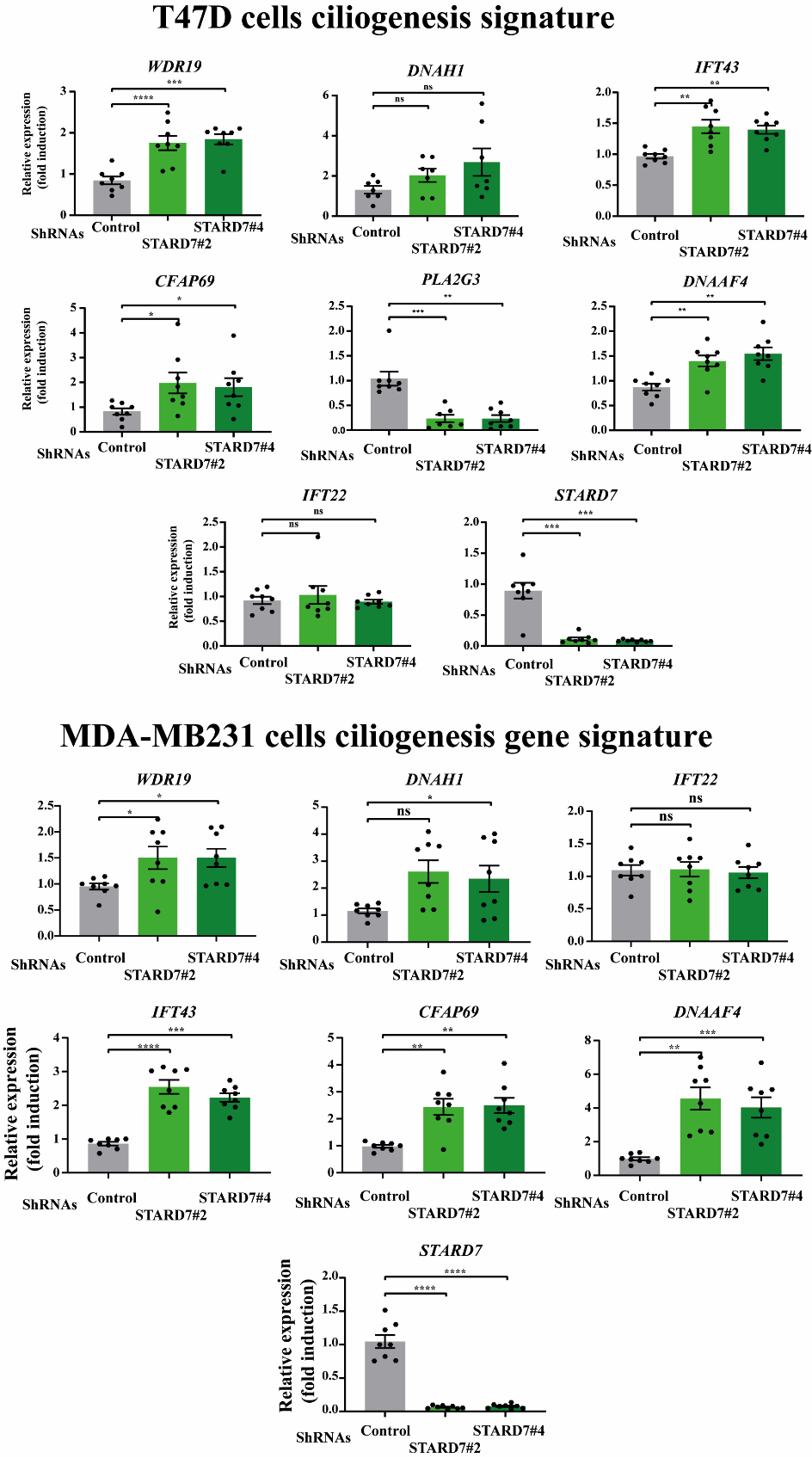


**Supplementary Figure 6: A ciliogenesis signature is linked to STARD7 deficiency in breast cancer cells.** Real-Time PCR analyses were conducted with total RNAs from control and STARD7-depleted T47D or MDA-MB231 cells. The expression of each candidate in control cells was set to 1 and levels in cells lacking STARD7 was relative to that after normalization with GAPDH mRNA levels (mean +/- SD, one-way ANOVA, * = p <0.05, ** = p < 0.01, *** = p < 0.001, n = 8 distinct experiments).

**
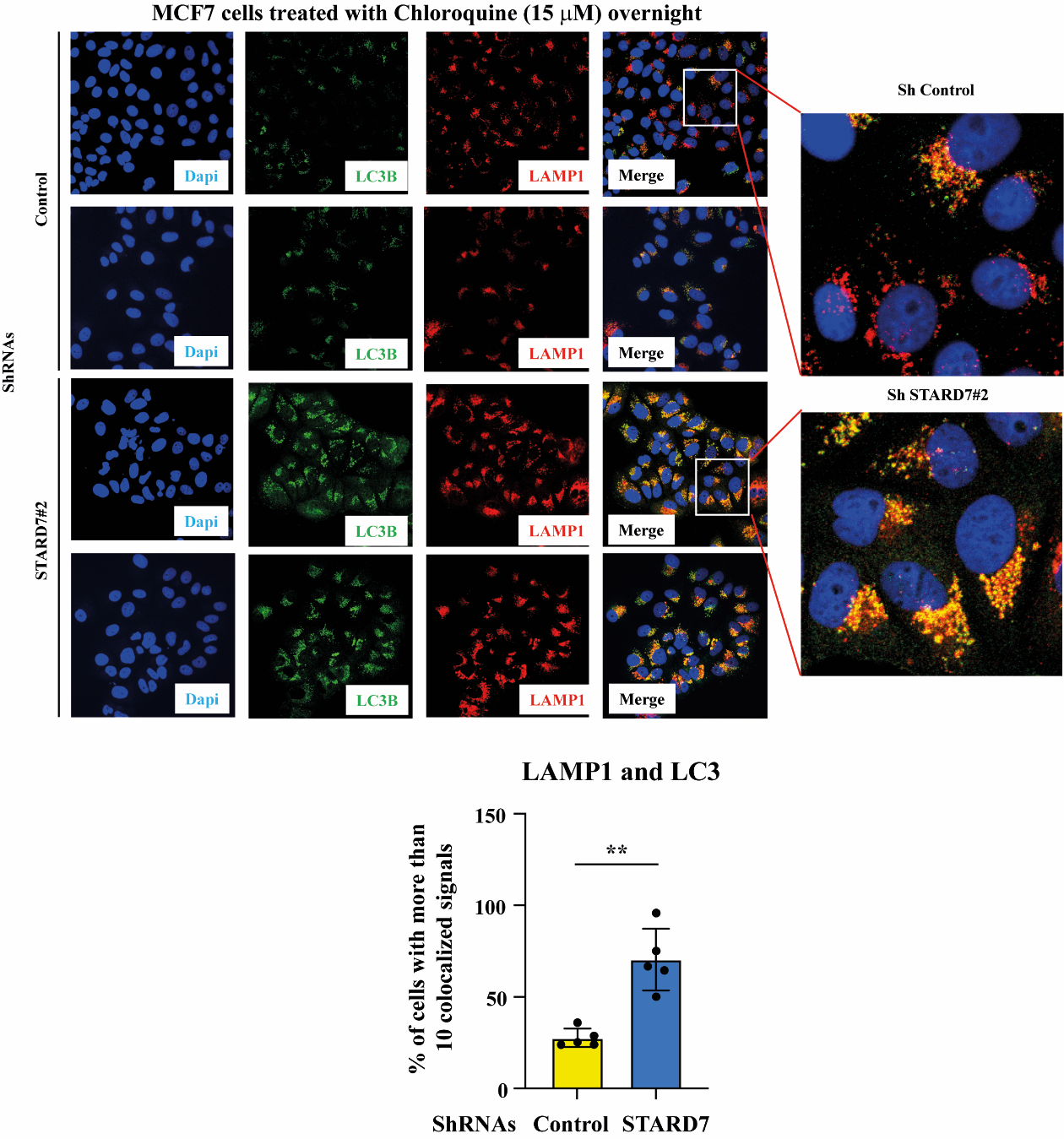
**

**Supplementary Figure 7: STARD7-depleted breast cancer cells undergo autophagy.** Representative immunofluorescence analyses of control and STARD7-depleted MCF7 cells (shRNA Control and shRNA STARD7#2) after an overnight treatment with Chloroquine (15 μM). Anti-LAMP1 (red) and -LC3B stainings were carried out. DAPI was used to for nuclei stainings. The bar graph shows a quantitative analysis of cells undergoing autophagy. The quantification shows the percentage of cells with more than 10 co-localized points to the total number of analysed cells (mean +/- SD, Student T-test, ** = p < 0.01).

**
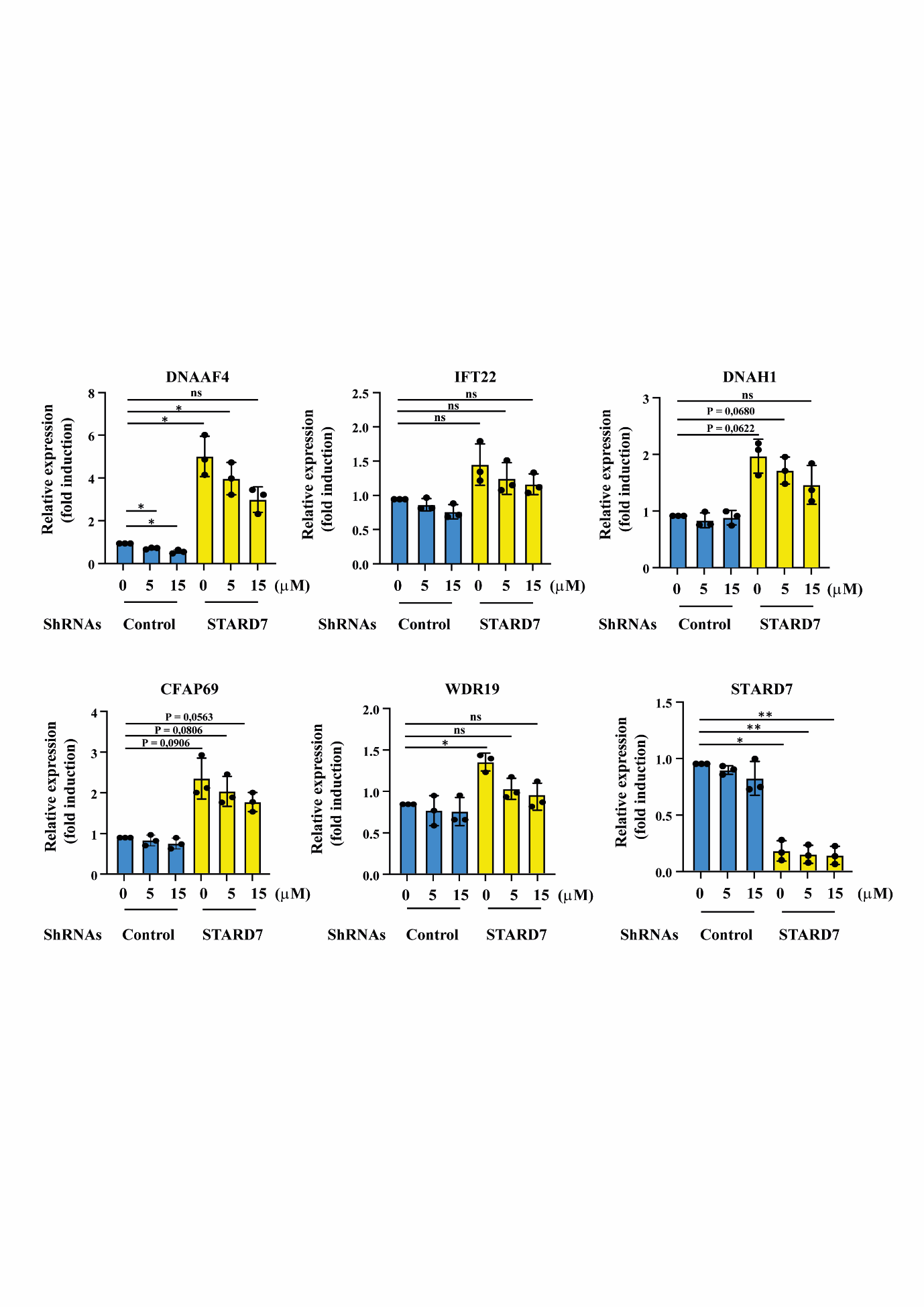
**

**Supplementary Figure 8: STARD7-depleted breast cancer cells undergo autophagy-dependent ciliogenesis.** Real-Time PCR analyses were conducted with total RNAs from control and STARD7-depleted MCF7 cells treated or not with Chloroquine at the indicated concentrations. The expression of each candidate in unstimulated control cells was set to 1 and levels in other experimental conditions were relative to that after normalization with GAPDH mRNA levels (mean +/- SD, RM one-way ANOVA, Dunnett’s multiple comparison test, * = p <0.05, ** = p < 0.01, *** = p < 0.001, n = 3 distinct experiments performed in triplicates).


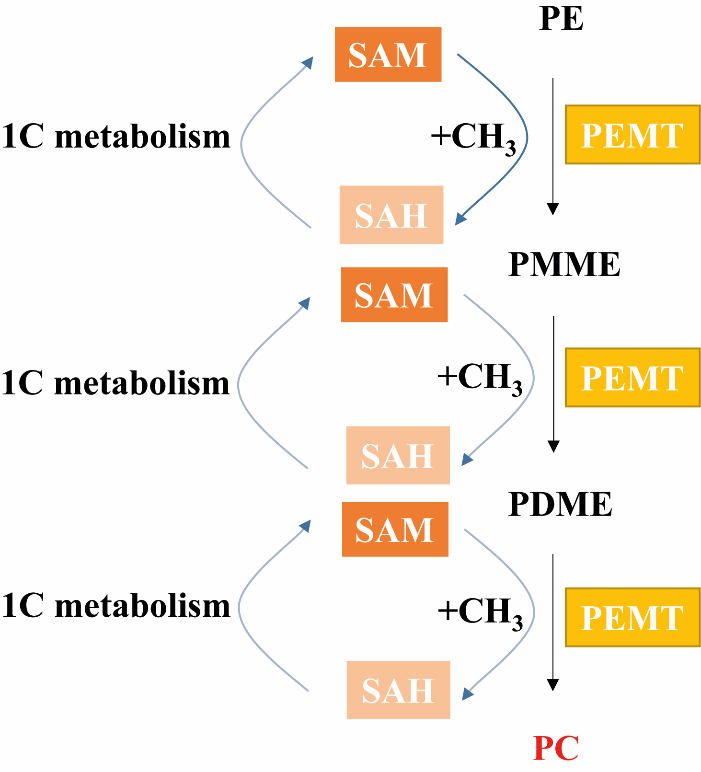


**Supplementary Figure 9: Enzymatic cascade involved in Phosphatidylcholine (PC) synthesis.** The enzyme involved in all reactions is PEMT (Phosphatidylethanolamine N-Methyl Transferase) while substrates are Phosphatidylethanolamine (PE), Phosphatidylmonomethylethanolamine (PMME) and Phosphatidyldi- methylethanolamine (PDME).

**Reference:**

1 Curtis C, Shah S, Chin S-F, Turashvili G, Rueda O, Dunning M *et al.* The genomic and transcriptomic architecture of 2,000 breast tumours reveals novel subgroups. *Nature* 2012; **486**: 346–352.
