# Supplementary material for: Loss of STARD7 Triggers Metabolic Reprogramming and Cell Cycle Arrest in Breast Cancer
Source: Adv Sci (Weinh). 2025 May 30;12(31):e03022. doi: 10.1002/advs.202503022 (PMC12376514; doi:10.1002/advs.202503022)

ADVANCED SCIENCE
Open Access

Supporting Information

for *Adv. Sci.*, DOI 10.1002/advs.202503022

Loss of STARD7 Triggers Metabolic Reprogramming and Cell Cycle Arrest in Breast Cancer

*Ewelina Dondajewska, Paula Allepuz-Fuster, Chloé Maurizy, Alexandre Hego, Sandra Ormenese, Quentin Lion, Arnaud Blomme, Pierre Close, Arnaud Lavergne, Latifa Karim, Marc Thiry, Ivan Nemazanyy, Roopesh Krishnankutty, Jair Marques Jr, Alex von Kriegsheim, Nathaniel F. Henneman, Ganna Panasyuk, Kateryna Shostak and Alain Chariot**

Figure 1G

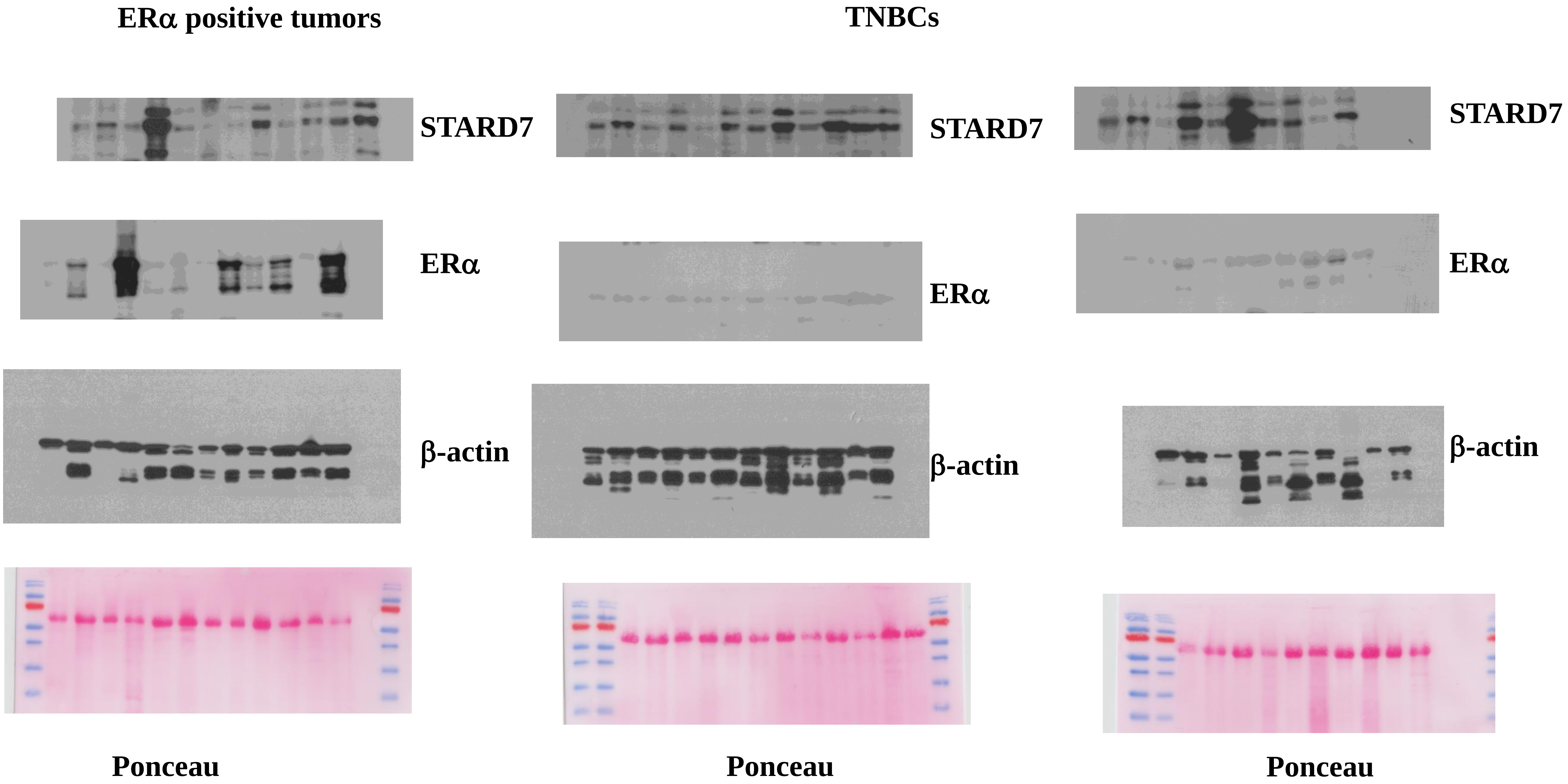
ERα positive tumors
TNBCs
STARD7
STARD7
STARD7
ERα
ERα
ERα
β-actin
β-actin
β-actin
Ponceau
Ponceau
Ponceau

Figure 2A

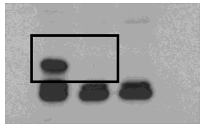

STARD7

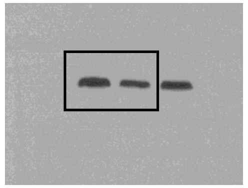

HSP90

Figure 2D

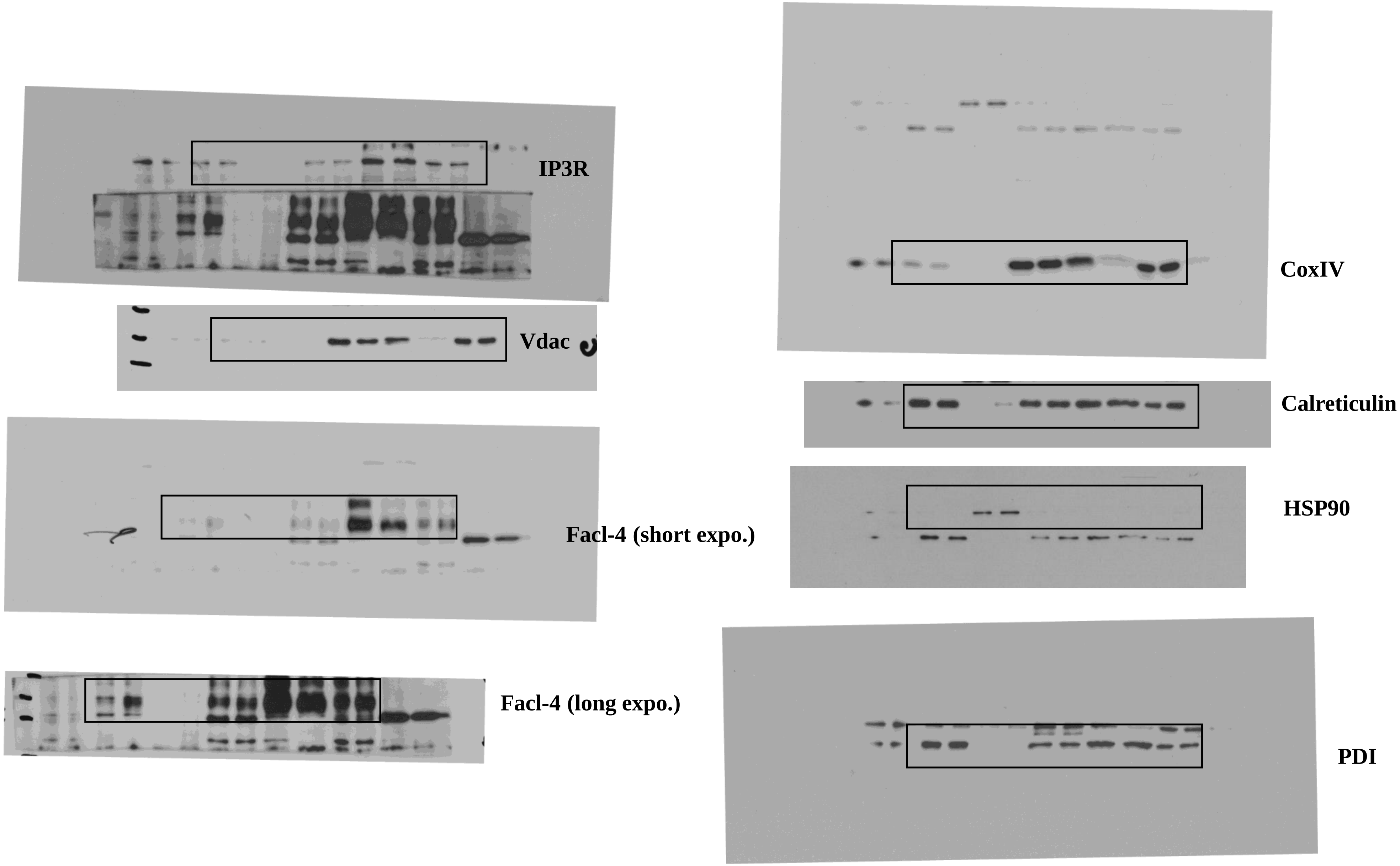
IP3R
Vdac
Facl-4 (short expo.)
Facl-4 (long expo.)
CoxIV
Calreticulin
HSP90
PDI

Figure 2D

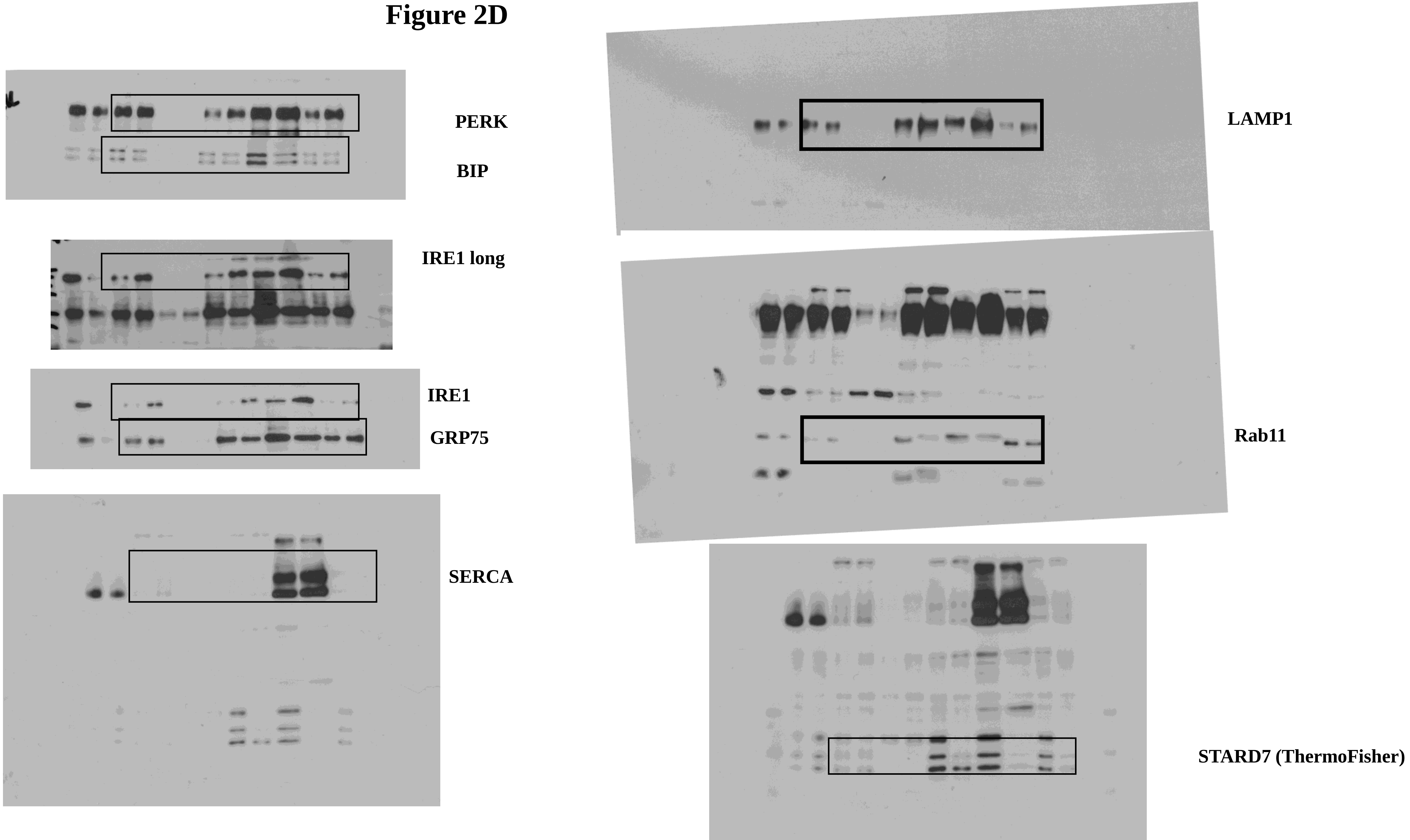
PERK
BIP
IRE1 long
IRE1
GRP75
SERCA
LAMP1
Rab11
STARD7 (ThermoFisher)

Figure 2E

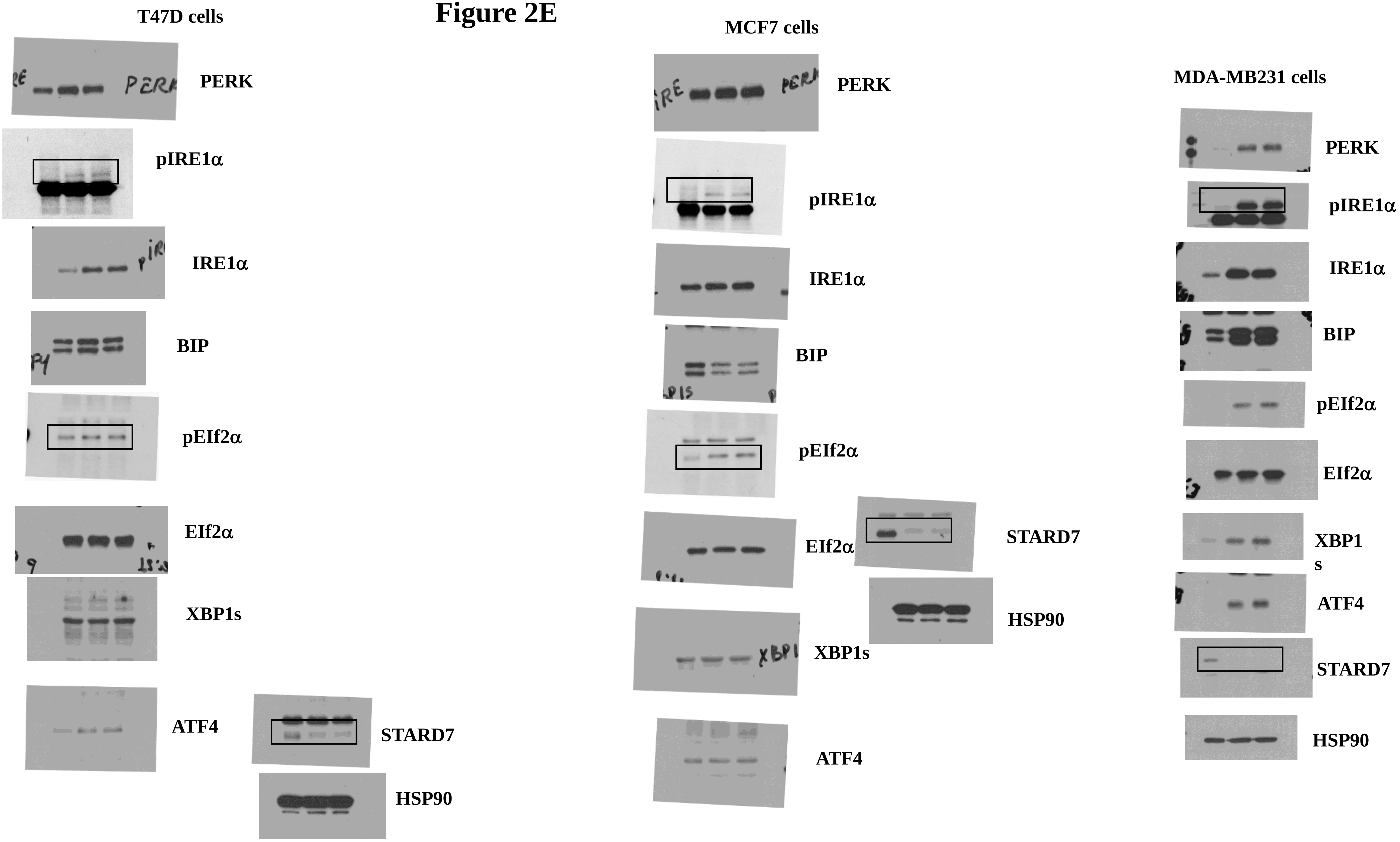

T47D cells
PERK
pIRE1α
IRE1α
BIP
pEIf2α
EIf2α
XBP1s
ATF4
STARD7
HSP90
MCF7 cells
PERK
pIRE1α
IRE1α
BIP
pEIf2α
EIf2α
STARD7
HSP90
XBP1s
ATF4
MDA-MB231 cells
PERK
pIRE1α
IRE1α
BIP
pEIf2α
EIf2α
XBP1s
ATF4
STARD7
HSP90

Figure 3C

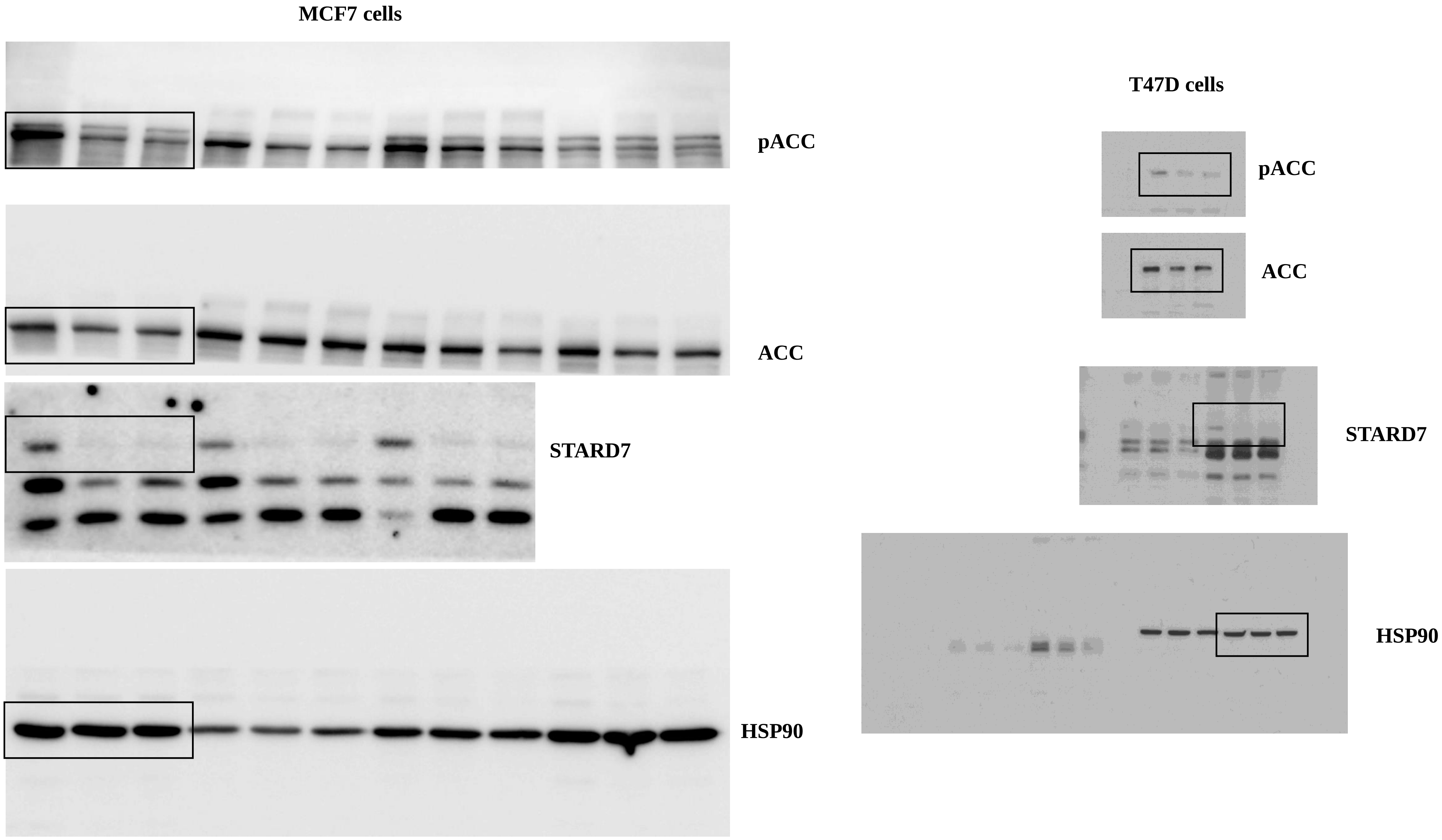

MCF7 cells
T47D cells
pACC
pACC
ACC
ACC
STARD7
STARD7
HSP90
HSP90

Figure 3C

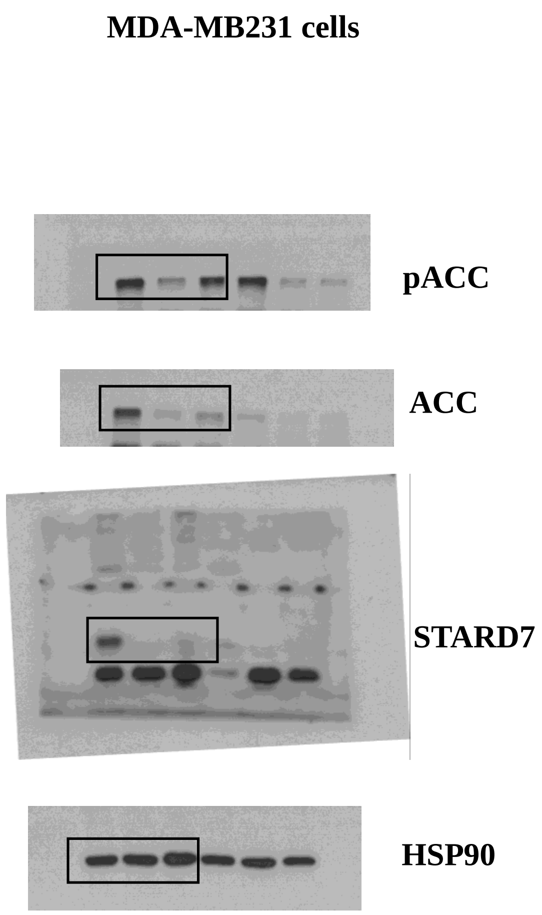

Figure 3G

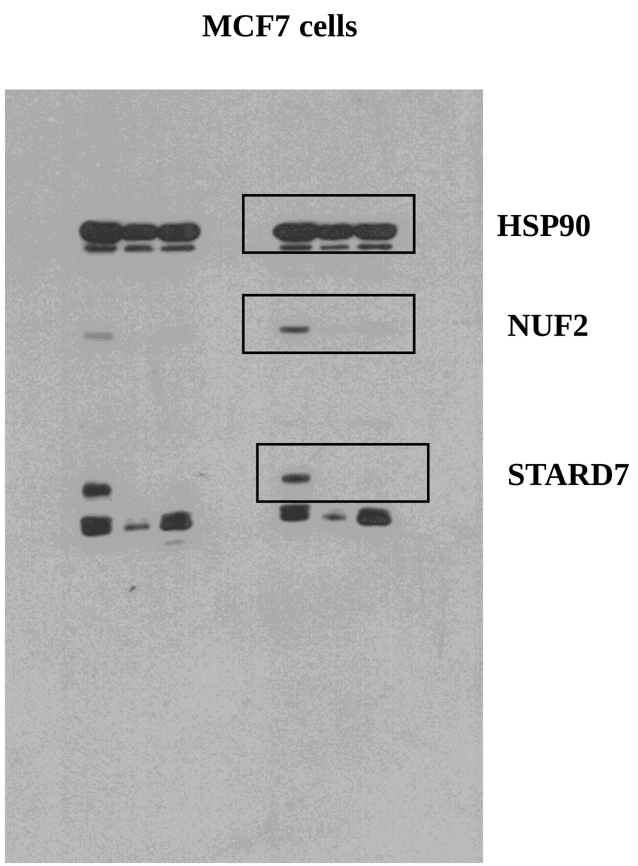

Figure 3H

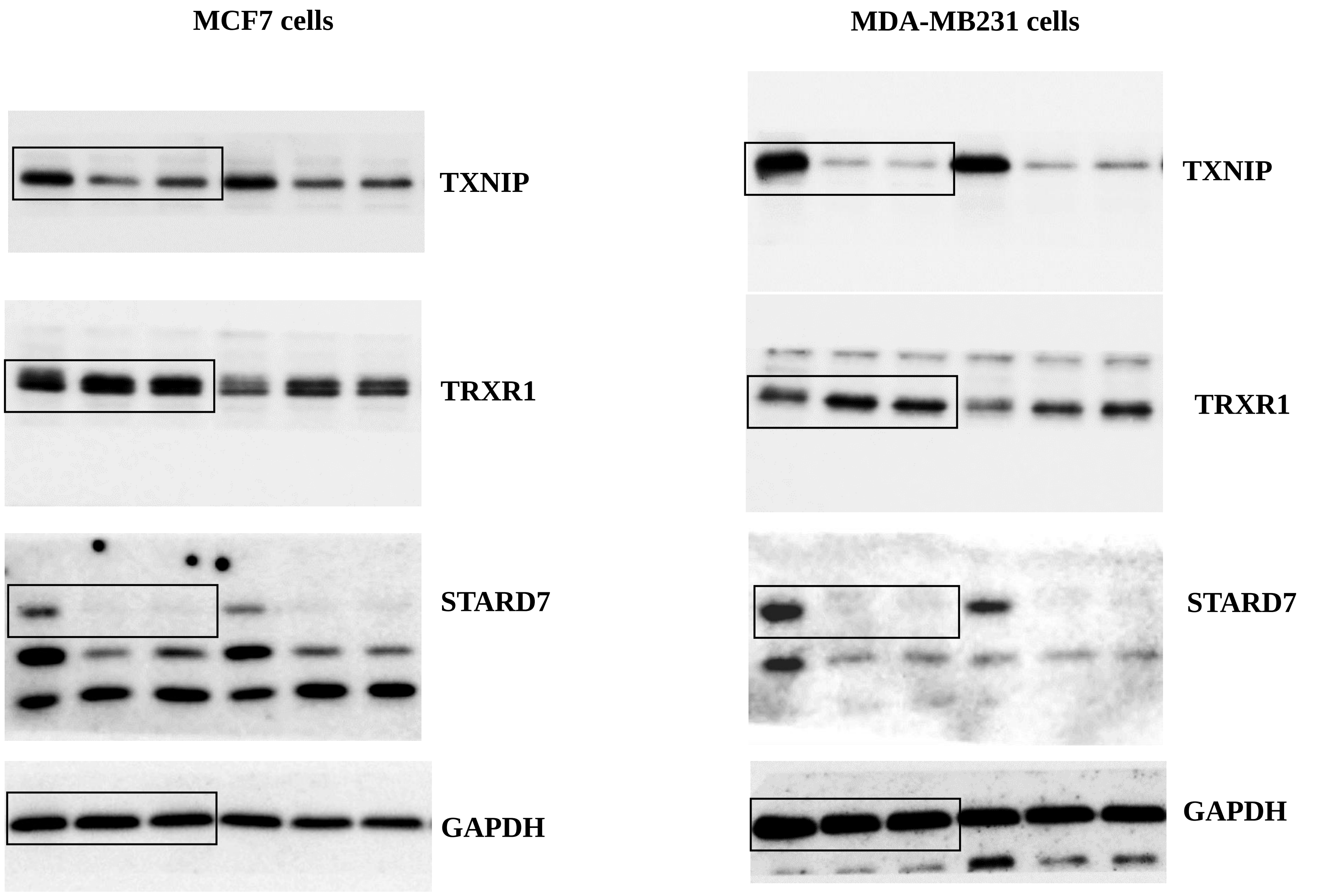

MCF7 cells
MDA-MB231 cells
TXNIP
TRXR1
STARD7
GAPDH
TXNIP
TRXR1
STARD7
GAPDH

Figure 4E

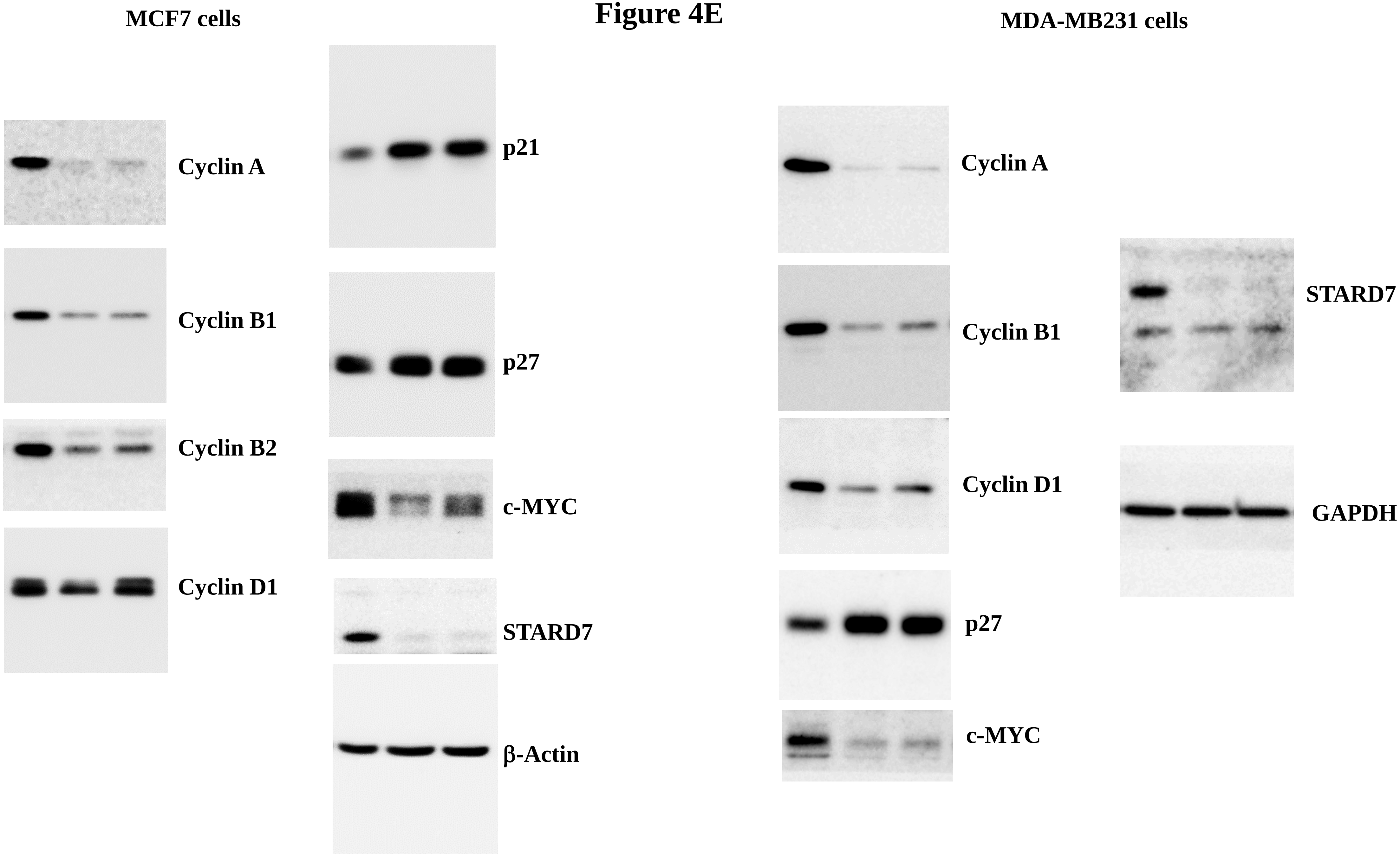
MCF7 cells
Cyclin A
Cyclin B1
Cyclin B2
Cyclin D1
p21
p27
c-MYC
STARD7
β-Actin
MDA-MB231 cells
Cyclin A
Cyclin B1
Cyclin D1
p27
c-MYC
STARD7
GAPDH

Figure 5C

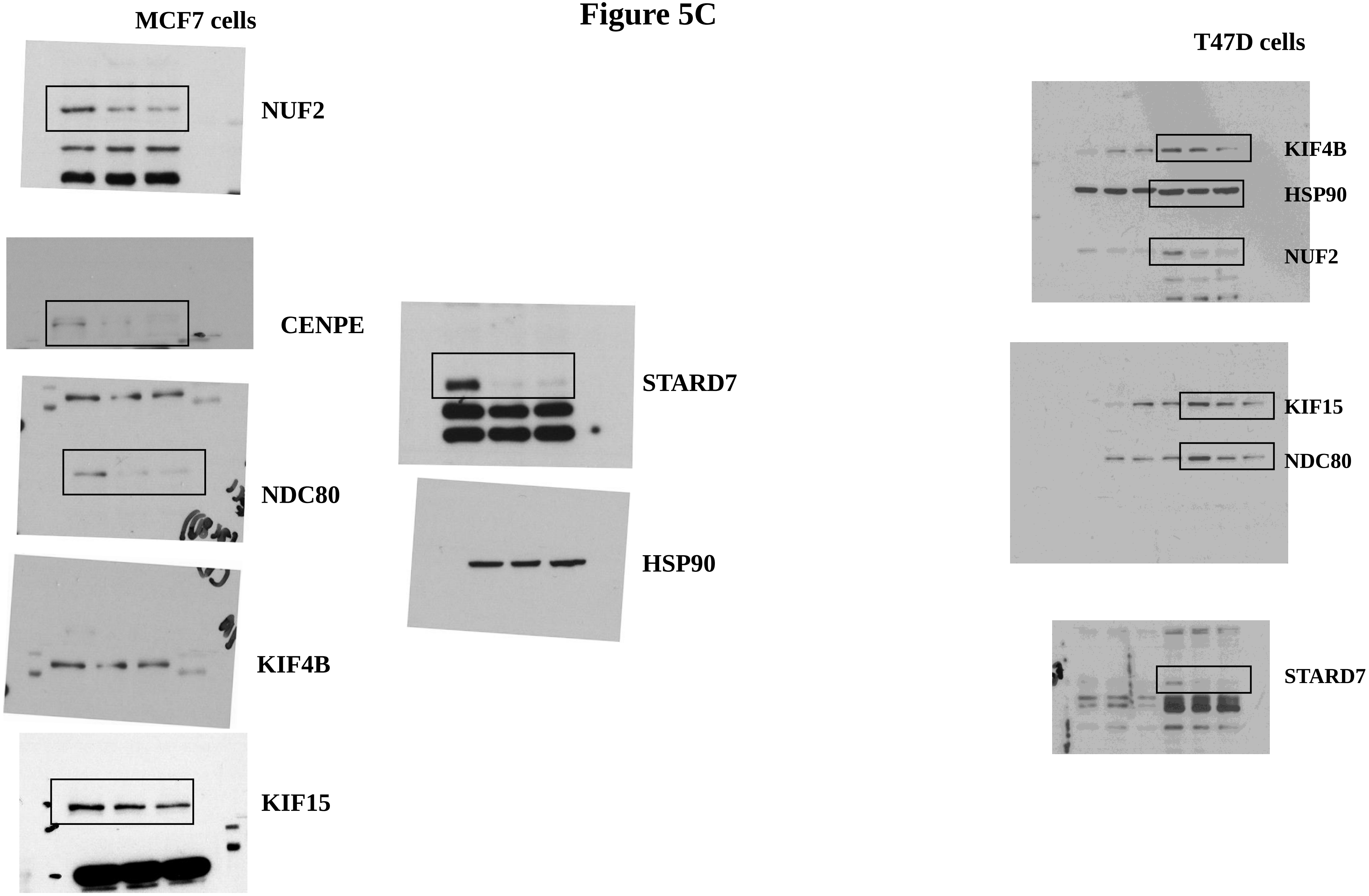
MCF7 cells
NUF2
CENPE
NDC80
KIF4B
KIF15
STARD7
HSP90
T47D cells
KIF4B
HSP90
NUF2
KIF15
NDC80
STARD7

Figure 5C

BT549 cells

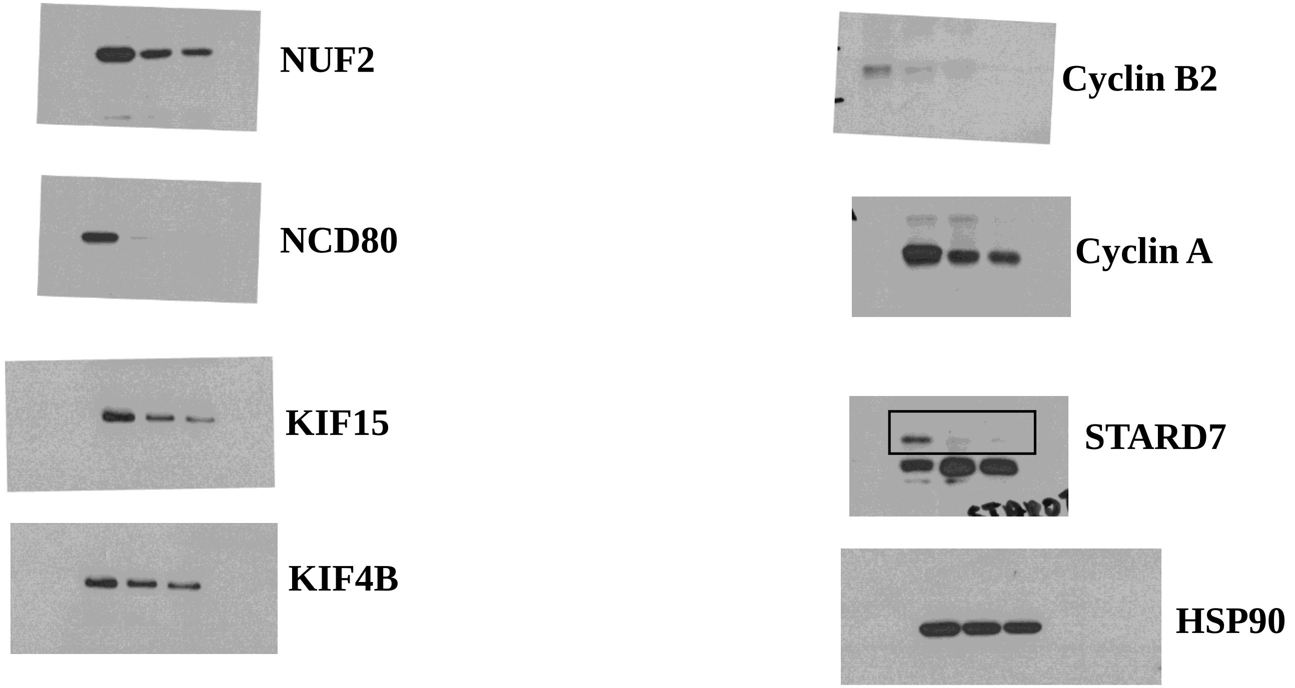

Figure 6A

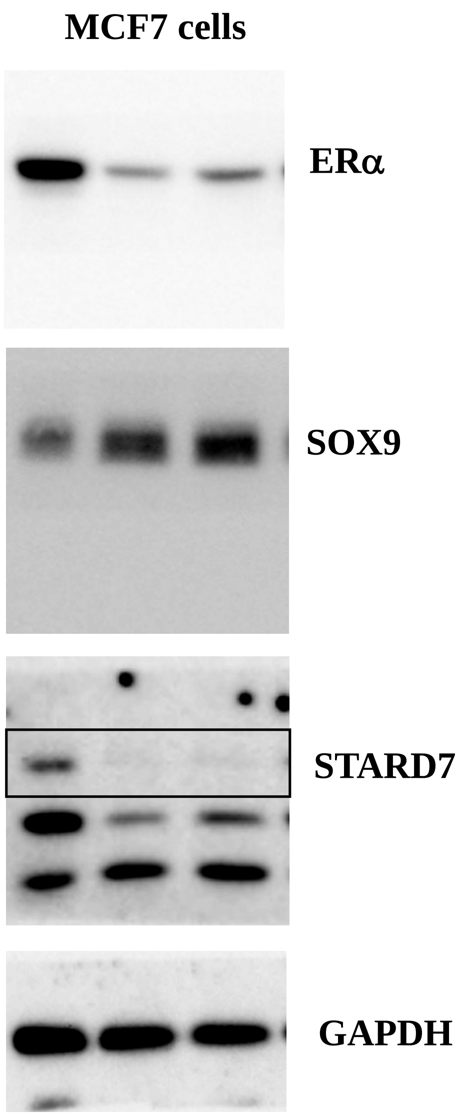

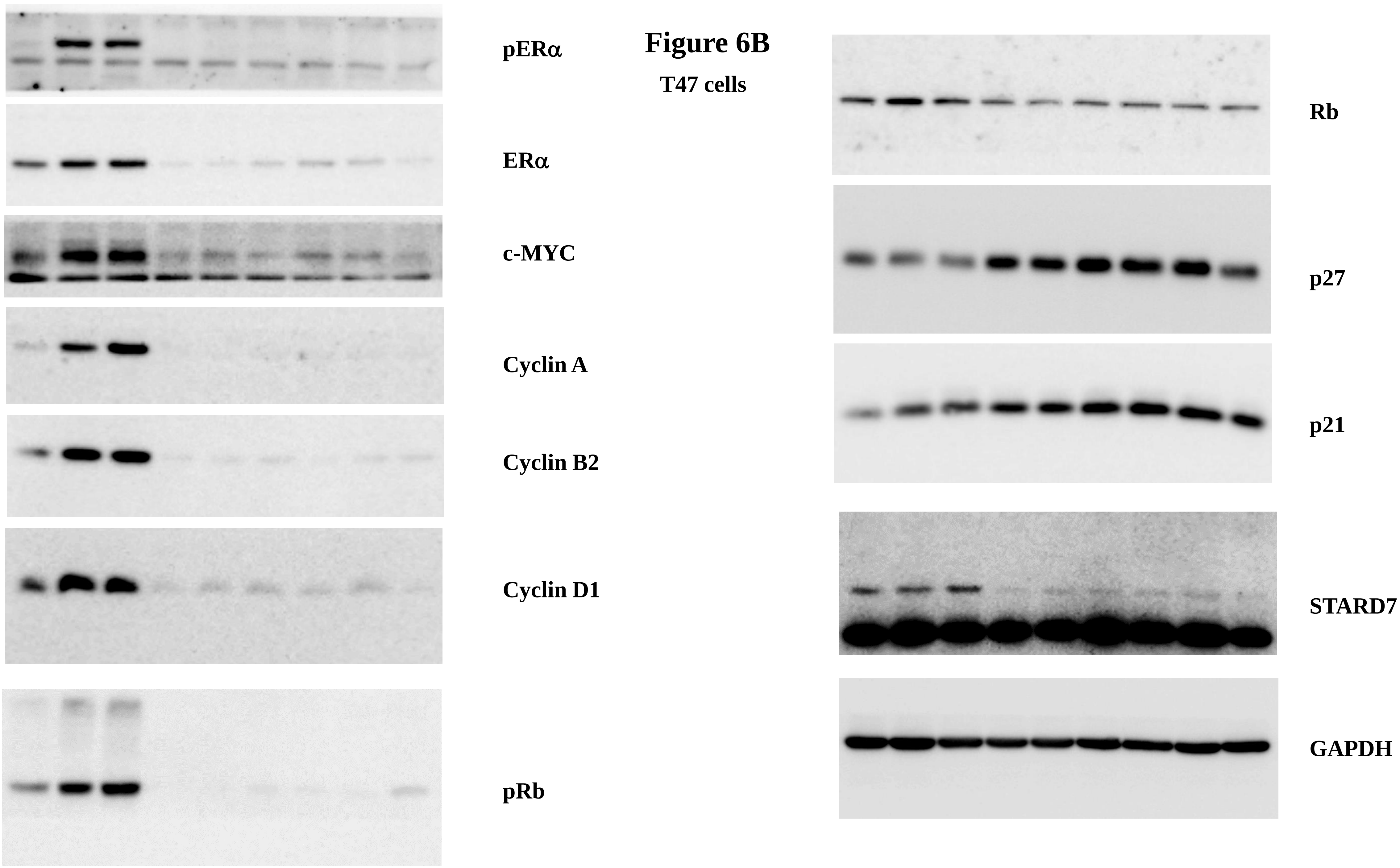
Figure 6B
T47 cells
pERα
ERα
c-MYC
Cyclin A
Cyclin B2
Cyclin D1
pRb
Rb
p27
p21
STARD7
GAPDH

Figure 6B

MCF7 cells

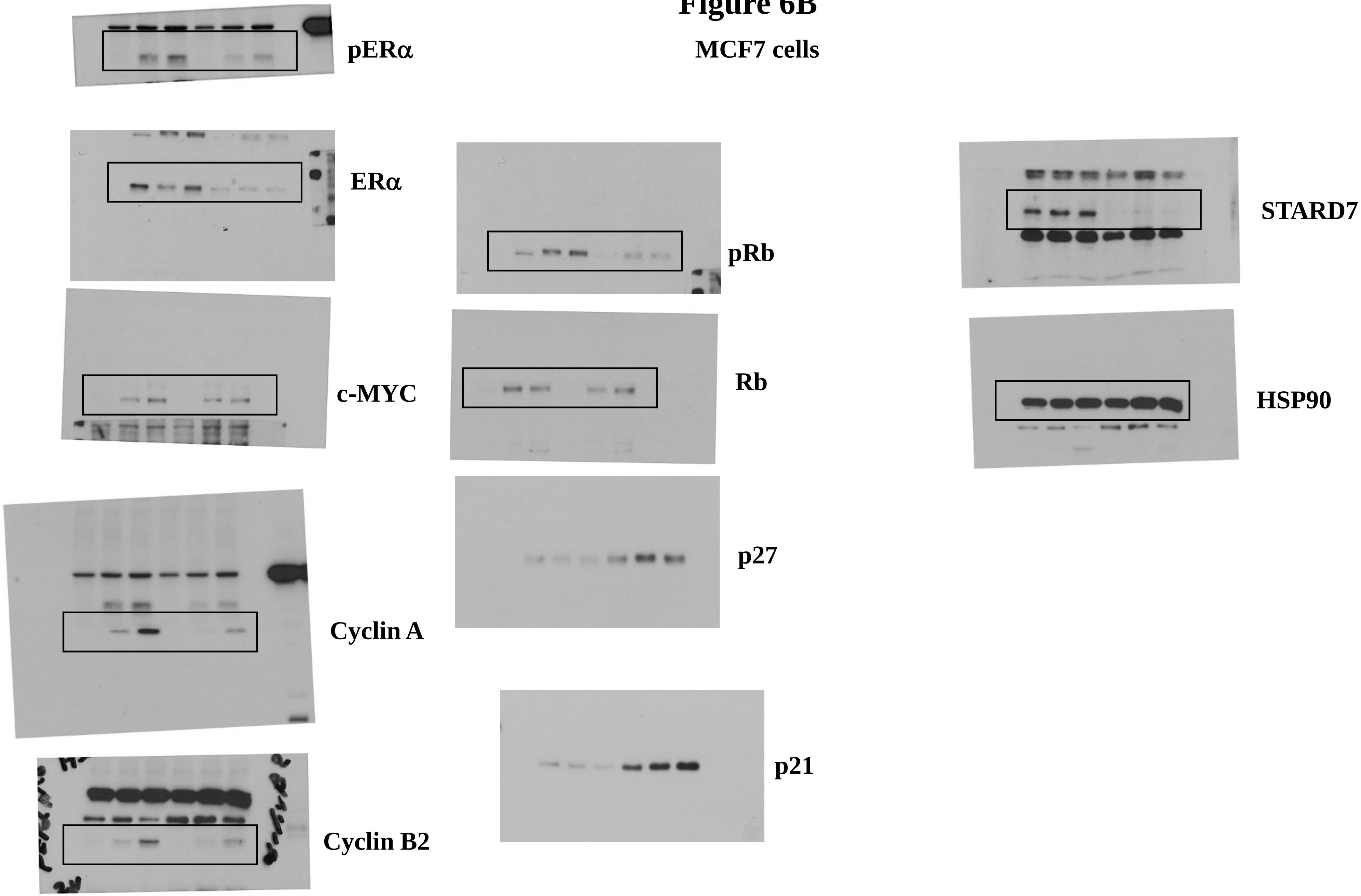

Figure 7A

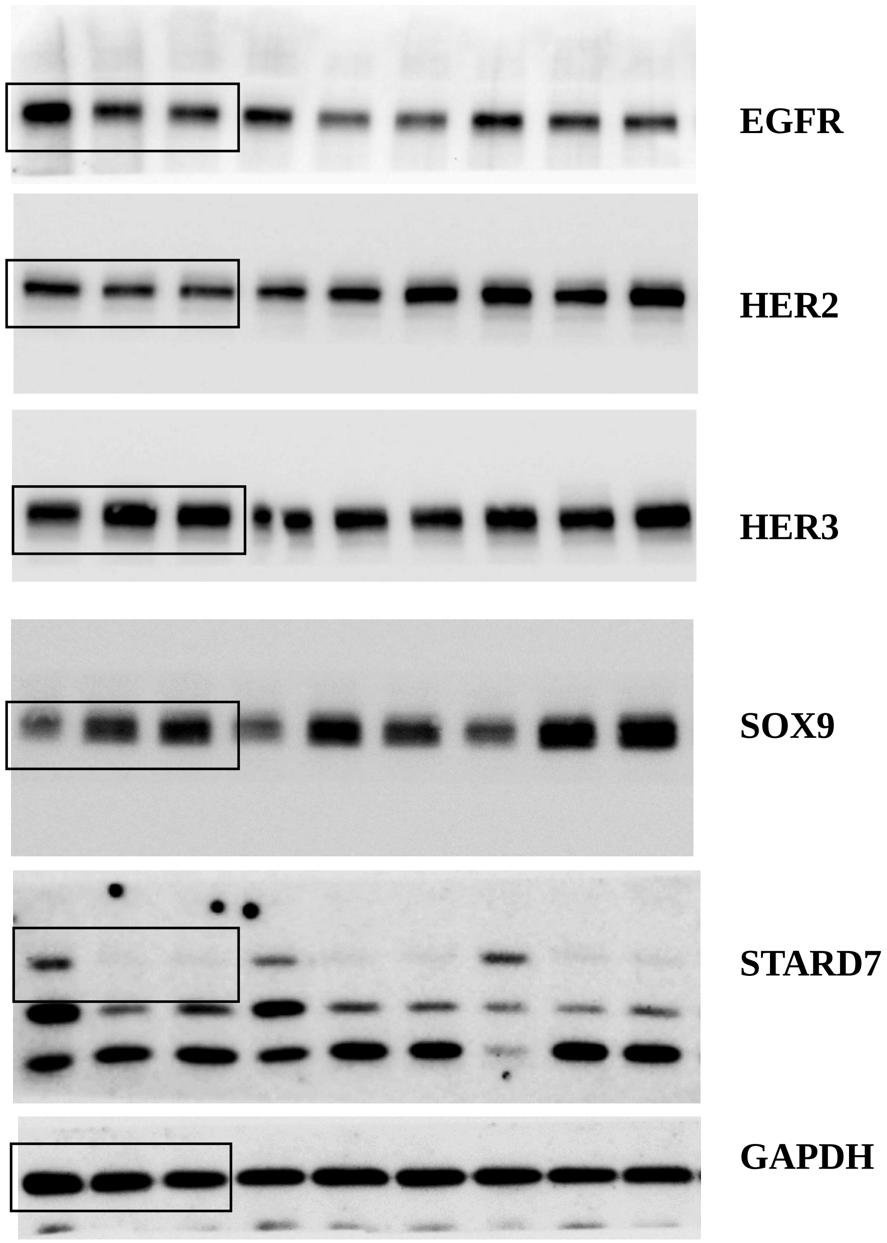

Figure 7A

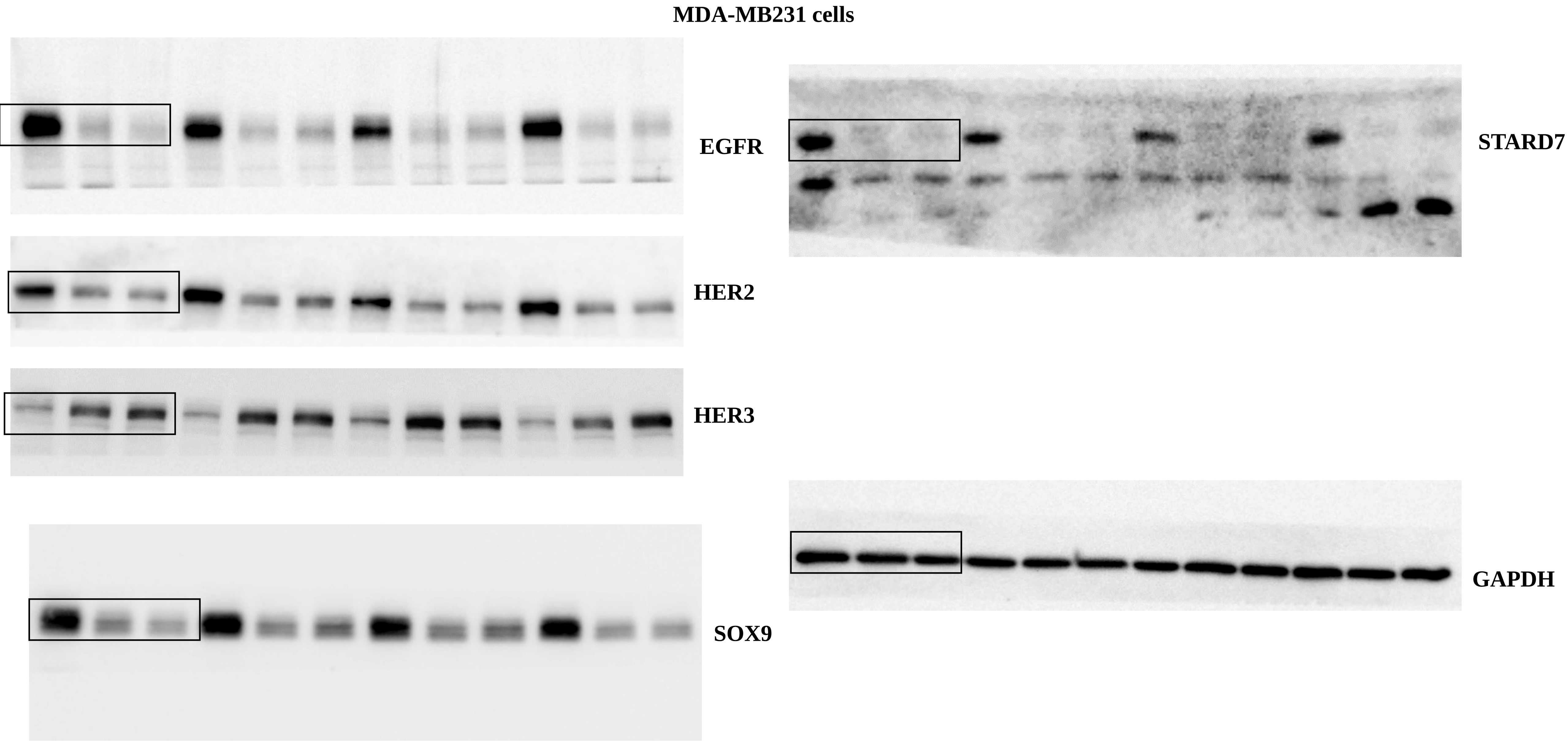

Figure 7B

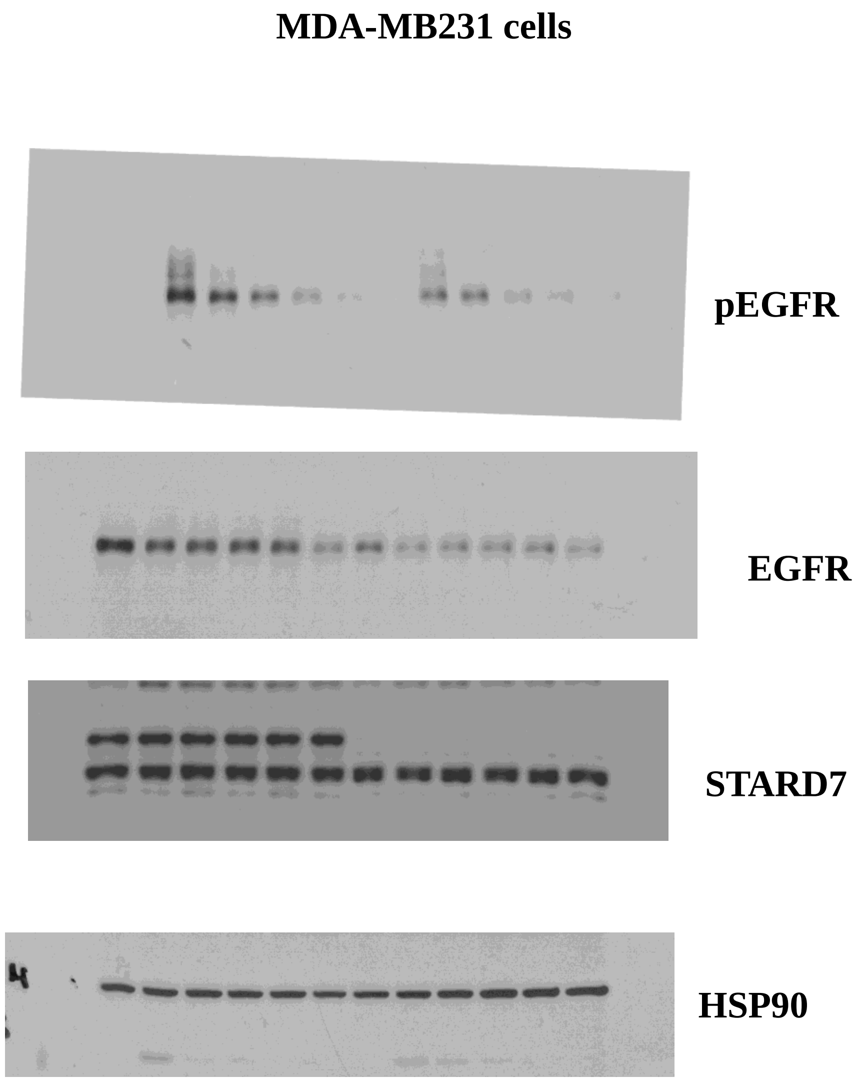
MDA-MB231 cells
pEGFR
EGFR
STARD7
HSP90

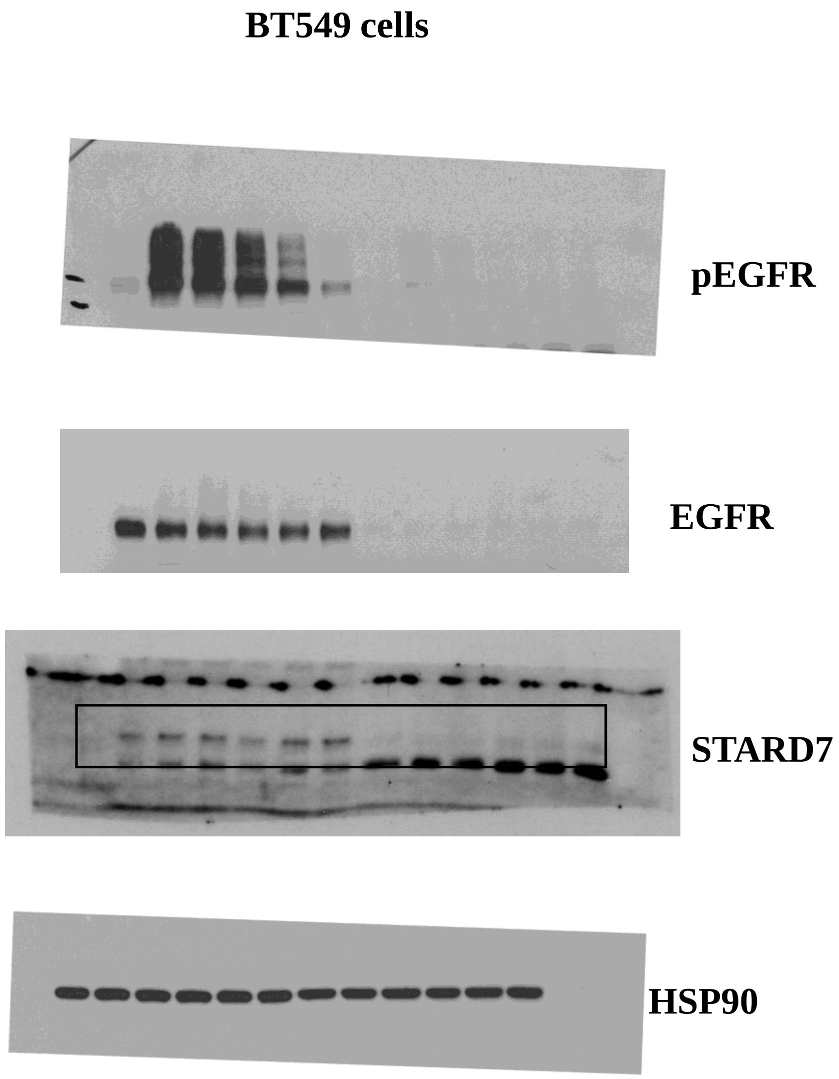
BT549 cells
pEGFR
EGFR
STARD7
HSP90

Figure 7C

MDA-MB231 cells

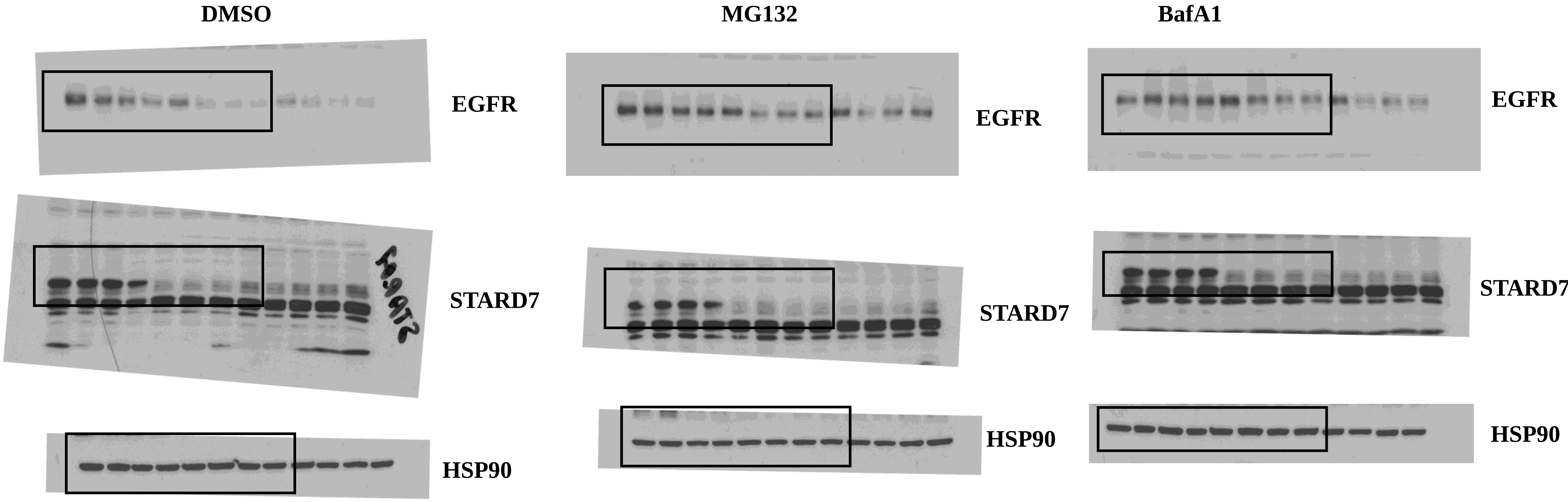

Figure 8A

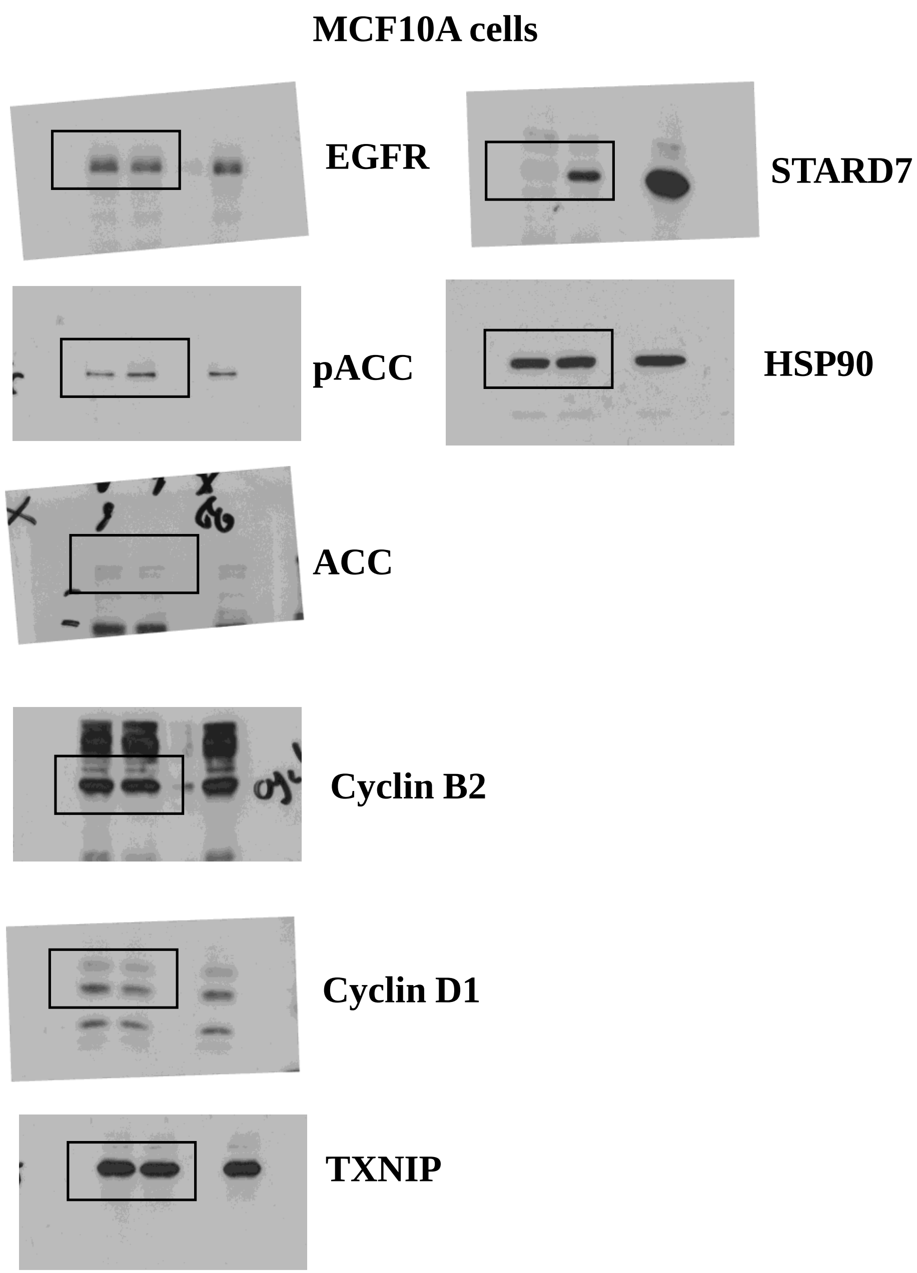

Figure 8B

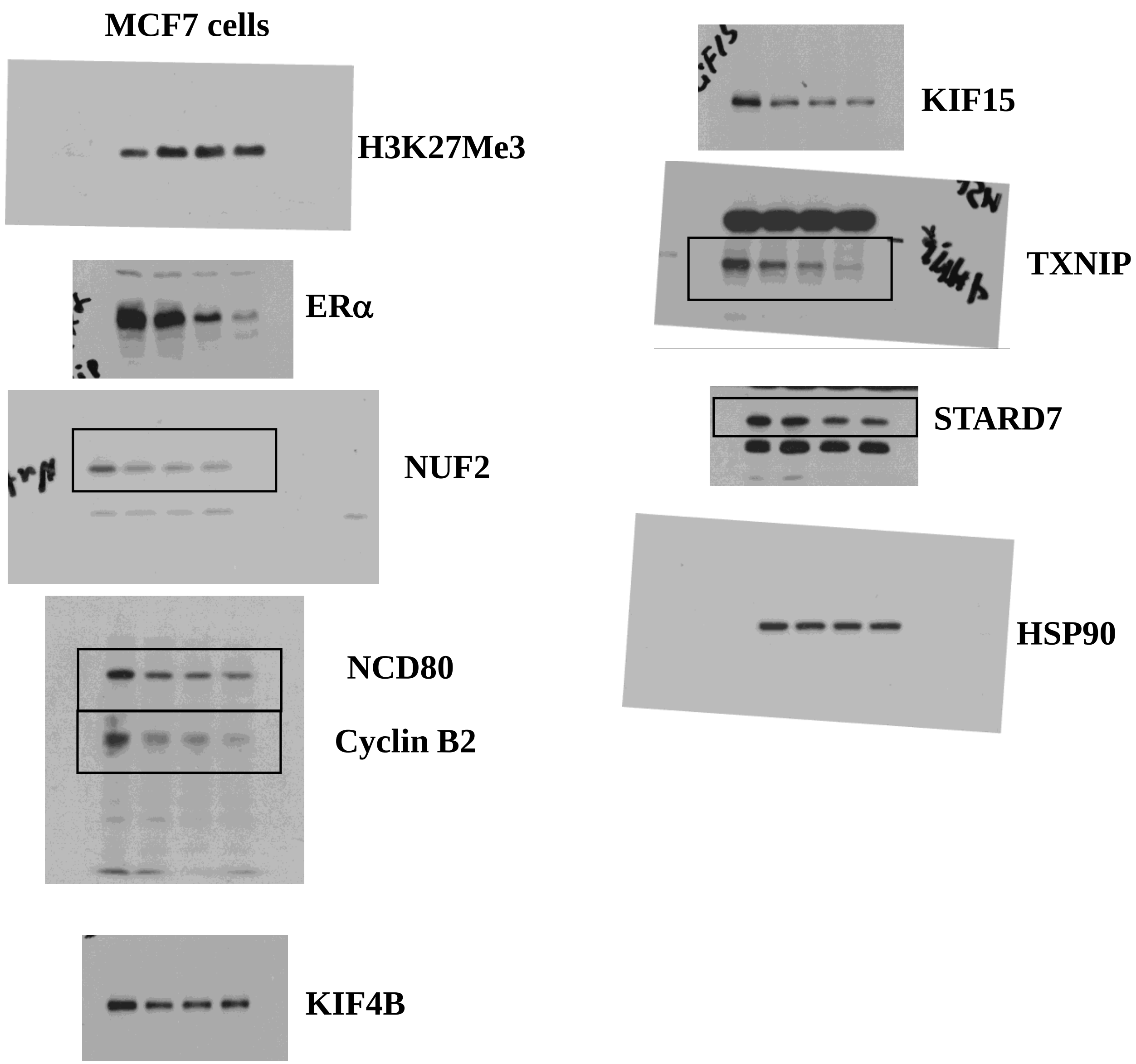

Figure 8B

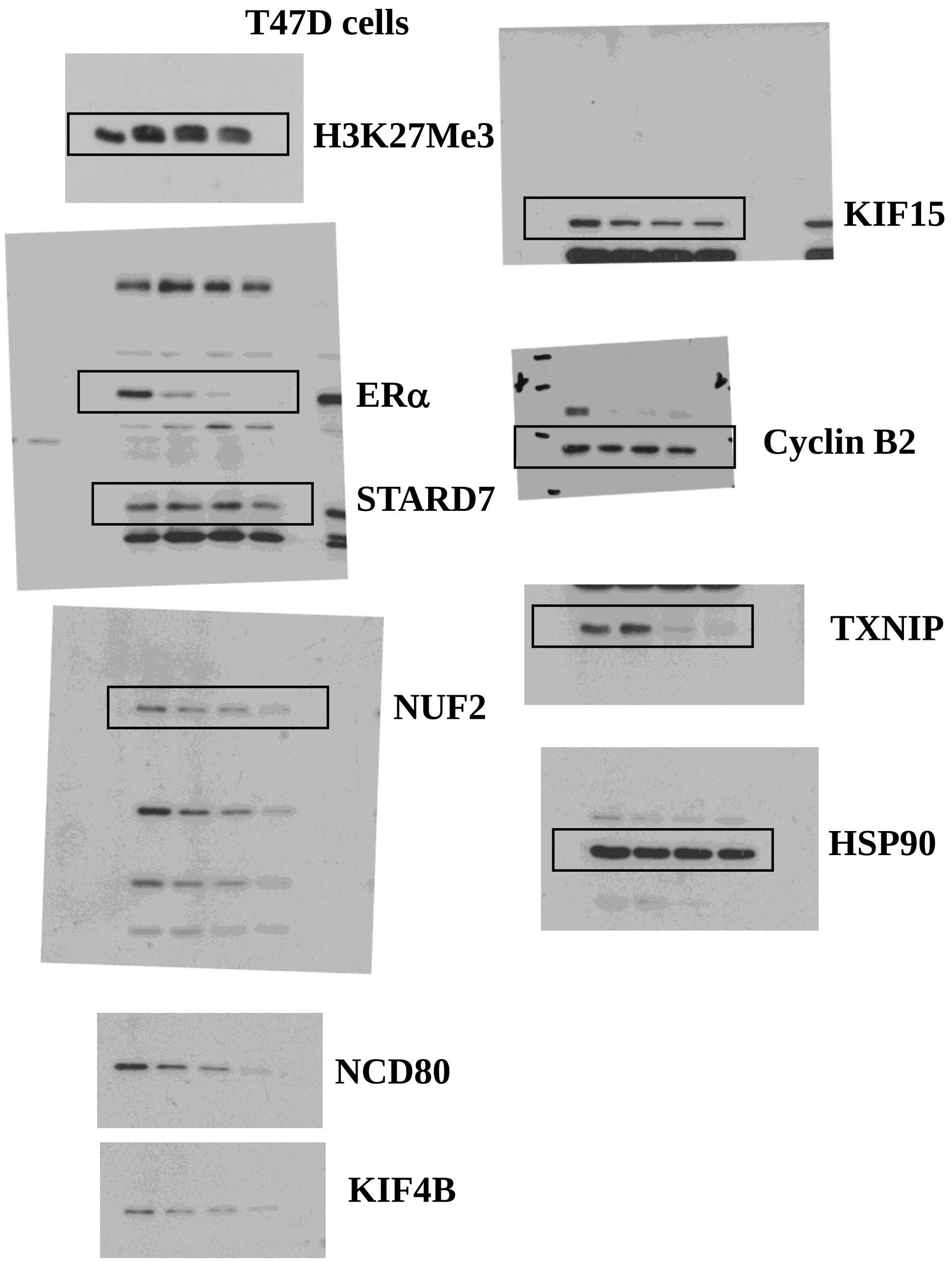
T47D cells
H3K27Me3
ERα
STARD7
NUF2
NCD80
KIF4B
KIF15
Cyclin B2
TXNIP
HSP90

Figure 8C

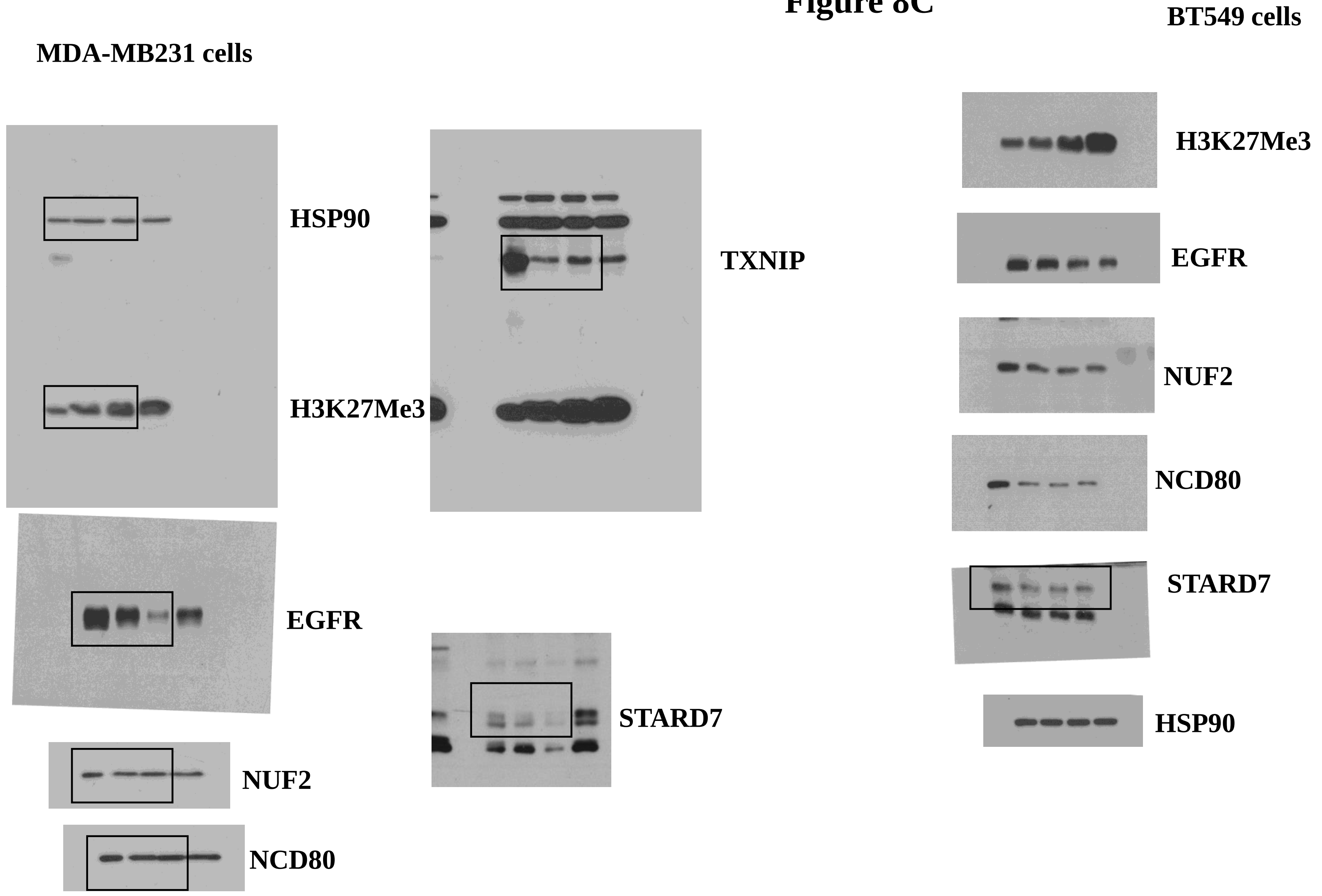

MDA-MB231 cells
HSP90
H3K27Me3
TXNIP
EGFR
STARD7
NUF2
NCD80
BT549 cells
H3K27Me3
EGFR
NUF2
NCD80
STARD7
HSP90

Figure 9D

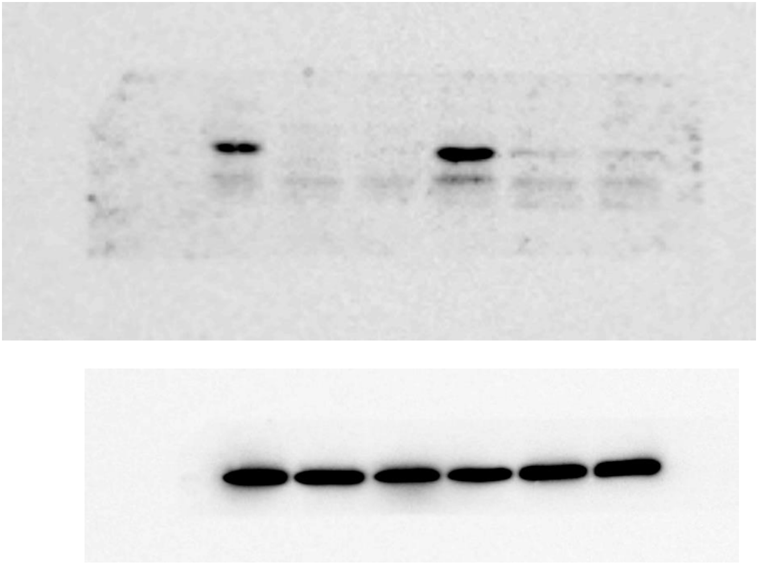

Figure 10B

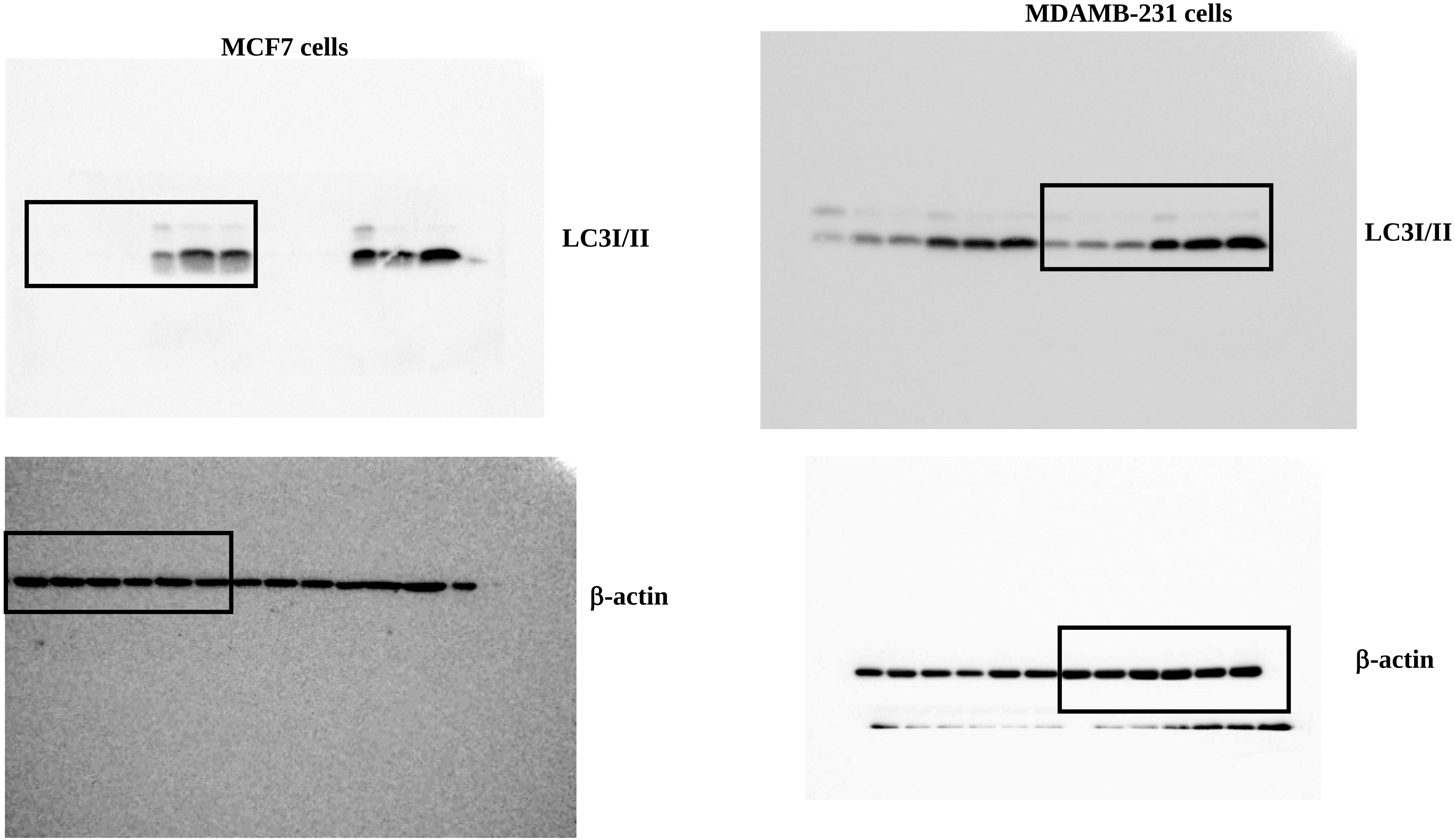

Figure 10C

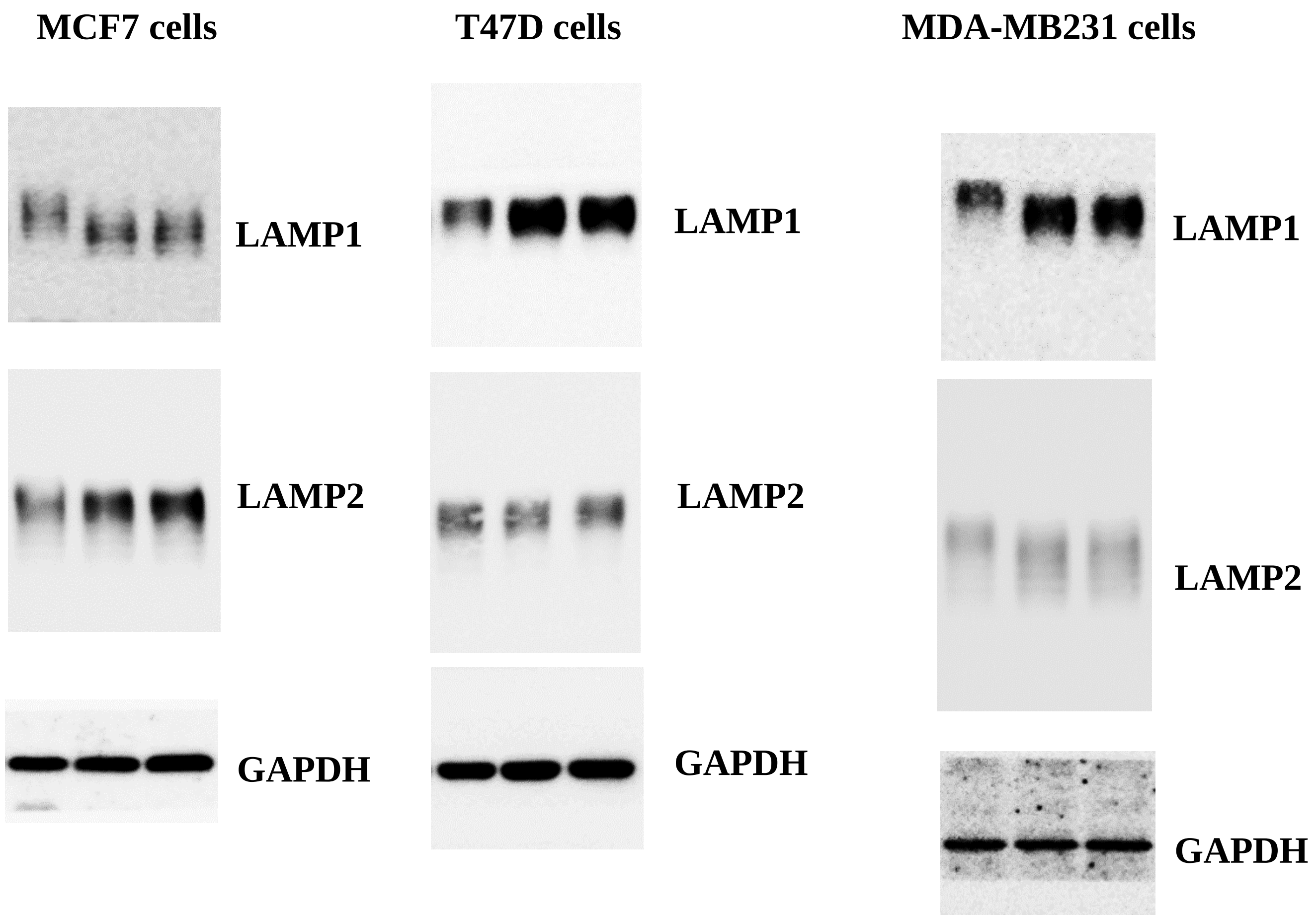
MCF7 cells
T47D cells
MDA-MB231 cells
LAMP1
LAMP2
GAPDH
LAMP1
LAMP2
GAPDH
LAMP1
LAMP2
GAPDH

Supplementary Figure 1C

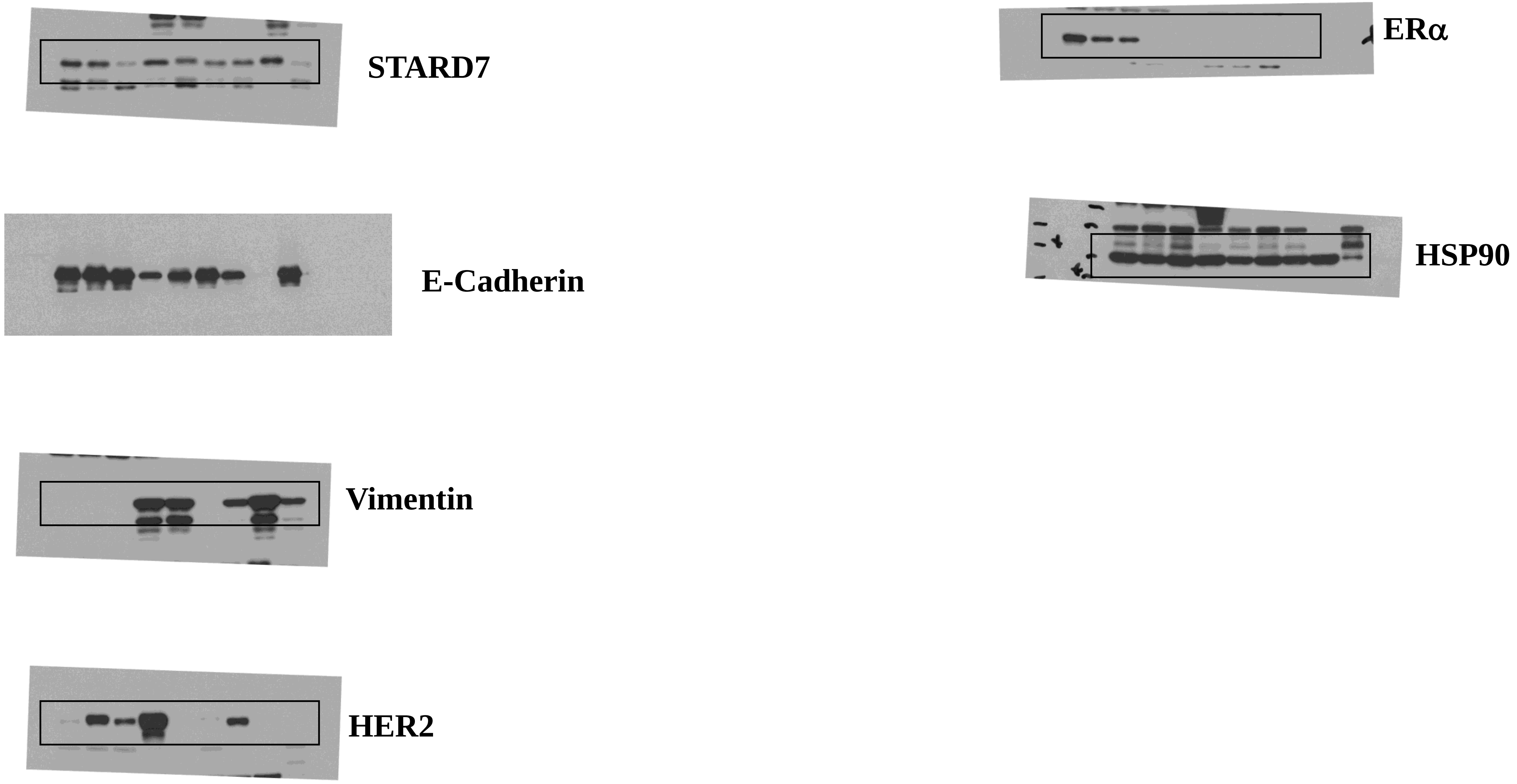

Supplementary Figure 2B

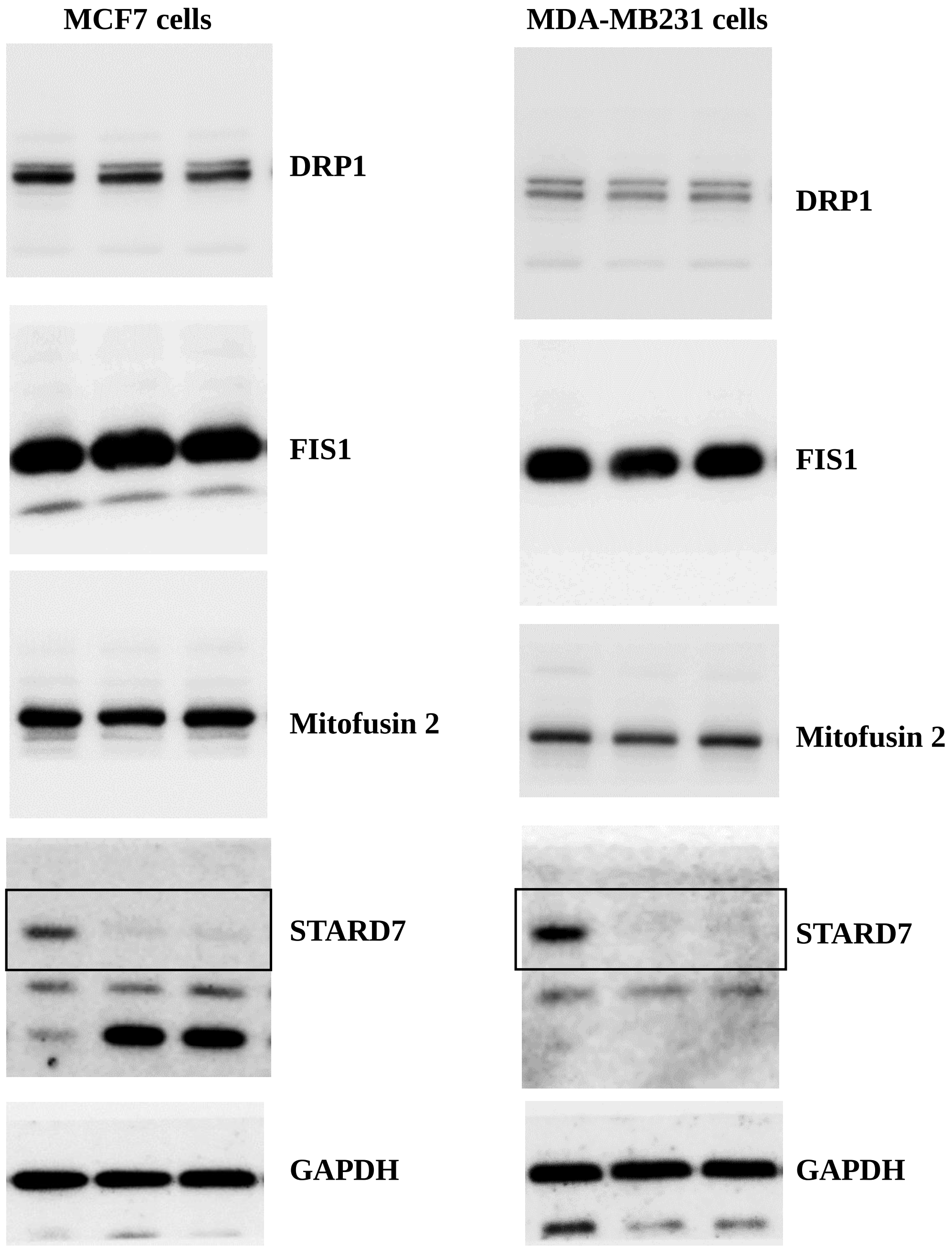

Supplement: Supplementary file 2 — Uncropped gels STARD7 and breast cancer. [file ADVS-12-e03022-s002.pdf]
