# Supplementary material for: Loss of STARD7 Triggers Metabolic Reprogramming and Cell Cycle Arrest in Breast Cancer
Source: Adv Sci (Weinh). 2025 May 30;12(31):e03022. doi: 10.1002/advs.202503022 (PMC12376514; doi:10.1002/advs.202503022)
Supplement: Supplementary file 3 — Supplementary file Densitometry graphs for Figures 1 to 10. [file ADVS-12-e03022-s003.pdf]

ADVANCED SCIENCE
Open Access

Supporting Information

for *Adv. Sci.*, DOI 10.1002/advs.202503022

Loss of STARD7 Triggers Metabolic Reprogramming and Cell Cycle Arrest in Breast Cancer

*Ewelina Dondajewska, Paula Allepuz-Fuster, Chloé Maurizy, Alexandre Hego, Sandra Ormenese, Quentin Lion, Arnaud Blomme, Pierre Close, Arnaud Lavergne, Latifa Karim, Marc Thiry, Ivan Nemazanyy, Roopesh Krishnankutty, Jair Marques Jr, Alex von Kriegsheim, Nathaniel F. Henneman, Ganna Panasyuk, Kateryna Shostak and Alain Chariot**

Fig.1A

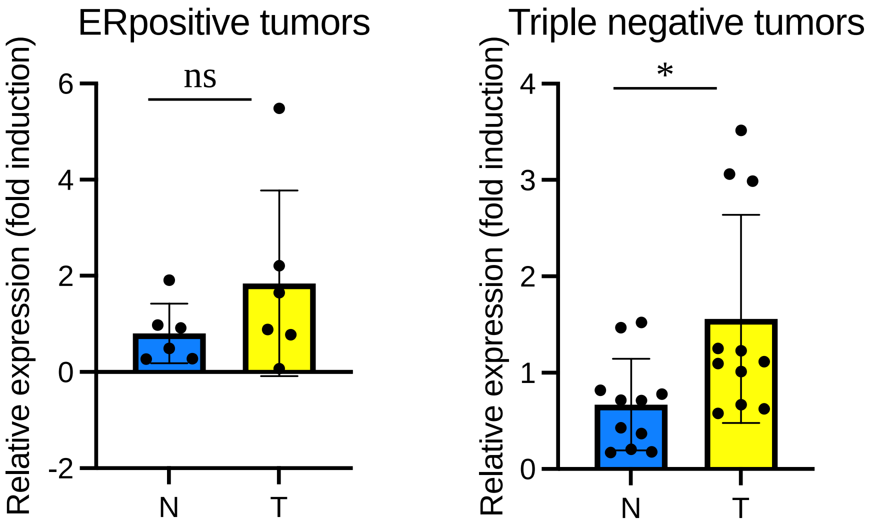
ERpositive tumors
Relative expression (fold induction)
6
4
2
0
-2
ns
N
T
Triple negative tumors
Relative expression (fold induction)
4
3
2
1
0
*
N
T

Fig.2E

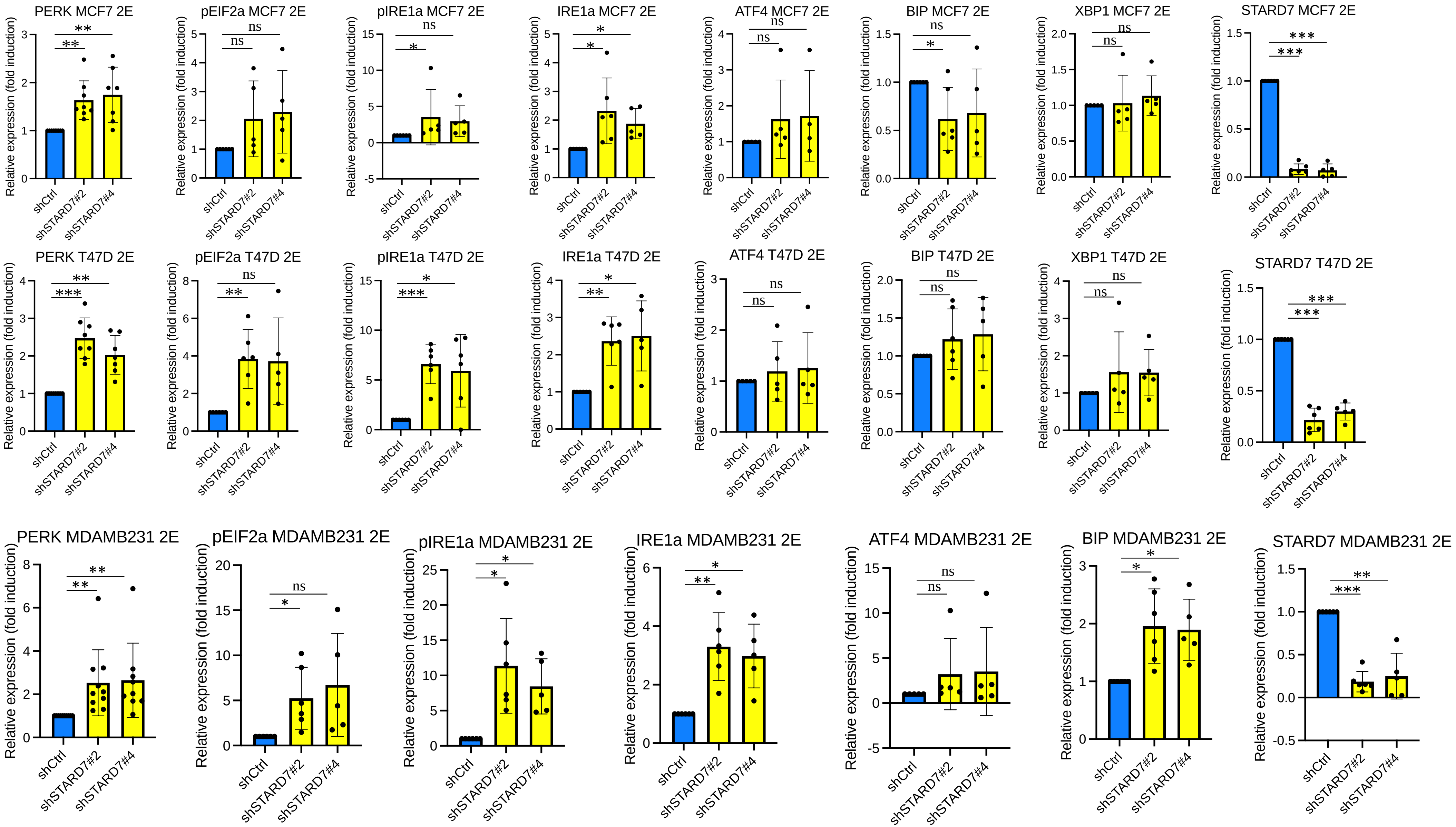
PERK MCF7 2E
pEIF2a MCF7 2E
pIRE1a MCF7 2E
IRE1a MCF7 2E
ATF4 MCF7 2E
BIP MCF7 2E
XBP1 MCF7 2E
STARD7 MCF7 2E
PERK T47D 2E
pEIF2a T47D 2E
pIRE1a T47D 2E
IRE1a T47D 2E
ATF4 T47D 2E
BIP T47D 2E
XBP1 T47D 2E
STARD7 T47D 2E
PERK MDAMB231 2E
pEIF2a MDAMB231 2E
pIRE1a MDAMB231 2E
IRE1a MDAMB231 2E
ATF4 MDAMB231 2E
BIP MDAMB231 2E
STARD7 MDAMB231 2E
Relative expression (fold induction)
shCtrl
shSTARD7#2
shSTARD7#4
ns
*
**
***

Fig.2D

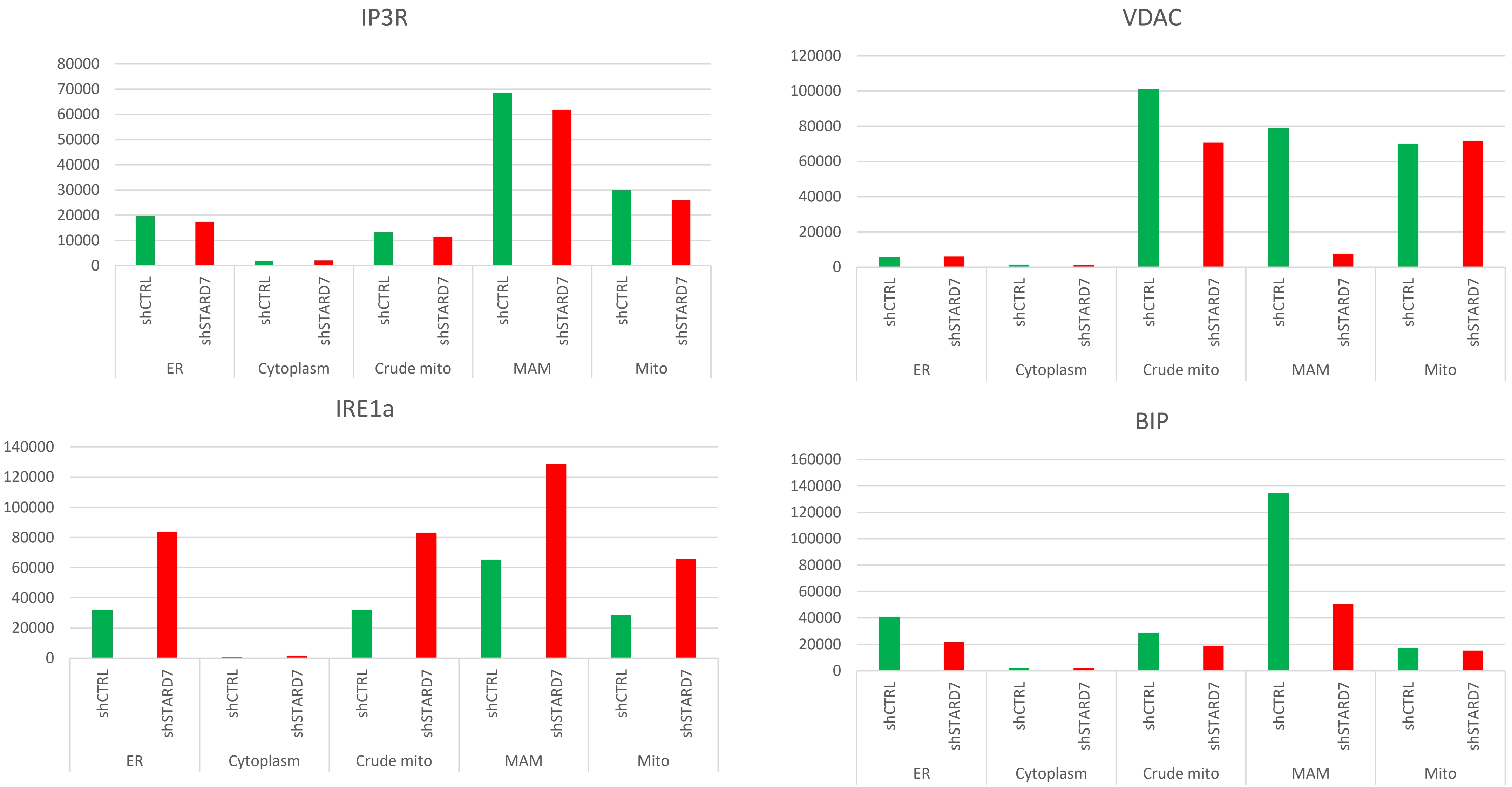
IP3R
80000
70000
60000
50000
40000
30000
20000
10000
0
shCTRL
shSTARD7
ER
Cytoplasm
Crude mito
MAM
Mito
VDAC
120000
100000
80000
60000
40000
20000
0
IRE1a
140000
120000
100000
80000
60000
40000
20000
0
BIP
160000
140000
120000
100000
80000
60000
40000
20000
0

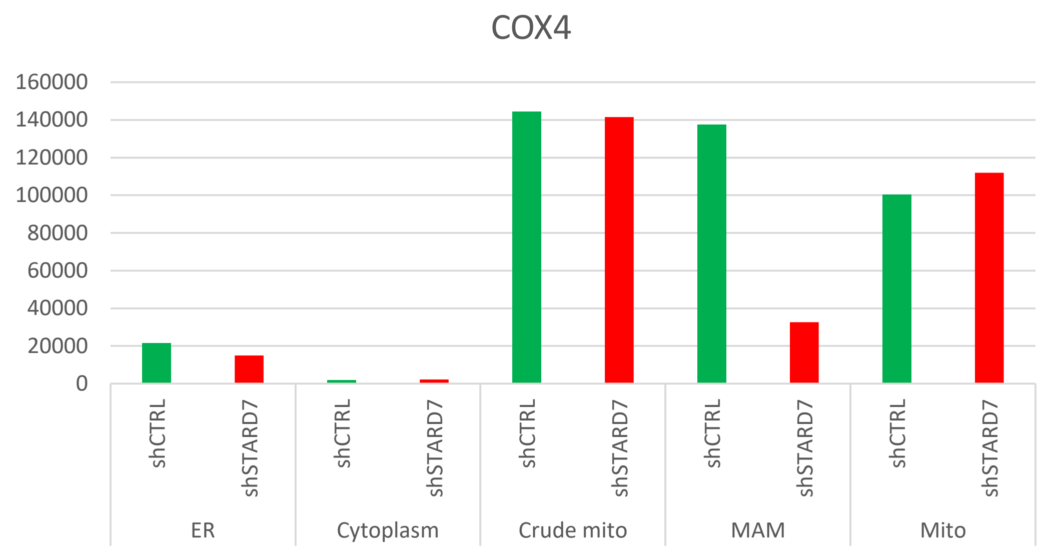
COX4
160000
140000
120000
100000
80000
60000
40000
20000
0
shCTRL
shSTARD7
ER
Cytoplasm
Crude mito
MAM
Mito

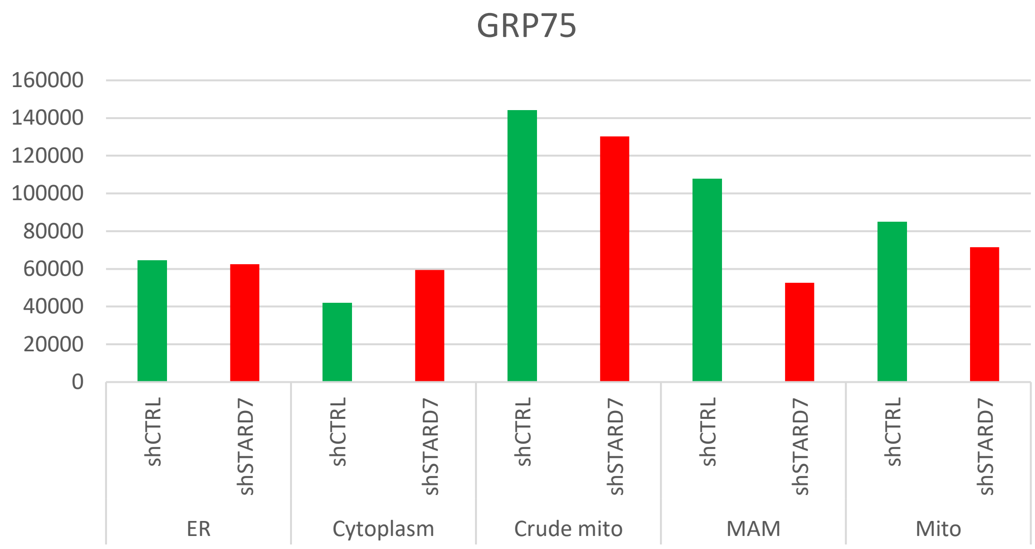
GRP75
160000
140000
120000
100000
80000
60000
40000
20000
0
shCTRL
shSTARD7
ER
Cytoplasm
Crude mito
MAM
Mito

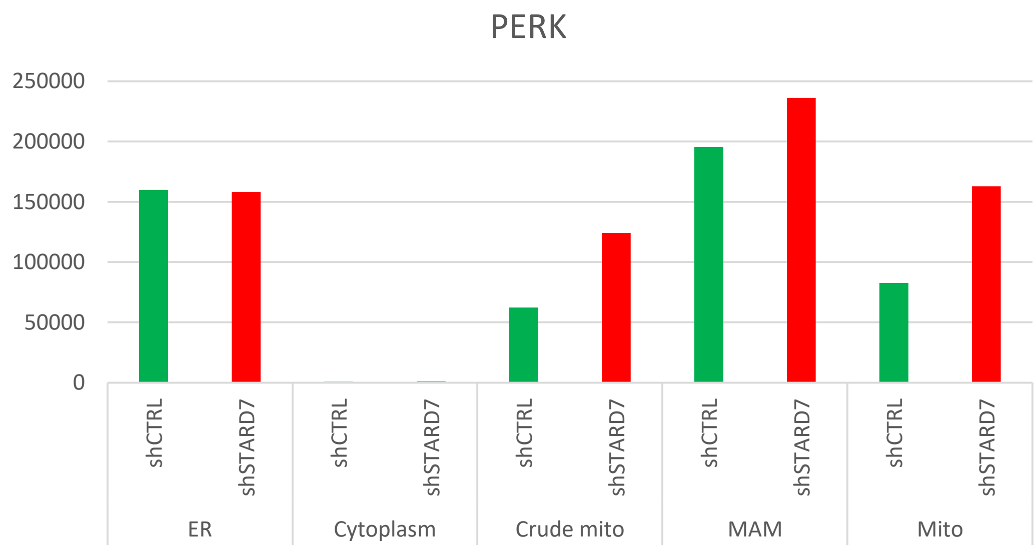
PERK
250000
200000
150000
100000
50000
0
shCTRL
shSTARD7
ER
Cytoplasm
Crude mito
MAM
Mito

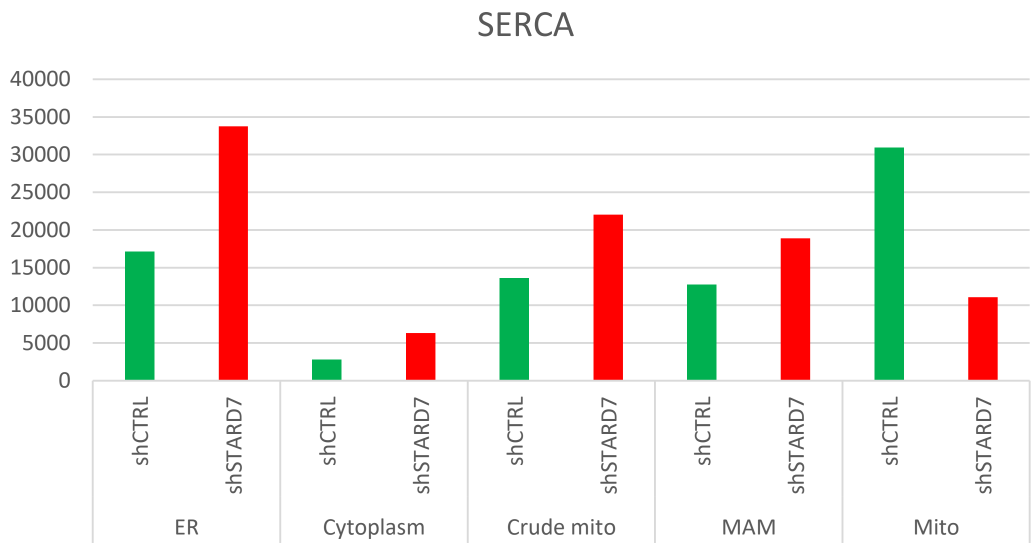
SERCA
40000
35000
30000
25000
20000
15000
10000
5000
0
shCTRL
shSTARD7
ER
Cytoplasm
Crude mito
MAM
Mito

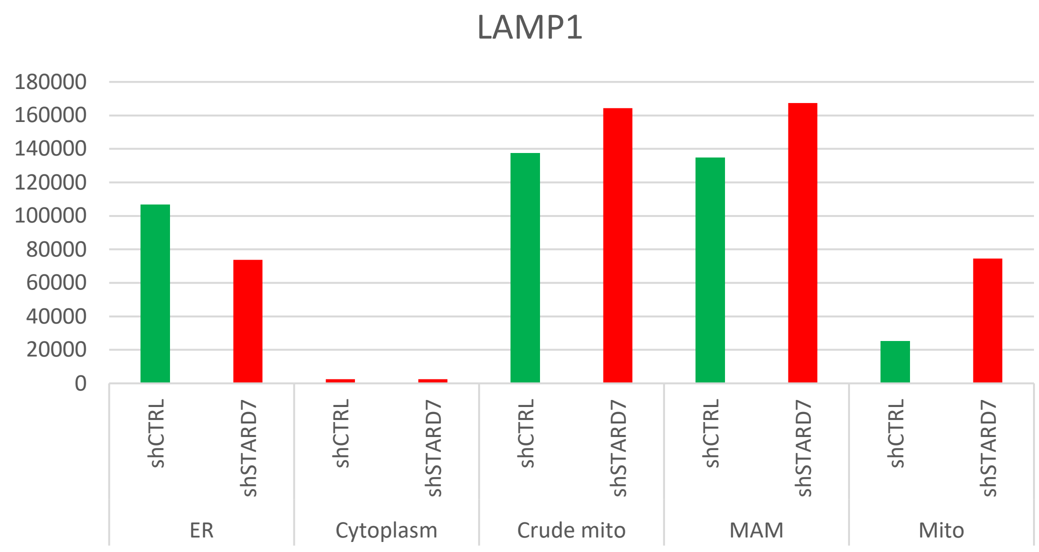
LAMP1
180000
160000
140000
120000
100000
80000
60000
40000
20000
0
shCTRL
shSTARD7
ER
Cytoplasm
Crude mito
MAM
Mito

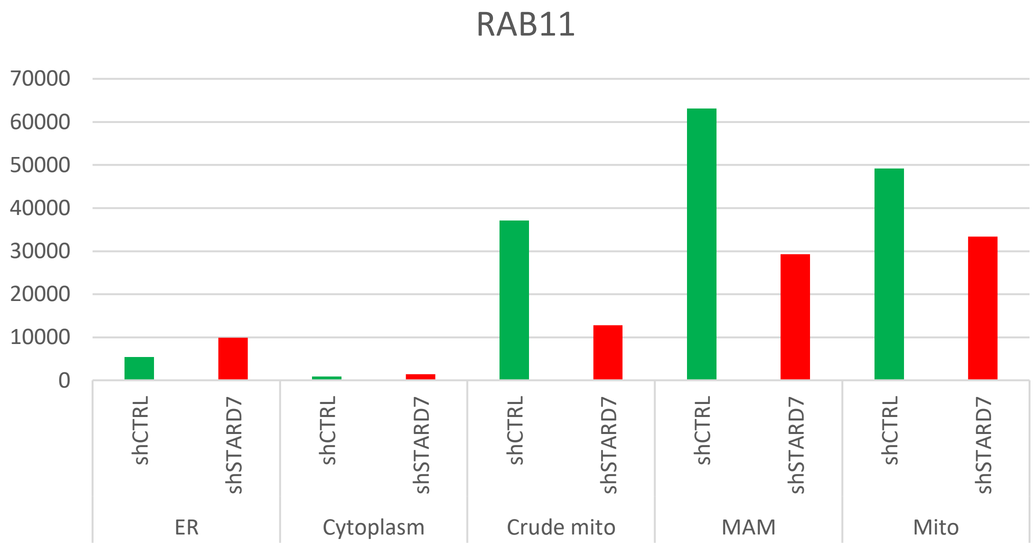
RAB11
70000
60000
50000
40000
30000
20000
10000
0
shCTRL
shSTARD7
ER
Cytoplasm
Crude mito
MAM
Mito

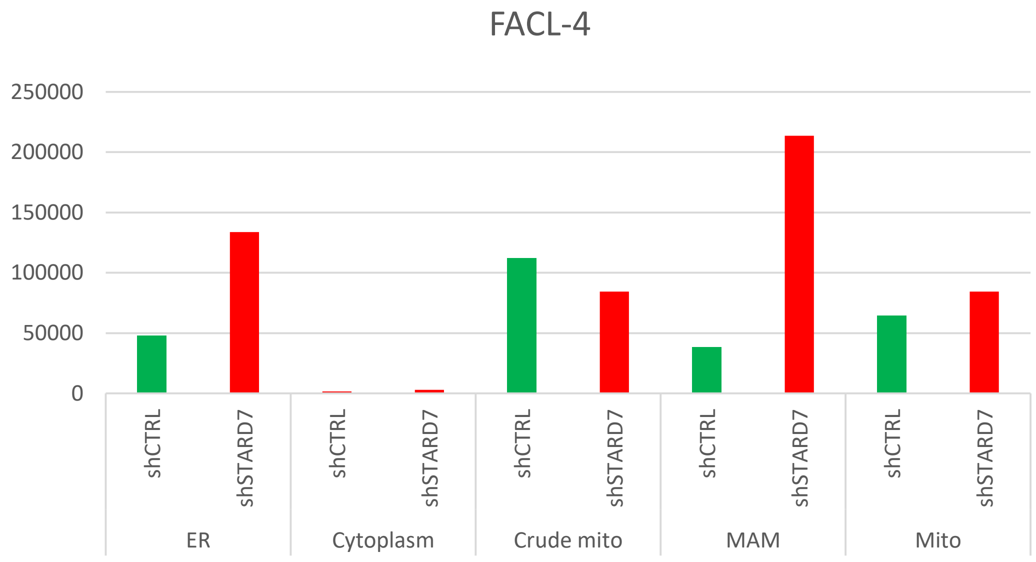
FACL-4
250000
200000
150000
100000
50000
0
shCTRL
shSTARD7
ER
Cytoplasm
Crude mito
MAM
Mito

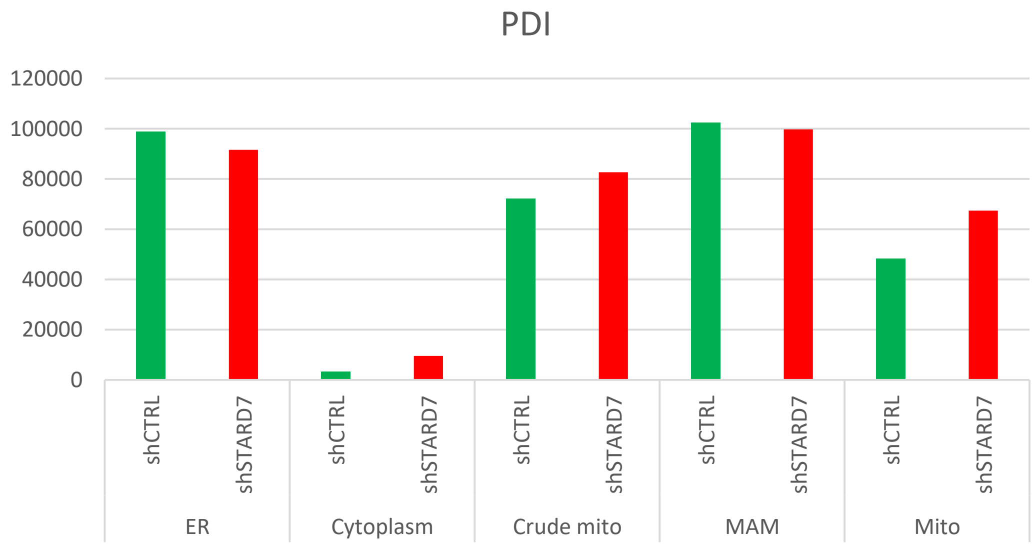
PDI
120000
100000
80000
60000
40000
20000
0
shCTRL
shSTARD7
ER
Cytoplasm
Crude mito
MAM
Mito

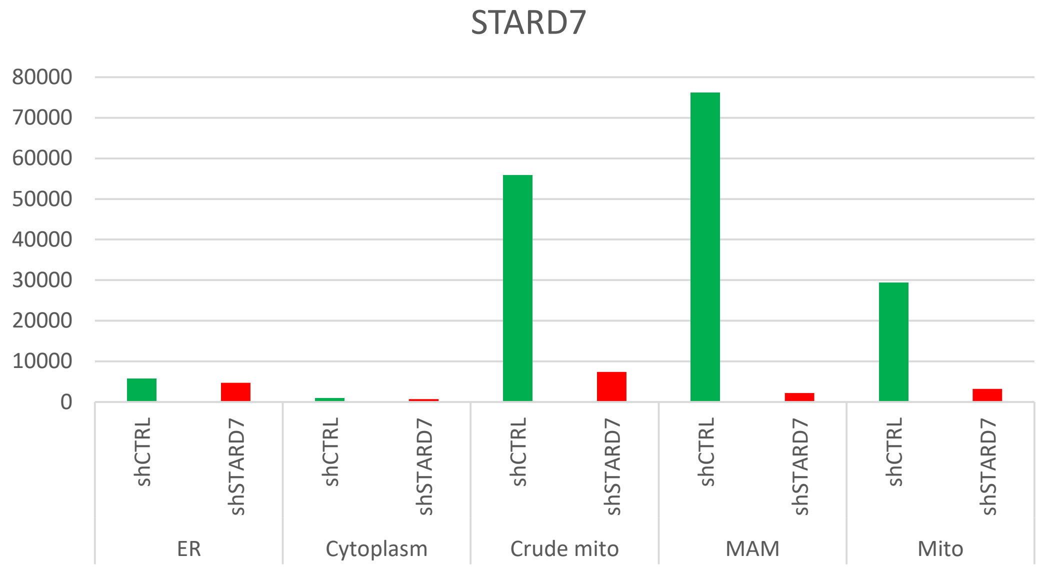
STARD7
80000
70000
60000
50000
40000
30000
20000
10000
0
shCTRL
shSTARD7
ER
Cytoplasm
Crude mito
MAM
Mito

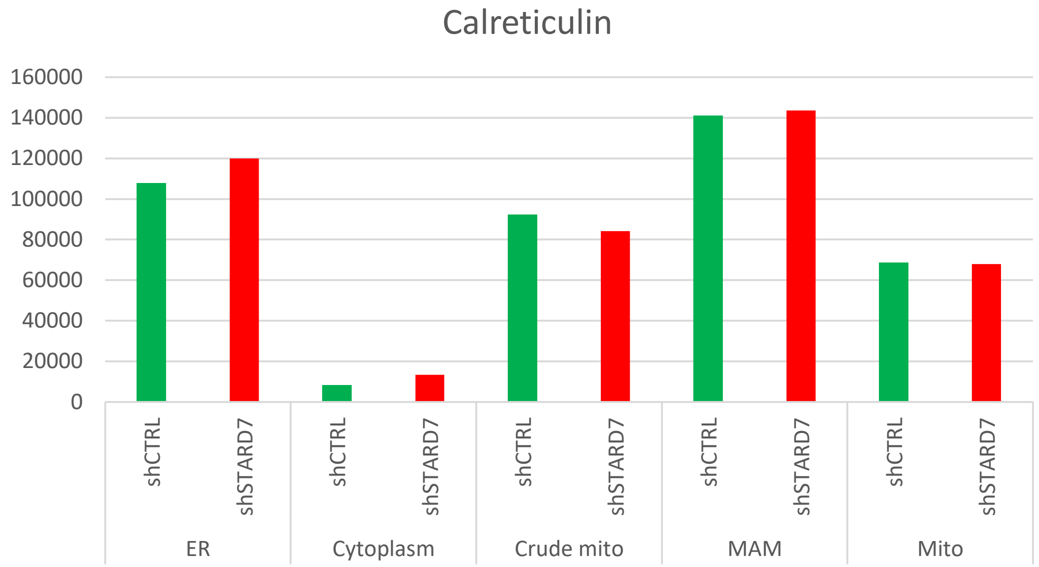
Calreticulin
160000
140000
120000
100000
80000
60000
40000
20000
0
shCTRL
shSTARD7
ER
Cytoplasm
Crude mito
MAM
Mito

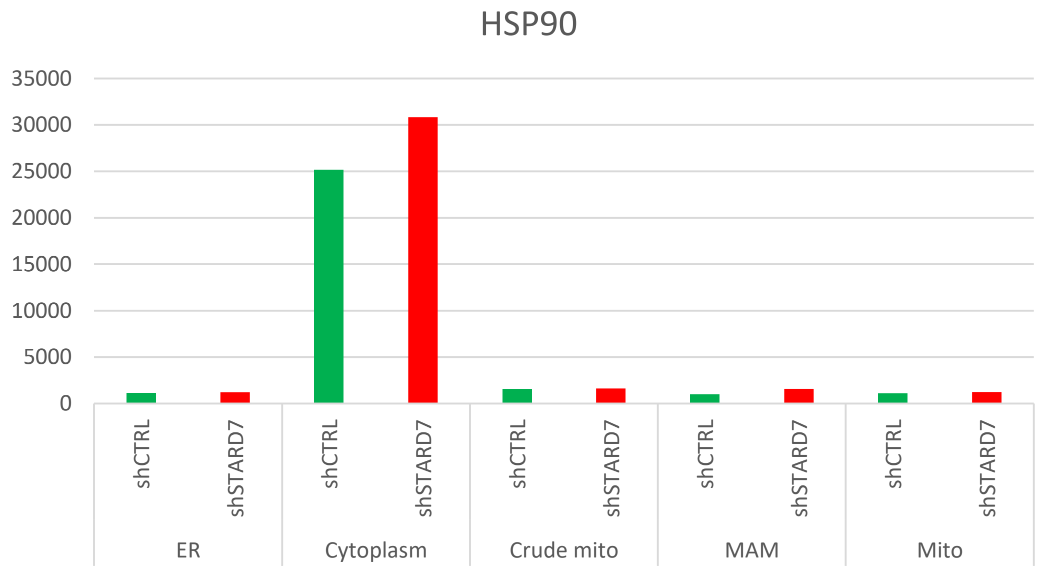
HSP90
35000
30000
25000
20000
15000
10000
5000
0
shCTRL
shSTARD7
ER
Cytoplasm
Crude mito
MAM
Mito

Fig.3C

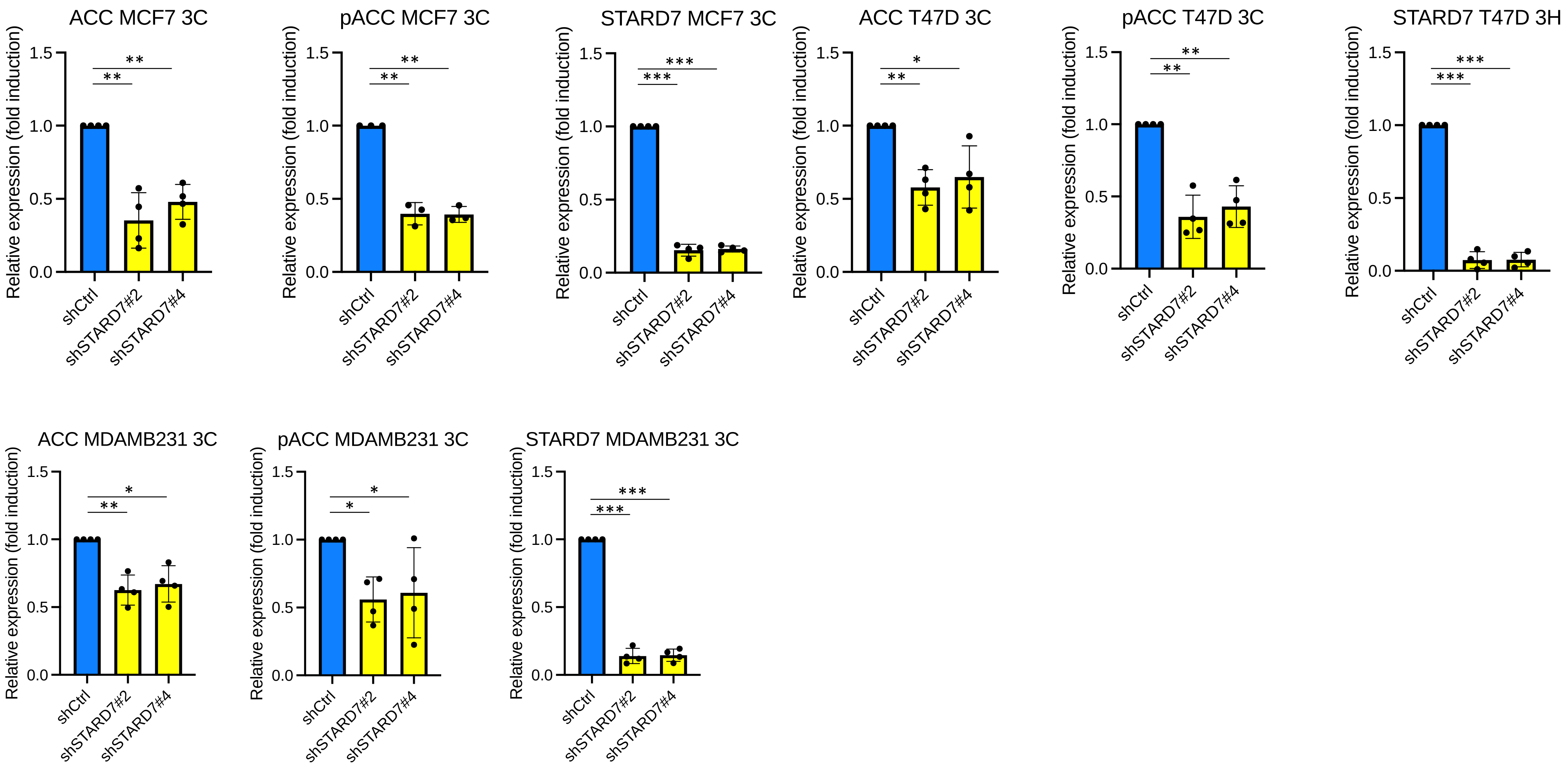

ACC MCF7 3C
pACC MCF7 3C
STARD7 MCF7 3C
ACC T47D 3C
pACC T47D 3C
STARD7 T47D 3H
ACC MDAMB231 3C
pACC MDAMB231 3C
STARD7 MDAMB231 3C
Relative expression (fold induction)
0.0
0.5
1.0
1.5
shCtrl
shSTARD7#2
shSTARD7#4
*
**
***

Fig.3H

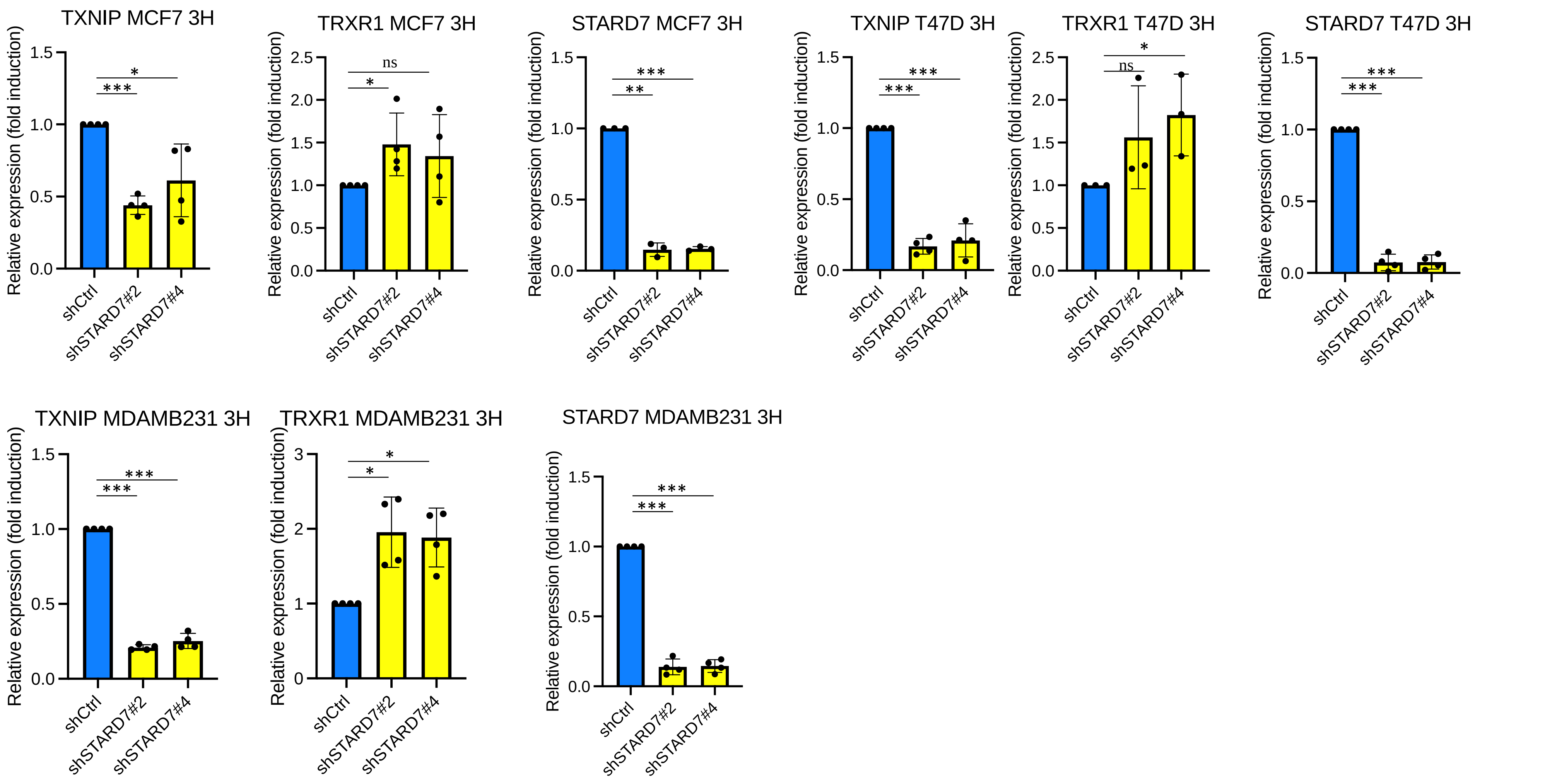

TXNIP MCF7 3H
TRXR1 MCF7 3H
STARD7 MCF7 3H
TXNIP T47D 3H
TRXR1 T47D 3H
STARD7 T47D 3H
TXNIP MDAMB231 3H
TRXR1 MDAMB231 3H
STARD7 MDAMB231 3H
Relative expression (fold induction)
shCtrl
shSTARD7#2
shSTARD7#4
ns
*
**
***

Fig.4E

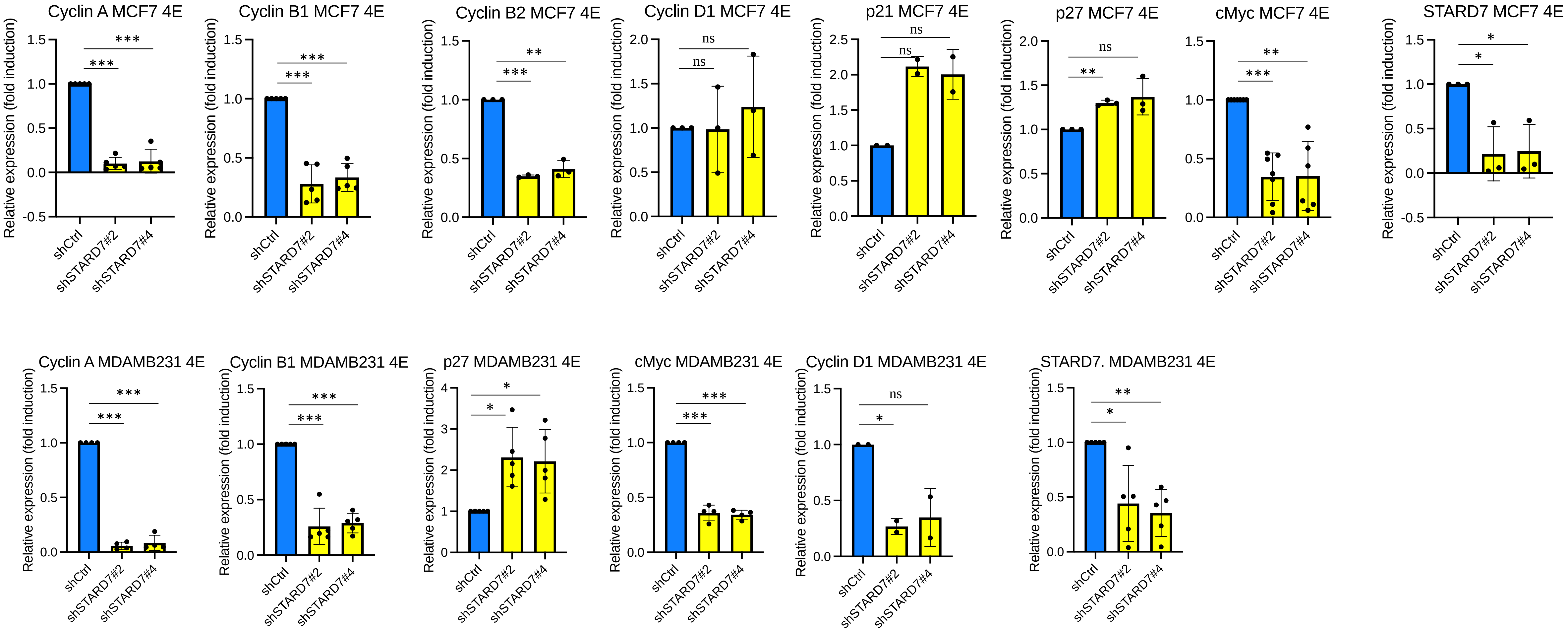
Cyclin A MCF7 4E
Cyclin B1 MCF7 4E
Cyclin B2 MCF7 4E
Cyclin D1 MCF7 4E
p21 MCF7 4E
p27 MCF7 4E
cMyc MCF7 4E
STARD7 MCF7 4E
Cyclin A MDAMB231 4E
Cyclin B1 MDAMB231 4E
p27 MDAMB231 4E
cMyc MDAMB231 4E
Cyclin D1 MDAMB231 4E
STARD7. MDAMB231 4E
Relative expression (fold induction)
shCtrl
shSTARD7#2
shSTARD7#4
ns
*
**
***

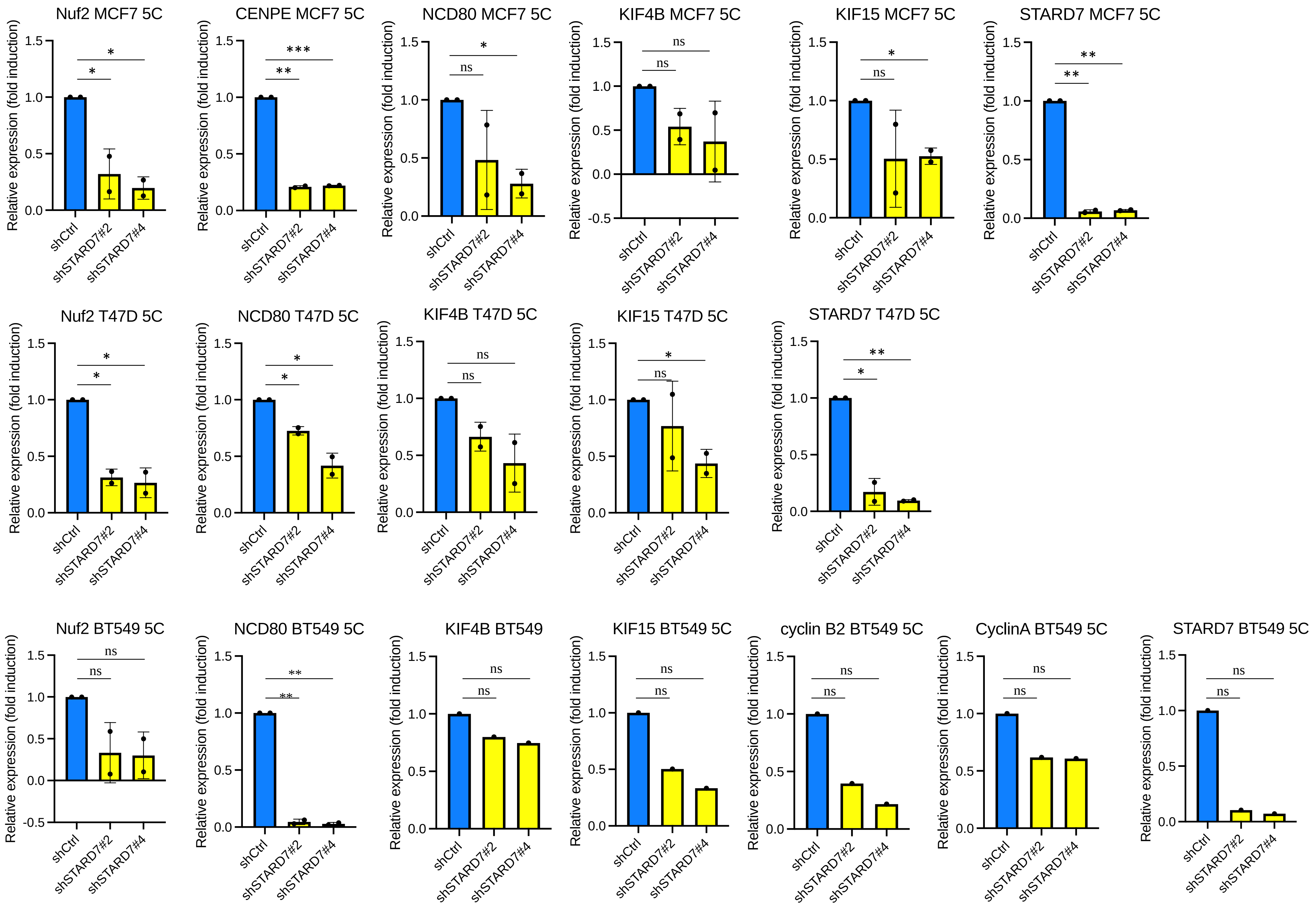
Nuf2 MCF7 5C
CENPE MCF7 5C
NCD80 MCF7 5C
KIF4B MCF7 5C
KIF15 MCF7 5C
STARD7 MCF7 5C
Nuf2 T47D 5C
NCD80 T47D 5C
KIF4B T47D 5C
KIF15 T47D 5C
STARD7 T47D 5C
Nuf2 BT549 5C
NCD80 BT549 5C
KIF4B BT549
KIF15 BT549 5C
cyclin B2 BT549 5C
CyclinA BT549 5C
STARD7 BT549 5C
Relative expression (fold induction)
shCtrl
shSTARD7#2
shSTARD7#4
ns
*
**
***

Fig.5C

Fig.6A

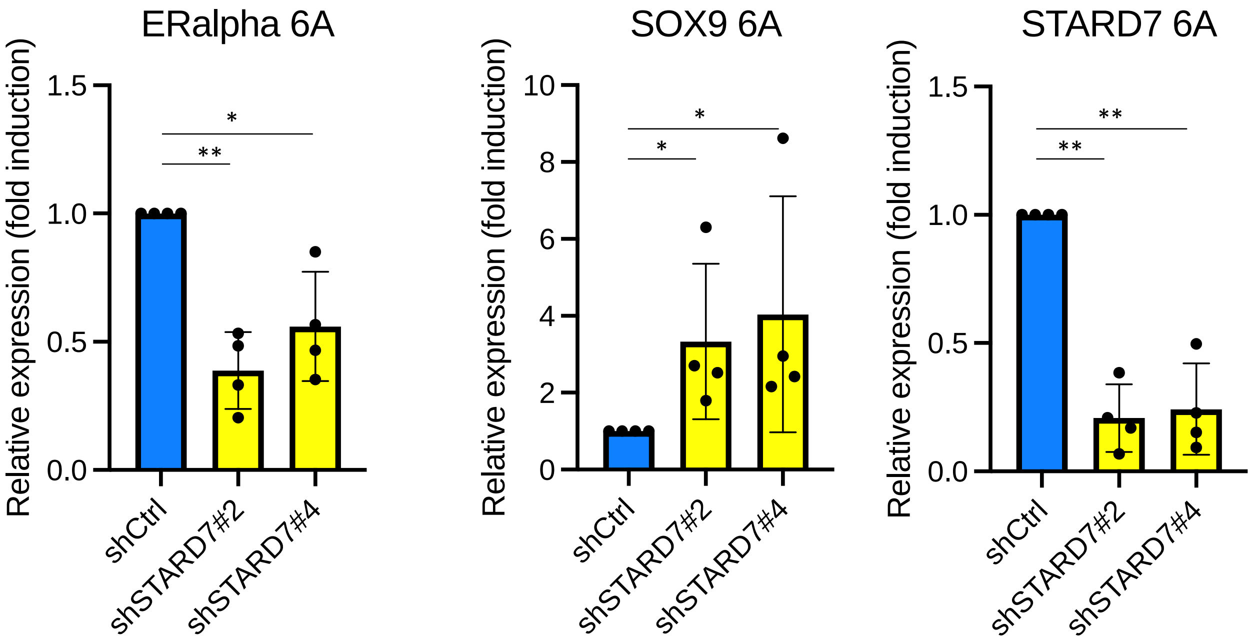

ERalpha 6A
SOX9 6A
STARD7 6A
Relative expression (fold induction)
1.5
1.0
0.5
0.0
10
8
6
4
2
0
*
**
shCtrl
shSTARD7#2
shSTARD7#4

Fig.6B

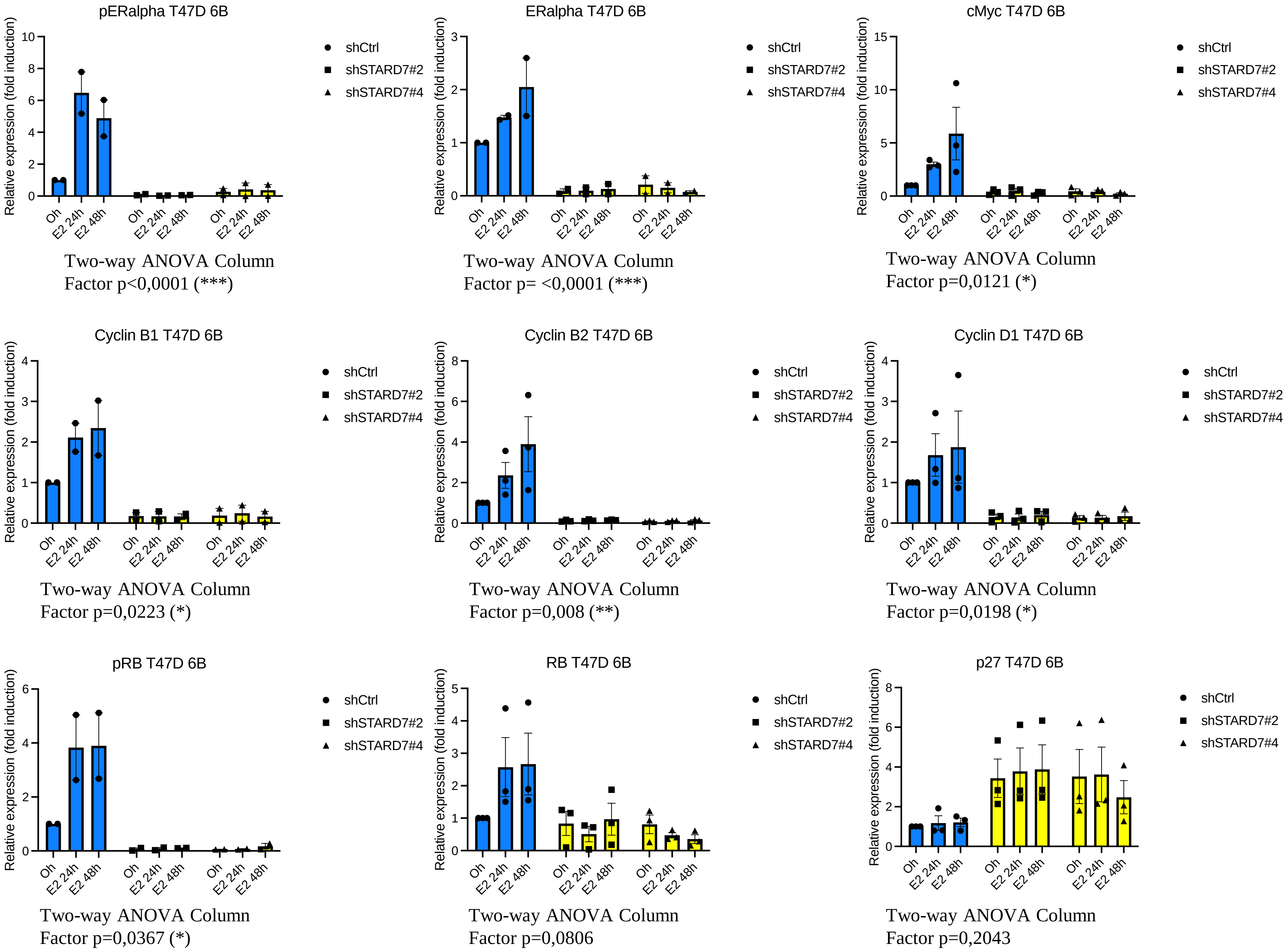
pERalpha T47D 6B
Two-way ANOVA Column Factor p<0,0001 (***)
ERalpha T47D 6B
Two-way ANOVA Column Factor p= <0,0001 (***)
cMyc T47D 6B
Two-way ANOVA Column Factor p=0,0121 (*)
Cyclin B1 T47D 6B
Two-way ANOVA Column Factor p=0,0223 (*)
Cyclin B2 T47D 6B
Two-way ANOVA Column Factor p=0,008 (**)
Cyclin D1 T47D 6B
Two-way ANOVA Column Factor p=0,0198 (*)
pRB T47D 6B
Two-way ANOVA Column Factor p=0,0367 (*)
RB T47D 6B
Two-way ANOVA Column Factor p=0,0806
p27 T47D 6B
Two-way ANOVA Column Factor p=0,2043
Relative expression (fold induction)
Oh
E2 24h
E2 48h
shCtrl
shSTARD7#2
shSTARD7#4

Fig.6B

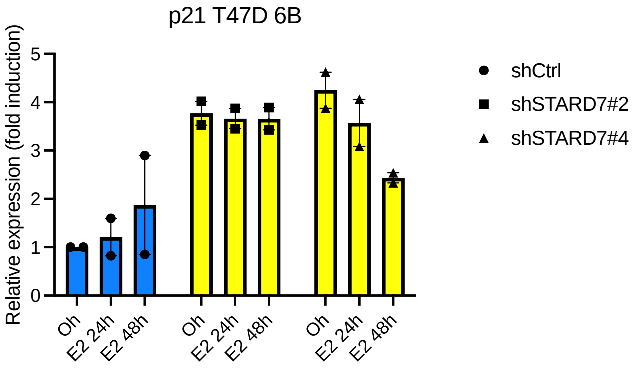

Two-way ANOVA Column Factor p=0,0002 (***)

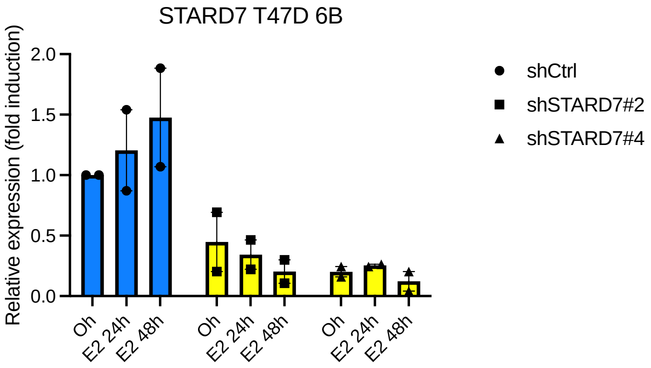

Two-way ANOVA Column Factor p= 0,008 (**)

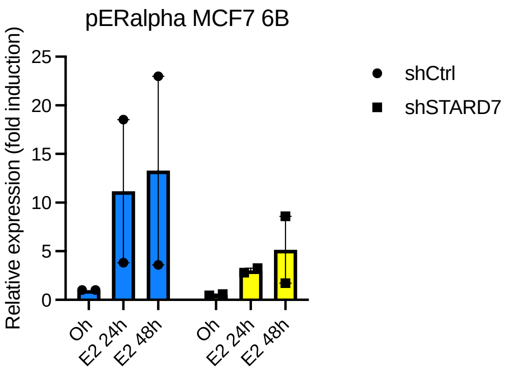

Two-way ANOVA Column Factor p=0,438

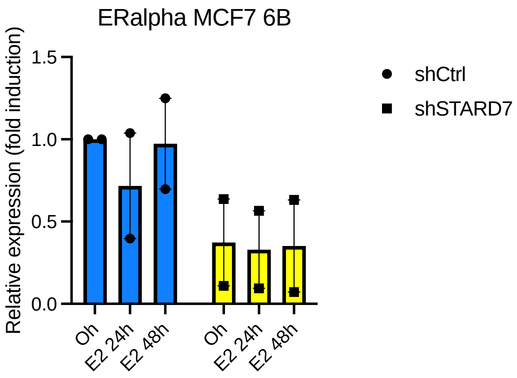

Two-way ANOVA Subject Factor p=0,0108 (*)

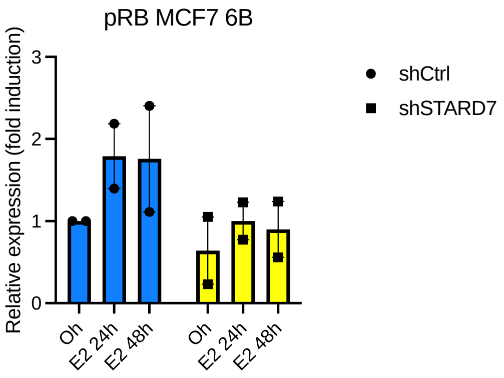

Two-way ANOVA Column Factor p=0,2944

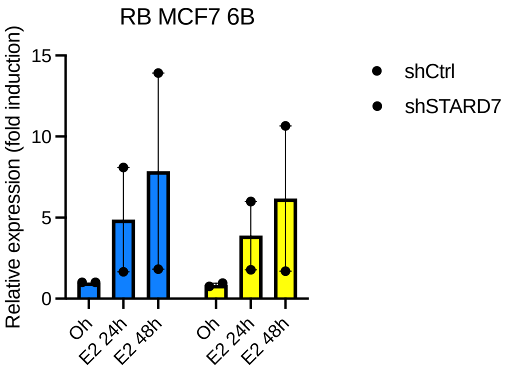

Two-way ANOVA Column Factor p=0,8282

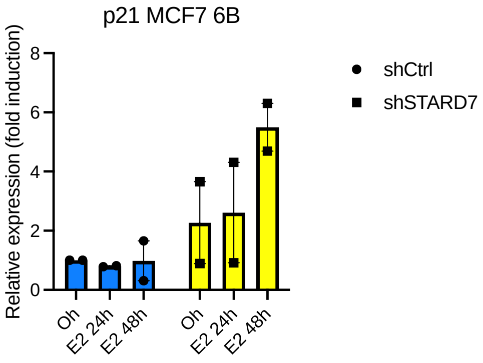

Two-way ANOVA TimeXColumn Factor p=0,0373(*)

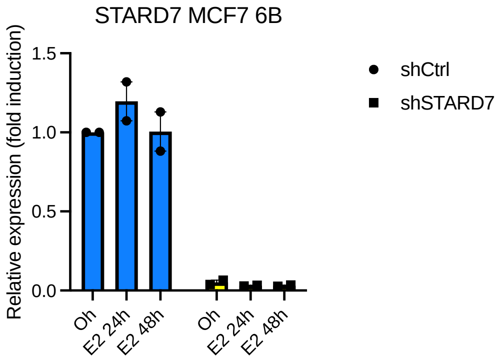

Two-way ANOVA Column Factor p=0,0063(**)

Fig.7A

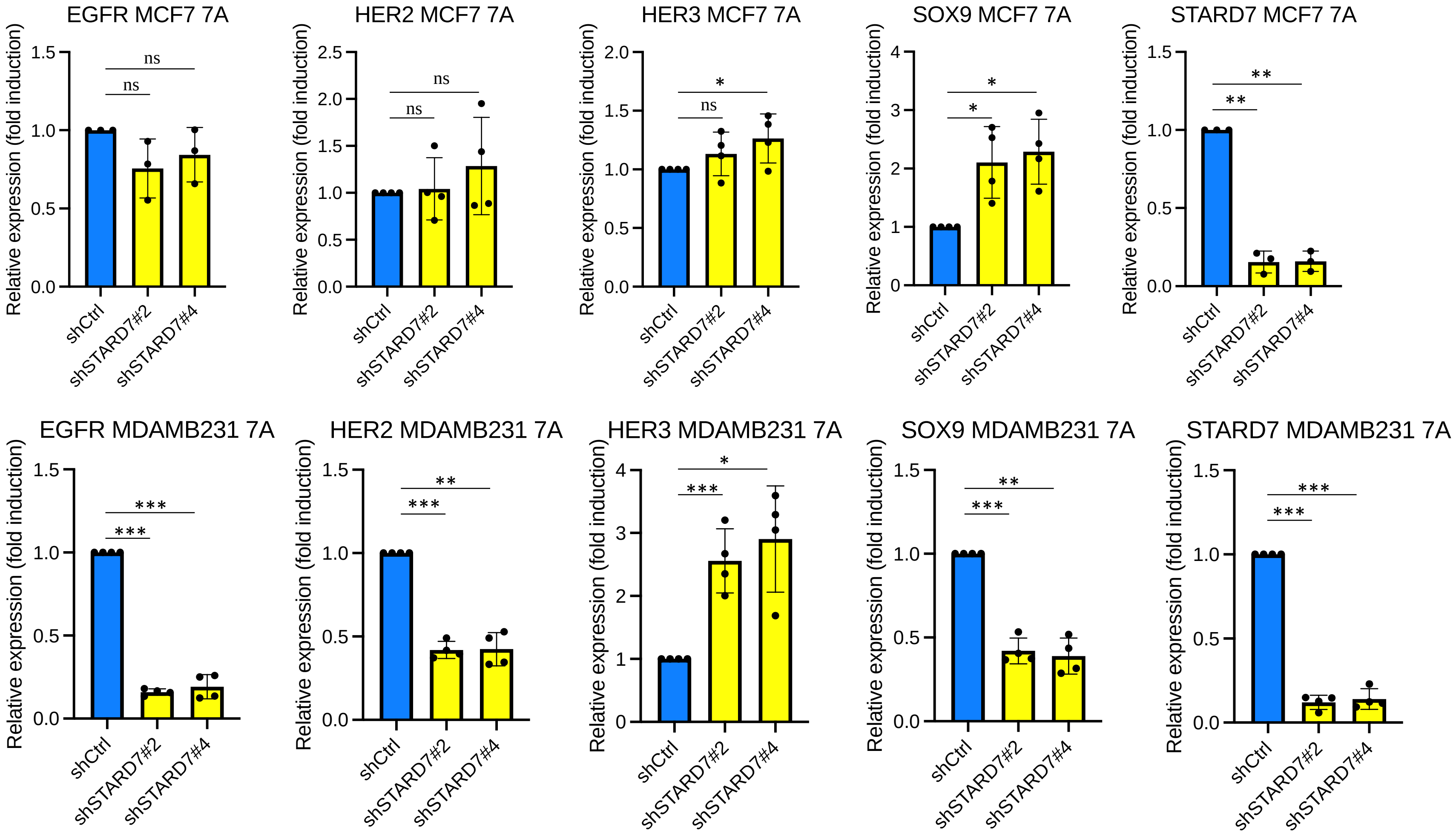
EGFR MCF7 7A
HER2 MCF7 7A
HER3 MCF7 7A
SOX9 MCF7 7A
STARD7 MCF7 7A
EGFR MDAMB231 7A
HER2 MDAMB231 7A
HER3 MDAMB231 7A
SOX9 MDAMB231 7A
STARD7 MDAMB231 7A
Relative expression (fold induction)
shCtrl
shSTARD7#2
shSTARD7#4
ns
*
**
***

Fig.7B

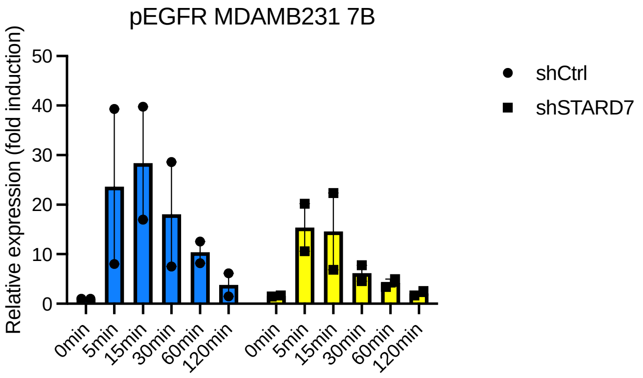

Two-way ANOVA Subject Factor p=0,0114 (*)

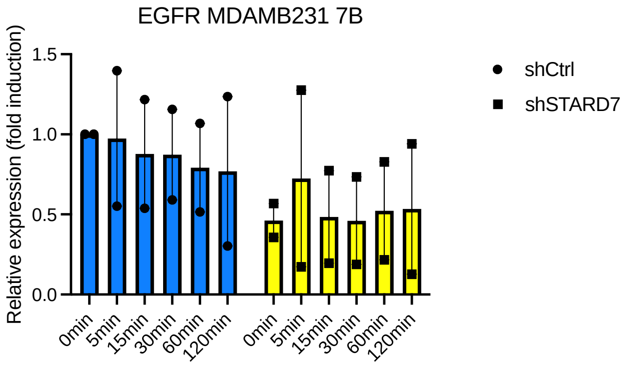

Two-way ANOVA Subject Factor p=0,0002 (***)

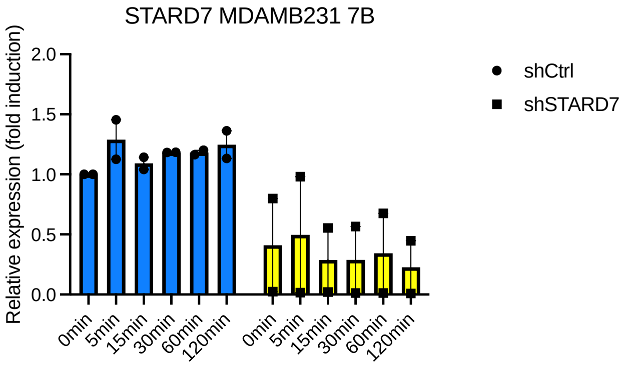

Two-way ANOVA Subject Factor p p<0,0001 (***)

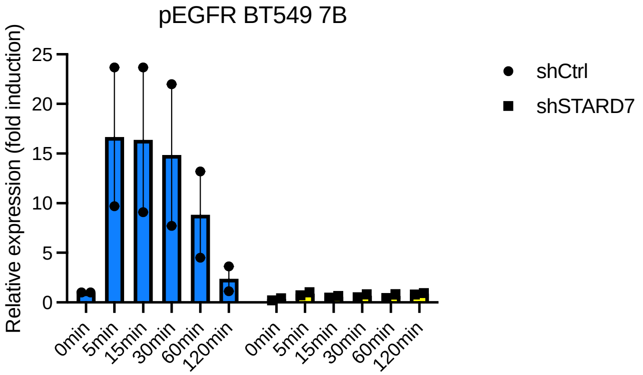

Two-way ANOVA Subject Factor p=0,0024 (**)

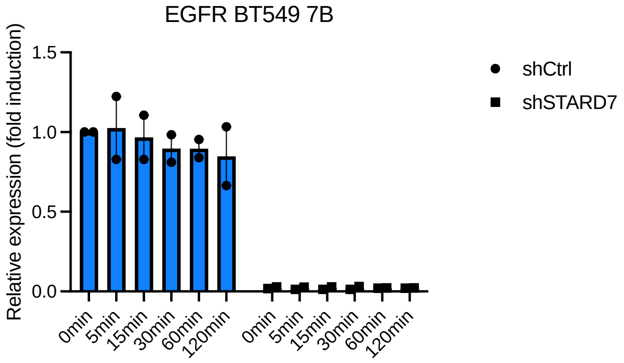

Two-way ANOVA Subject Factor p=0,0019 (**)

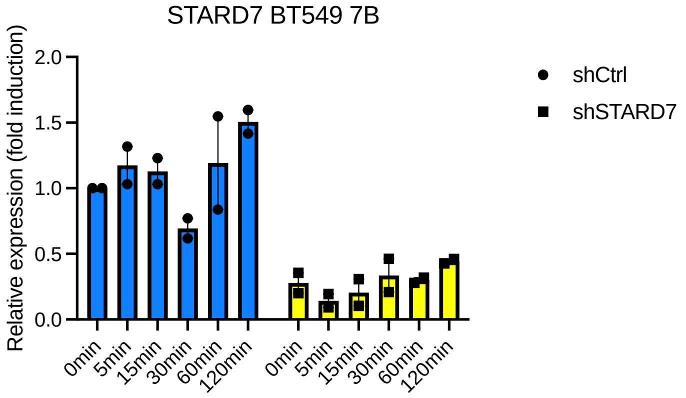

Two-way ANOVA Subject Factor p=0,0174 (*)

Fig.7C

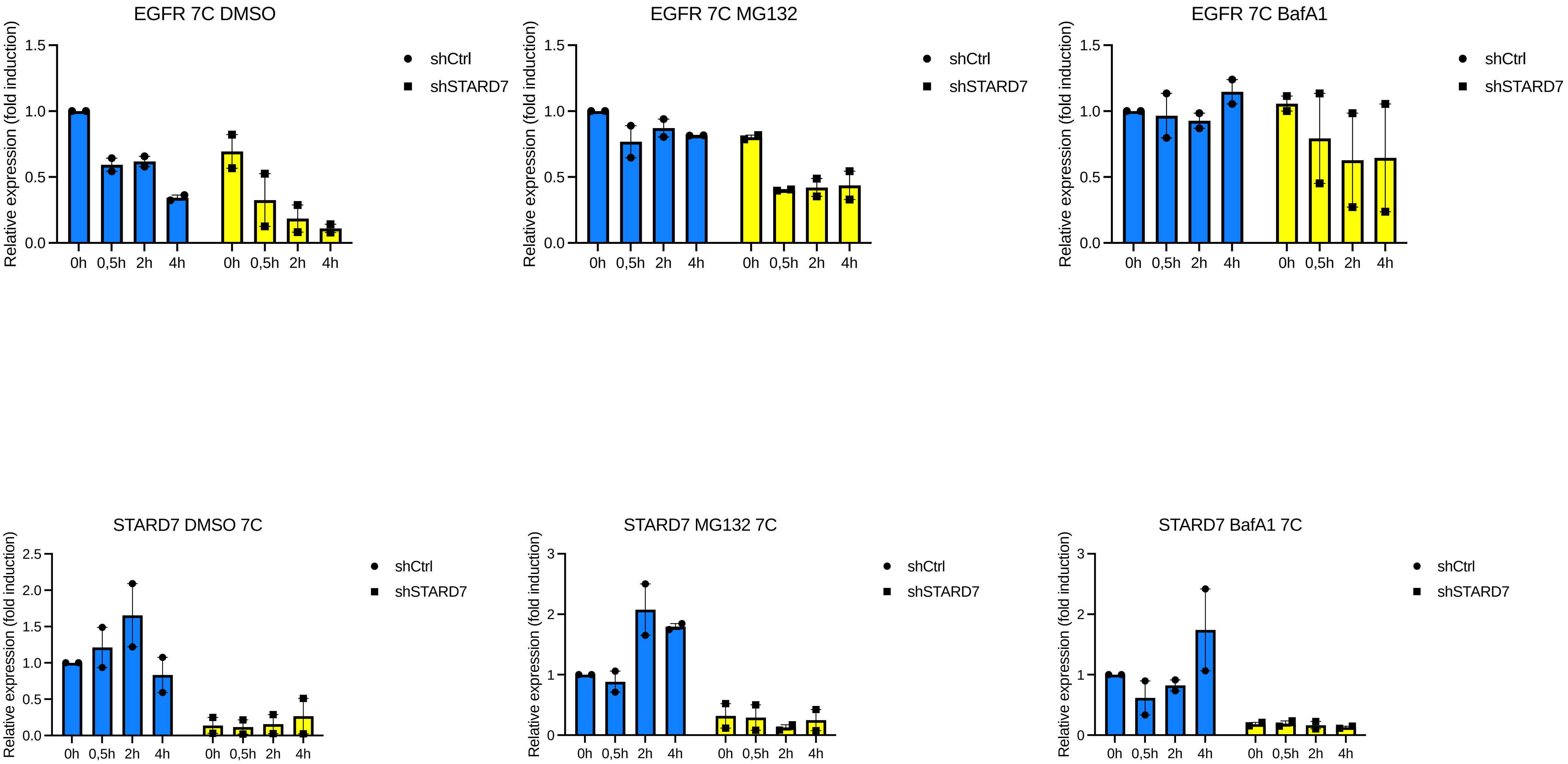
EGFR 7C DMSO
EGFR 7C MG132
EGFR 7C BafA1
STARD7 DMSO 7C
STARD7 MG132 7C
STARD7 BafA1 7C
Relative expression (fold induction)
0h
0,5h
2h
4h
shCtrl
shSTARD7
0.0
0.5
1.0
1.5
2.0
2.5
0
1
2
3

Fig.8A

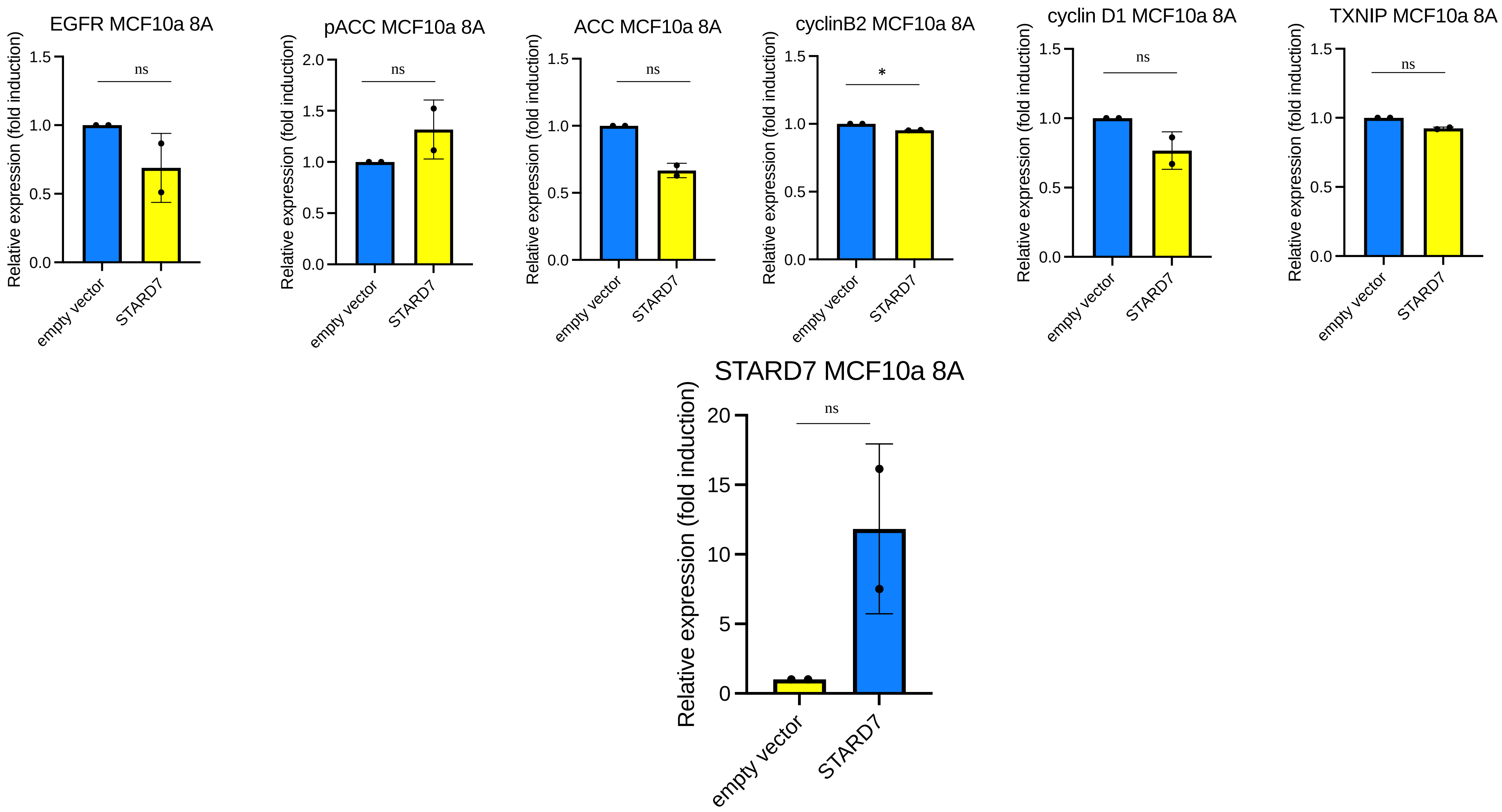
EGFR MCF10a 8A
pACC MCF10a 8A
ACC MCF10a 8A
cyclinB2 MCF10a 8A
cyclin D1 MCF10a 8A
TXNIP MCF10a 8A
STARD7 MCF10a 8A
Relative expression (fold induction)
empty vector
STARD7
ns
*
0.0
0.5
1.0
1.5
2.0
0
5
10
15
20

Fig.8B

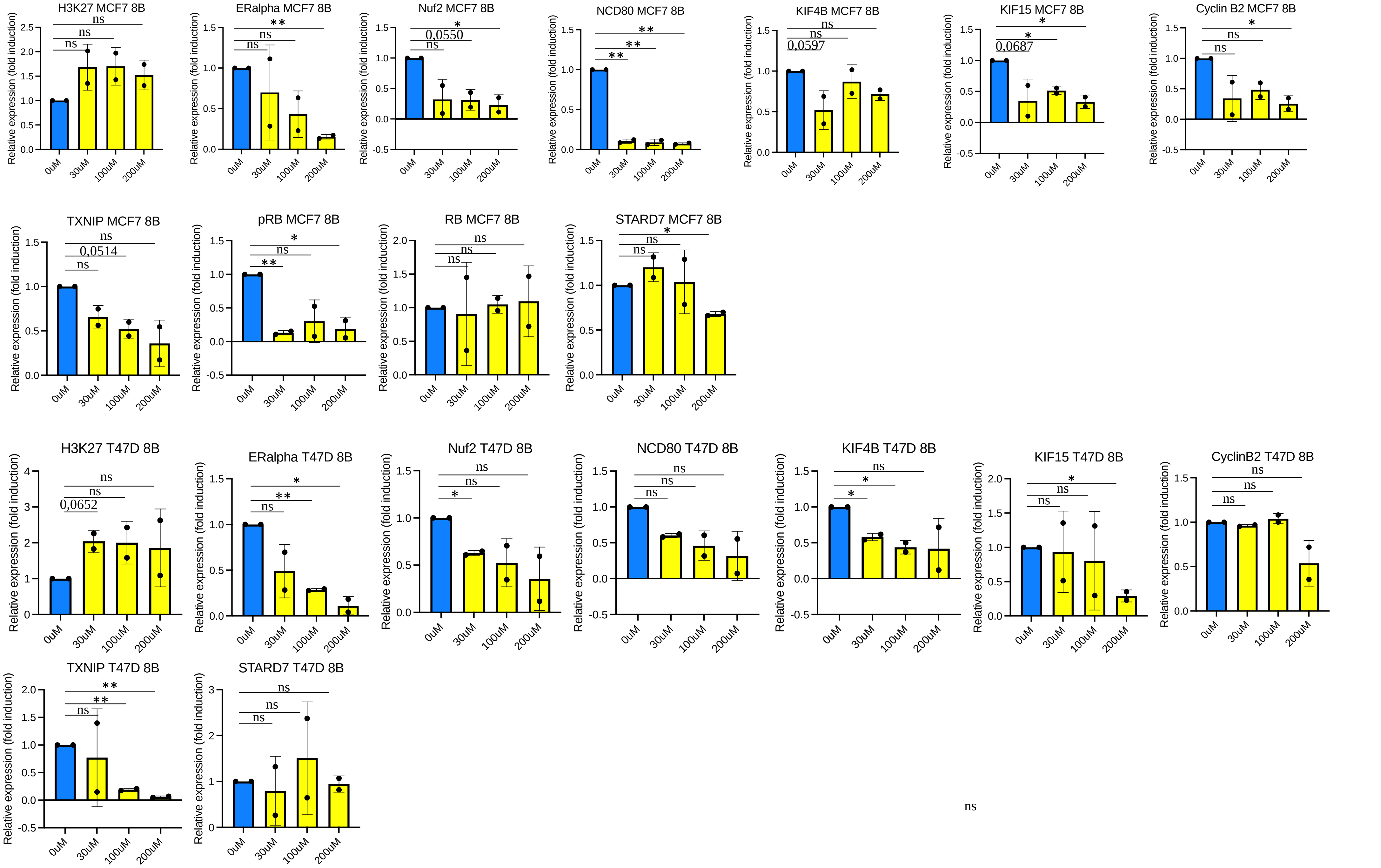
H3K27 MCF7 8B
ERalpha MCF7 8B
Nuf2 MCF7 8B
NCD80 MCF7 8B
KIF4B MCF7 8B
KIF15 MCF7 8B
Cyclin B2 MCF7 8B
TXNIP MCF7 8B
pRB MCF7 8B
RB MCF7 8B
STARD7 MCF7 8B
H3K27 T47D 8B
ERalpha T47D 8B
Nuf2 T47D 8B
NCD80 T47D 8B
KIF4B T47D 8B
KIF15 T47D 8B
CyclinB2 T47D 8B
TXNIP T47D 8B
STARD7 T47D 8B
Relative expression (fold induction)
0uM
30uM
100uM
200uM
ns
0,0550
0,0597
0,0687
0,0514
0,0652

Fig.8C

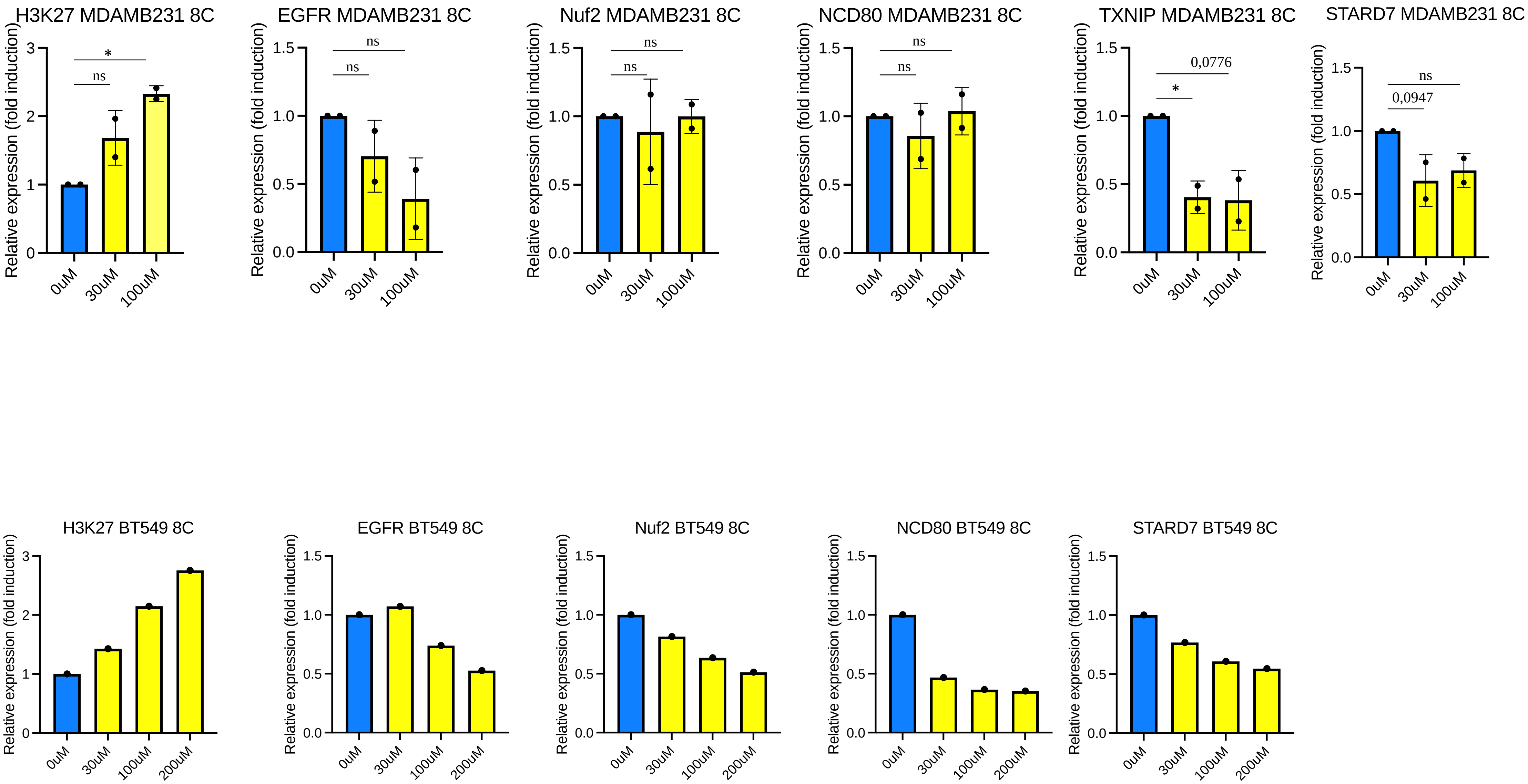
H3K27 MDAMB231 8C
EGFR MDAMB231 8C
Nuf2 MDAMB231 8C
NCD80 MDAMB231 8C
TXNIP MDAMB231 8C
STARD7 MDAMB231 8C
Relative expression (fold induction)
0uM
30uM
100uM
200uM
ns
*
0,0776
0,0947
H3K27 BT549 8C
EGFR BT549 8C
Nuf2 BT549 8C
NCD80 BT549 8C
STARD7 BT549 8C

Fig.10B

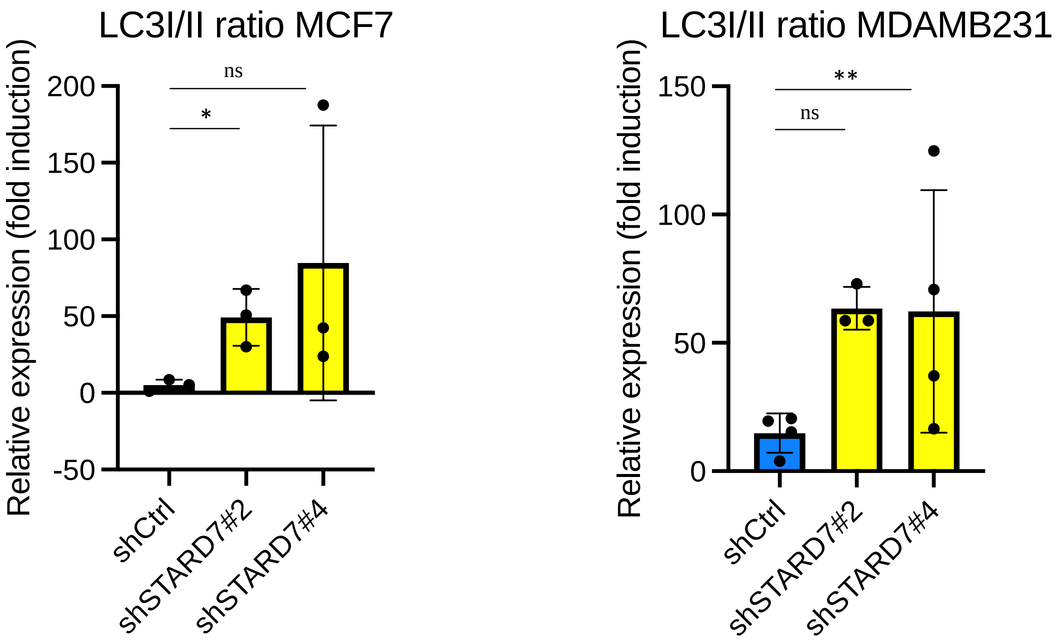
LC3I/II ratio MCF7
Relative expression (fold induction)
200
150
100
50
0
-50
ns
*
shCtrl
shSTARD7#2
shSTARD7#4
LC3I/II ratio MDAMB231
Relative expression (fold induction)
150
100
50
0
**
ns
shCtrl
shSTARD7#2
shSTARD7#4

Fig.10C

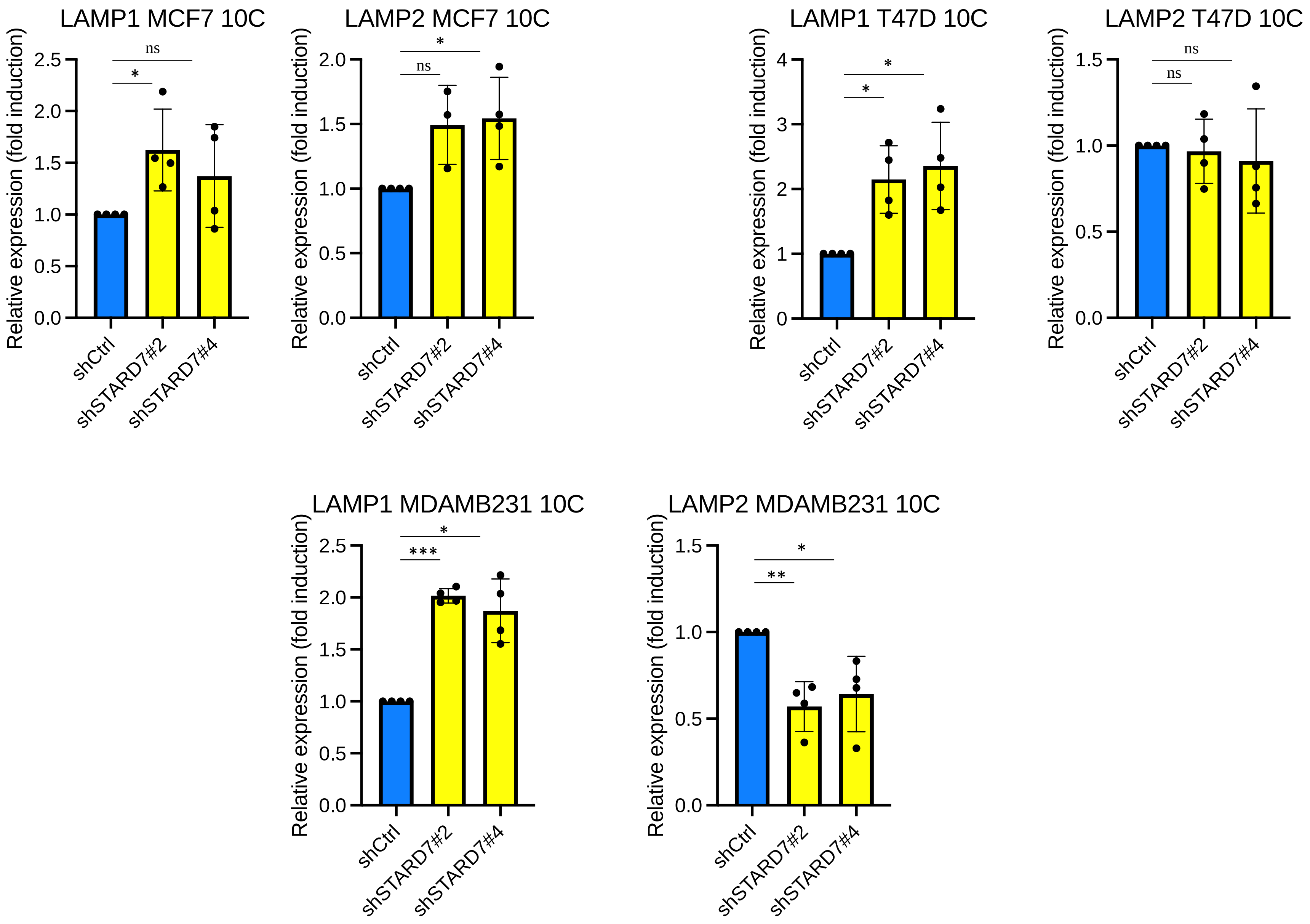
LAMP1 MCF7 10C
LAMP2 MCF7 10C
LAMP1 T47D 10C
LAMP2 T47D 10C
LAMP1 MDAMB231 10C
LAMP2 MDAMB231 10C
Relative expression (fold induction)
shCtrl
shSTARD7#2
shSTARD7#4
ns
*
**
***
