# Supplementary material for: Loss of STARD7 Triggers Metabolic Reprogramming and Cell Cycle Arrest in Breast Cancer
Source: Adv Sci (Weinh). 2025 May 30;12(31):e03022. doi: 10.1002/advs.202503022 (PMC12376514; doi:10.1002/advs.202503022)
Supplement: Supplementary file 4 — List of abbreviations. [file ADVS-12-e03022-s001.docx]

**ACC** - Acetyl-CoA carboxylase

**AKT** – Protein Kinase B

**Arl13B** - ADP-Ribosylation Factor-Like GTPase 13B

**ATF4** – Activating Transcription Factor 4

**ATP** – Adenosine Triphosphate

**BafA1** – Bafilomycin A1

**BIP** – Binding Immunoglobulin Protein

**BRCA** – Breast Invasive Carcinoma

**CA12** - Carbonic Anhydrase 12

**CCNB1** - Cyclin B1

**CCNB2** - Cyclin B2

**CCND1** - Cyclin D1

**CENPE** - Centromere Protein E

**CESC** – Cervical Squamous Cell Carcinoma and Endocervical Adenocarcinoma

**CFAP69** - Cilia and Flagella Associated Protein 69

**CHCHD10** - Coiled-Coil-Helix-Coiled-Coil-Helix Domain Containing 10

**ChIP** – Chromatin Immunoprecipitation

**CHOL** – Cholangiocarcinoma

**CHX** – Cycloheximide

**cMYC** - Cellular Myc (also known as MYC)

**CoQ** – Coenzyme Q

**COX4** – Cytochrome c oxidase subunit 4

**CPTI** - Carnitine palmitoyltransferase I

**DLBCL** – Diffuse Large B Cell Lymphoma

**DMSO** - Dimethyl Sulfoxide

**DNAAF4** - Dynein Axonemal Assembly Factor 4

**DNAH1** - Dynein Axonemal Heavy Chain 1

**DRP1** – Dynamin-Related Protein 1

**E2** - 17β-estradiol

**EEA1** - Early Endosome Antigen 1

**EGF** – Epidermal Growth Factor

**EGFR** - Epidermal Growth Factor Receptor

**EIF2α** – Eukaryotic Initiation Factor 2 alpha

**ER+** – Estrogen Receptor Positive

**ErbB** - Erythroblastic Leukemia Viral Oncogene Homolog (family of receptors)

**ERBB2** – Erb-B2 Receptor Tyrosine Kinase 2 (also known as HER2)

**ERα** – Estrogen Receptor alpha

**ESR1** - Estrogen Receptor 1

**EZH1/2** - Enhancer of Zeste Homolog 1/2

**FA** – Fatty Acids

**FIS1** – Mitochondrial Fission 1

**GBM** – Glioblastoma

**GREB1** - Growth Regulation by Estrogen in Breast Cancer 1

**GRP75** – Glucose-Regulated Protein 75

**GSEA** – Gene Set Enrichment Analysis

**H3K27** – Histone H3 Lysine 27

**HCC** – Hepatocellular Carcinoma

**HER2** - Human Epidermal Growth Factor Receptor 2

**HER3** - Human Epidermal Growth Factor Receptor 3

**IFT22** - Intraflagellar Transport 22

**IFT43** - Intraflagellar Transport 43

**IGFBP4** - Insulin-like Growth Factor Binding Protein 4

**IP3R** – Inositol 1,4,5-Trisphosphate Receptor

**IRE1α** – Inositol-Requiring Enzyme 1 alpha

**KIF15** - Kinesin Family Member 15

**KIF4B** - Kinesin Family Member 4B

**LAMP1** – Lysosomal Associated Membrane Protein 1

**LAMP2** - Lysosomal-associated Membrane Protein 2

**LC3B** - Microtubule-associated Protein 1A/1B-light Chain 3 Beta

**LC-MS** - Liquid Chromatography – Mass Spectrometry

**MAM** – Mitochondria Associated Membrane

**mTOR** – Mechanistic Target of Rapamycin

**MYB** - MYB Proto-Oncogene

**MYBL1** - MYB Like Protein 1

**NAGLU** - N-acetylglucosamine-6-sulfatase

**NASH** – Non-Alcoholic Steatohepatitis

**NDC80** - NDC80 Complex Component (also known as HEC1)

**NPC2** - Niemann-Pick C2 Protein

**NUF2** - Nucleolar and Ubiquitous Fusion 2

**p21** - Cyclin-dependent Kinase Inhibitor 1A (also known as CDKN1A)

**p27** - Cyclin-dependent Kinase Inhibitor 1B (also known as CDKN1B)

**PAAD** – Pancreatic Adenocarcinoma

**PARL** – Presenilin-Associated Rhomboid-Like

**PC** – Phosphatidylcholine

**PE** – Phosphatidylethanolamine

**PEMT** - Phosphatidylethanolamine N-methyltransferase

**PERK** – PKR-like ER Kinase

**PI3K** – Phosphoinositide 3-Kinase

**PLA2G3** - Phospholipase A2 Group III

**PTPIP51** - Protein Tyrosine Phosphatase Interacting Protein 51

**RAB11** – Ras-Related Protein Rab-11A

**RARA** - Retinoic Acid Receptor Alpha

**READ** – Rectum Adenocarcinoma

**SAM** – S-Adenosyl-L-methionine

**SERCA2b** – Sarcoplasmic/Endoplasmic Reticulum Calcium ATPase 2b

**SKMC** – Skin Cutaneous Melanoma

**SOX9** - SRY (Sex Determining Region Y)-Box 9

**STARD7** – StAR-Related Lipid Transfer Domain Containing 7

**START** – Steroidogenic acute Regulatory protein-related lipid Transfer domain

**TCGA** – The Cancer Genome Atlas

**TGCT** – Testicular Germ Cell Tumor

**THYM** – Thymoma

**TMRE** – Tetramethyl rhodamine, Ethyl Ester

**TNBC** – Triple Negative Breast Cancer

**TRXR1** - Thioredoxin Reductase 1

**TXNIP** - Thioredoxin Interacting Protein

**UPR** – Unfolded Protein Response

**UTR** – Untranslated region

**VAPB** - Vesicle-associated Membrane Protein-associated Protein B

**VDAC1** – Voltage-Dependent Anion Channel 1

**WDR19** - WD Repeat Domain 19

**XBP1** – X Box-Binding Protein 1
